# Collective behavior and virulence arsenal of the fish pathogen *Piscirickettsia salmonis* in the biofilm realm

**DOI:** 10.3389/fcimb.2022.1067514

**Published:** 2022-12-05

**Authors:** Héctor A. Levipan, Rute Irgang, L. Felipe Opazo, Henry Araya-León, Ruben Avendaño-Herrera

**Affiliations:** ^1^ Laboratorio de Ecopatología y Nanobiomateriales, Departamento de Ciencias y Geografía, Facultad de Ciencias Naturales y Exactas, Universidad de Playa Ancha, Valparaíso, Chile; ^2^ Centro de Espectroscopía Atómica y Molecular (ATMOS-C), Universidad de Playa Ancha, Valparaíso, Chile; ^3^ Laboratorio de Patología de Organismos Acuáticos y Biotecnología Acuícola, Facultad de Ciencias de la Vida, Universidad Andrés Bello, Viña del Mar, Chile; ^4^ Interdisciplinary Center for Aquaculture Research (INCAR), Universidad Andrés Bello, Viña del Mar, Chile; ^5^ Institute of Ecology and Biodiversity (IEB), Santiago, Chile; ^6^ Departamento de Ecología, Facultad de Ciencias Biológicas, Pontificia Universidad Católica de Chile, Santiago, Chile; ^7^ Centro de Investigación Marina Quintay (CIMARQ), Universidad Andrés Bello, Quintay, Chile

**Keywords:** piscirickettsiosis, RNA sequencing, biofilm cell viability, exopolymeric matrix, cytotoxicity, aquaculture, virulence

## Abstract

Piscirickettsiosis is a fish disease caused by the Gram-negative bacterium *Piscirickettsia salmonis.* This disease has a high socio-economic impact on the Chilean salmonid aquaculture industry. The bacterium has a cryptic character in the environment and their main reservoirs are yet unknown. Bacterial biofilms represent a ubiquitous mechanism of cell persistence in diverse natural environments and a risk factor for the pathogenesis of several infectious diseases, but their microbiological significance for waterborne veterinary diseases, including piscirickettsiosis, have seldom been evaluated. This study analyzed the *in vitro* biofilm behavior of *P*. *salmonis* LF-89^T^ (genogroup LF-89) and CA5 (genogroup EM-90) using a multi-method approach and elucidated the potential arsenal of virulence of the *P*. *salmonis* LF-89^T^ type strain in its biofilm state. *P*. *salmonis* exhibited a quick kinetics of biofilm formation that followed a multi-step and highly strain-dependent process. There were no major differences in enzymatic profiles or significant differences in cytotoxicity (as tested on the Chinook salmon embryo cell line) between biofilm-derived bacteria and planktonic equivalents. The potential arsenal of virulence of *P*. *salmonis* LF-89^T^ in biofilms, as determined by whole-transcriptome sequencing and differential gene expression analysis, consisted of genes involved in cell adhesion, polysaccharide biosynthesis, transcriptional regulation, and gene mobility, among others. Importantly, the global gene expression profiles of *P*. *salmonis* LF-89^T^ were not enriched with virulence-related genes upregulated in biofilm development stages at 24 and 48 h. An enrichment in virulence-related genes exclusively expressed in biofilms was also undetected. These results indicate that early and mature biofilm development stages of *P*. *salmonis* LF-89^T^ were transcriptionally no more virulent than their planktonic counterparts, which was supported by cytotoxic trials, which, in turn, revealed that both modes of growth induced important and very similar levels of cytotoxicity on the salmon cell line. Our results suggest that the aforementioned biofilm development stages do not represent hot spots of virulence compared with planktonic counterparts. This study provides the first transcriptomic catalogue to select specific genes that could be useful to prevent or control the (*in vitro* and/or *in vivo*) adherence and/or biofilm formation by *P*. *salmonis* and gain further insights into piscirickettsiosis pathogenesis.

## Introduction

The term ‘biofilm’ was first coined to describe bacterial attachment on submerged solid surfaces in a wastewater treatment system (Mack et al., 1975), with later expansion to the area of health (Nickel et al., 1985). Today, the canonical definition of “biofilm” is under debate (Flemming et al., 2021), as driven by the now existent body of knowledge (Høiby, 2017; Penesyan et al., 2021). Biofilms often proliferate on (but are not limited to) solid interfaces, there gaining emergent properties that are seldom observed in free-living and recently biofilm-detached bacteria (Rumbaugh and Sauer, 2020). Such properties have medical significance, contributing to an enhanced antimicrobial tolerance, expedited bacterial signaling, facilitated horizontal gene transfer, and a promoted expression of virulence factors (Flemming et al., 2016). However, the veterinary significance of biofilms for fish health is still widely speculative and has thus far been scarcely supported by scientific knowledge since crosstalk between aquatic diseases and biofilm microbiology has received little attention.

An example of this sanitary landscape is the relationship between piscirickettsiosis, or Salmon Rickettsial Septicaemia (SRS), and biofilms produced by the etiological agent *Piscirickettsia salmonis* (Avendaño-Herrera et al., 2022). This microorganism is a Gram-negative facultative intracellular γ-proteobacteria ranked among the most important bacterial pathogens for the Chilean salmon industry; this status means *P*. *salmonis* is a primary target for antimicrobials (Avendaño-Herrera et al., 2022). Moreover, *P*. *salmonis* has successfully evaded the Chilean vaccination regimens due to multiple factors (Happold et al., 2020), such as host-intrinsic genetic variations (Figueroa et al., 2020). Despite accumulated knowledge about piscirickettsiosis through the surveillance program of the National Fisheries and Aquaculture Service of Chile (SERNAPESCA) (Gaete-Carrasco et al., 2019; Avendaño-Herrera et al., 2022), *P*. *salmonis* remains particularly detrimental to the marine farming stage of the salmon industry.

Rozas-Serri (2022) proposed an interesting conceptual framework for understanding the infectious setting of piscirickettsiosis – *P*. *salmonis* mainly exploits skin and gill pathways to invade fish hosts. Later, adhered bacteria can develop microcolony-like aggregates *in vivo* on the surface of the external epithelial cells of the skin and gills of fish host, as well as on goblet and skeletal muscular cells (Smith et al., 2015). Moreover, *P*. *salmonis* forms biofilms *in vitro* on different abiotic substrates, including *Mytilus chilensis* mussels (Larenas et al., 2019); *M*. *chilensis* is the second species after salmons in commercial importance in Chile, and mussel production takes place in salmon-farming areas, thus potentially acting as an environmental reservoir for the bacterium (Hariharan and Amadi, 2016). Remarkably, despite the recognized role of fish skin mucus as the first immunological barrier in fighting waterborne pathogens (Esteban and Cerezuela, 2015), *P*. *salmonis* cells in the biofilm exopolysaccharide matrix maintain their cell viability in seawater after exposure to high concentrations of salmon skin mucus (Levipan et al., 2020; Levipan and Avendaño-Herrera, 2021). The ability of *P*. *salmonis* to form biofilms is associated with “piscirickettsial attachment complex” formation, which may also favor the vertical transmission of piscirickettsiosis when the bacterium adheres on fish eggs (Larenas et al., 2003). While biofilms are potentially important environmental reservoirs of diseases (Marshall et al., 2012; Levipan et al., 2020), exhaustive field experimentation is needed to test if these microbial accretions act as seedbanks (Zhang et al., 2019) for the vector-free waterborne propagation of *P*. *salmonis* (Bravo et al., 2020; Long et al., 2021).

During the intracellular mode of growth, *P*. *salmonis* actives virulence genes (e.g., *dot*/*icm* homologue genes, protease-encoding genes, etc.) (Gómez et al., 2013; Labra et al., 2016; Figueroa et al., 2021) and generates several proteins (Ortiz-Severín et al., 2020) with virulence properties; these features can co-occur with a generalized translational arrest (Zúñiga et al., 2020). Some of these activated genes (e.g., *clpB*, *bipA*, *dot*/*icm*) participate in intracellular survival (Isla et al., 2014; Machuca and Martinez, 2016) or biofilm-formation regulation (e.g., stringent response genes) (Díaz-Salazar et al., 2017; Zúñiga et al., 2020), with intracellular infection state-dependent levels of gene expression (Ortiz-Severín et al., 2021). Similarly, expression analysis of specific genes in *P*. *salmonis* indicates the activation of survival-related genes under conditions that favor biofilm development, such as the toxin/anti-toxin *mazEF* operon (Marshall et al., 2012) and *cheA* gene (Albornoz et al., 2017). However, the extent to which global patterns in gene expression of *P*. *salmonis* are shared between the intracellular and biofilm modes of growth remains unclear. A similar knowledge gap exists between the biofilm and planktonic modes of growth of *P*. *salmonis* regarding the expression of virulence-related genes and resulting virulent phenotypes. This latter topic is relevant as bacterial biofilms can exhibit a super infectious phenotype, which can trigger infections at doses lower (by orders of magnitude) than the planktonic equivalents (Tamayo et al., 2010).

Gross virulence for intracellular fish pathogens can be estimated from the cytotoxic response of cell lines by quantifying lactate dehydrogenase (LDH) release. Differences were nonexistent between biofilm-derived and planktonic bacteria of *P*. *salmonis* regarding LDH release in the salmon head kidney cell line (SHK-1) during early states of infection (i.e., ≤ 10 days post-infection) (Levipan et al., 2020; Santibañez et al., 2020). However, the cytotoxic response of SHK-1 cells, measured as the expression levels of proinflammatory genes in the first 24 h, indicated a strain-dependent association with the bacterial mode of growth (i.e., biofilm versus planktonic) (Santibañez et al., 2020). Thus far, no clear evidence supports a straightforward link between biofilm formation (as a putative pathogenic mechanism) and the pathogenesis of piscirickettsiosis. Compared to the expression analysis of specific traits and genes (e.g., Marshall et al., 2012; Albornoz et al., 2017), whole transcriptome sequencing brings more holistic insights into the virulence potential of *P*. *salmonis* biofilms. Indeed, depending on which species is studied and the methodological approaches used, generalizations on the potential hypervirulence of biofilms can differ markedly among bacterial pathogens. For instance, transcriptomic evidence in pathogenic marine and freshwater bacteria, such as *Vibrio tapetis* (Rodrigues et al., 2018), *Flavobacterium psychrophilum* (Levipan and Avendaño-Herrera, 2017), and *Flavobacterium columnare* (Lange et al., 2018), indicates higher levels of virulence for biofilm states compared with planktonic bacteria. However, other bacterial models using the same technique can result in an opposite trend (Charlebois et al., 2016).

The aims of this study were to characterize *in vitro* biofilm formation by *P*. *salmonis* LF-89^T^ and CA5 strains using a multi-method approach and to establish the potential arsenal of virulence of biofilm-derived bacteria. *P. salmonis* LF-89^T^ is the representative strain of the LF-89 genogroup, a cluster that, in general, contains isolates characterized by a higher and ubiquitous prevalence, wider host range, and higher antibiotic resistance than members of the EM-90 genogroup (Saavedra et al., 2017). Precisely due to this point, our study included strain *P. salmonis* CA5, a representative of the EM-90 genogroup, for comparative purposes. Both strains showed fast kinetics of biofilm formation in a highly strain-dependent multi-step process. We hypothesized that global differences in gene expression between biofilm and planktonic states involve an important number of virulence-related genes upregulated in the biofilm mode of growth, as well as an important number of virulence-related genes only expressed in biofilms. Whole-transcriptome analysis of *P*. *salmonis* LF-89^T^ revealed global gene expression patterns with a scarce contribution of virulence-related genes upregulated in 24 h and 48 h biofilms. Moreover, a small number of virulence-related genes were exclusively expressed in biofilms. Our results suggest that early and mature biofilm development states are not hot spots of virulence as compared to the respective planktonic equivalents.

## Materials and methods

### Strains and growth conditions

*Piscirickettsia salmonis* type strain LF-89^T^ (ATCC VR-1361) and the field Chilean strain CA5 were studied. These strains have been previously identified as belonging to the LF-89 and EM-90 genogroups, respectively. The field strain was isolated in 2012 from the liver of a clinically infected Atlantic salmon (*Salmo salar*) during a piscirickettsiosis outbreak affecting a Chilean farm in the Aysén Region. The two strains were confirmed as *P*. *salmonis* by standard phenotyping and routine internal transcribed spacer-based PCR analysis (Marshall et al., 1998). The strains were routinely cultured at 18°C for 4-5 days in Austral-TSHem agar plates (Yáñez et al., 2013) and agitated-liquid AUSTRAL-SRS medium at 120 rpm (Yáñez et al., 2012). The strains were stored in Cryobille tubes for long-term storage at -80°C, as per the manufacturer’s instructions (AES Laboratory). The purity of the strains was corroborated by Gram staining and optical microscopy prior to beginning experiments.

### Specific biofilm formation

Once the two *P*. *salmonis* strains were routinely grown in AUSTRAL-SRS medium, they were then inoculated into 96-well microplates (flat-bottom, SPL Life Sciences Co., Ltd.) to determine the specific biofilm formation (SBF) index using the crystal violet (CV) method. Briefly, inocula were adjusted to ~0.8 McFarland and sizes were determined by counting the number of colony-forming units (CFU) on Austral-TSHem agar plates (incubated as described above) by the standard serial-dilution method. Later, microplate wells were inoculated with aliquots of 150 μL of AUSTRAL-SRS medium (8 wells per inoculum) containing 7.86 ± 6.06 × 10^6^ (i.e., 0.81 ± 0.02 McFarland) and 3.42 ± 1.61 × 10^6^ CFU mL^-1^ (i.e., 0.81 ± 0.01 McFarland) of *P*. *salmonis* LF-89^T^ and CA5, respectively. Similarly, eight wells per microplate were inoculated with 150 μL of sterile AUSTRAL-SRS broth as negative controls. Three independent experiments were performed by inoculating thirteen 96-well microplates in each experiment. Inoculated microplates were statically incubated at 18°C until further individual processing at 24 h intervals to evaluate biofilm development over a 13-day total period. After incubation, inoculated and control wells were emptied and washed (3X) with 200 μL of sterile milli-Q water and then stained with 180 μL of CV solution (1% w/v) at room temperature for 15 min. Afterward, the CV solution was discarded, and the wells were washed with abundant tap water until no more dye was released. The microplate was turned upside down to dry at room temperature for 15 min before adding 180 μL of absolute ethanol per well for CV solubilization at room temperature for 15 min. The SBF index was determined by reading absorbance of the EtOH-solubilized CV at 585 nm using a Tecan Microplate Reader (Infinite 200 PRO, Männedorf, Switzerland) and computed in accordance with Niu and Gilbert (2004) as (B - NC)/G, where B is the amount of EtOH-solubilized CV released from biofilms; NC is the amount of EtOH-solubilized CV that adhered to the microplate surfaces in negative controls; and G is the absorbance of bacterial supernatants at 620 nm.

### Physiological and morphological characterization of *P*. *salmonis* biofilms

The physiological characteristics for 48-h-old biofilms were determined using the miniaturized API ZYM system in accordance with the manufacturer’s directions (API-BioMerieux, La Balmeles-Grottes, France), but with two modifications regarding the incubation temperature (at 18°C) and the time elapsed between inoculation and revealing (4 to 5 days). Briefly, 48-h-old biofilms were formed on the bottom of sterile glass Petri dishes by inoculating 20 mL of AUSTRAL-SRS medium containing *P*. *salmonis* LF-89^T^ and CA5 at ~1 × 10^6^ CFU mL^-1^. Once biofilms were established, the supernatants were withdrawn to wash (3X) sessile bacteria and discard non-adherent ones with 10 mL of chilled sterile NaCl solution (2.5% w/v). A washed Petri dish of each strain was randomly selected for staining with CV (1% w/v) at room temperature for 10 min. Once the dye was discarded, CV-stained Petri dishes were washed with tap water (until no more dye was released) and dried at room temperature for 15 min (**Supplementary Figure S1A**) to confirm the formation of biofilms under an optic microscope at 1,000X magnification (**Supplementary Figure S1B**). The remaining washed Petri dishes were used to prepare 48-h-old biofilm inocula by resuspending the sessile bacteria from the bottoms by using cell scrapers and 5 mL of saline solution (2.5% NaCl). To verify the biofilm detachment procedure, some scraped Petri dishes were randomly selected for CV (1% w/v) staining (**Supplementary Figure S1C**) and optical microscopy (**Supplementary Figure S1D**). Before assays, biofilm inocula of *P*. *salmonis* LF-89^T^ and CA5 were adjusted to 6.0 McFarland equivalent to 1.40 ± 0.87 × 10^8^ CFU mL^-1^. Planktonic inocula of both strains were harvested by centrifugation at 9,000 rpm and 4°C for 2.5 min from culture supernatants, and the resulting pellets were washed (3X) with a sterile saline solution and resuspended in the same saline solution at 1.65 ± 0.65 × 10^8^ CFU mL^-1^ for subsequent API ZYM assays for comparison purposes.

Changes in biofilm cell viability over time were determined with the LIVE/DEAD BacLight Bacterial Viability Kit (Invitrogen, Molecular Probes, Oregon, USA); this kit was evaluated prior to use in samples. To do so, 1 mL-aliquots of fresh *P*. *salmonis* cultures (prepared in AUSTRAL-SRS medium) were fixed overnight in formaldehyde (10% v/v) to obtain dead cells. Afterward, fixed cells were harvested by centrifugation at 4°C and 10,000 rpm for 2.5 min, washed (3X) with sterile NaCl solution (2.5% w/v), and then resuspended in the same saline solution. To obtain living cells, 1 mL aliquots of fresh cultures were directly harvested by centrifugation at 4°C and 9,000 rpm for 2.5 min, washed (2X) in sterile NaCl solution, and resuspended in the same saline solution. Afterward, bacterial suspensions with different live-to-dead cell ratios (0: 100, 10: 90, 50: 50, 90:10, and 100: 0; hereafter, expected ratios) were prepared by mixing live and dead bacterial suspensions. The expected ratios of live-to-dead bacteria in bacterial suspensions were correlated with the empiric ratios of live-to-dead bacteria in the same bacterial suspensions by using the Pearson’s correlation coefficient. Empiric ratios of live-to-dead bacteria in bacterial suspensions (with different expected ratios of live-to-dead bacteria) were determined by direct cell counting on an Olympus BX41 microscope at 1,000X magnification (Olympus Corporation, Tokyo, Japan). Pearson’s correlation coefficients between expected and empiric ratios of live-to-dead bacteria indicated that the LIVE/DEAD Viability kit detected physiologically contrasting states of *P*. *salmonis* LF-89^T^ (r = 0.9995, P < 0.05) and CA5 (r = 0.9975, P < 0.05).

Three independent experiments were conducted to determine the changes in both the surface coverage and bacterial viability of biofilms formed at 18°C and at 24 h intervals over a 360 h period. The two *P*. *salmonis* strains were grown in AUSTRAL-SRS medium and, prior to conducting the experiments, inoculum sizes were adjusted to 5.09 ± 0.59 × 10^6^ CFU mL^-1^ (0.79 ± 0.01 McFarland) and 3.52 ± 3.15 × 10^6^ CFU mL^-1^ (0.80 ± 0.03 McFarland) for *P*. *salmonis* LF-89^T^ and CA5, respectively. Ninety-six-well microplates were inoculated in triplicate (at 24 h intervals) with 150 µL of McFarland-adjusted inocula to monitor different stages of biofilm development (i.e., from early to advanced states of biofilm formation, including the mature stage) on a single microplate. Furthermore, triplicate 150 µL aliquots of sterile AUSTRAL-SRS medium were included as negative controls in each experiment. After incubation, inoculated and negative-control wells of microplates were emptied and washed (3X) with 200 μL of a sterile NaCl solution (2.5% w/v). Later, washed biofilms were stained with the LIVE/DEAD Kit as per the manufacturer’s instructions, except that dye salts were dissolved in a sterile NaCl solution (2.5% w/v).

Endpoint images (single-point reading) from LIVE/DEAD-stained biofilms were automatically captured on a Cytation 5 Imaging Multi-Mode Reader (BioTek Instruments Inc., Winooski, VT, USA) by using the 20X objective with the correction collar set to 0.5 mm (for the usual bottom thickness of 96-well microplates). Image capturing was carried out in two fluorescence channels; green (GFP: 469, 525) and acridine orange (AO: 469, 647). Optimal image exposure was achieved using LED intensities of 3 and 5 (for GFP and OA channels, respectively), an integration time of 10 and 35 ms (for GFP and OA channels, respectively), and a camera gain equal to zero (both channels). The laser autofocus method allowed us to find the optimal focus position using a laser beam that corrects for any irregularities in plastic. Once the trained position was found, the instrument used the described channels and settings to automatically capture the biofilms formed on the bottom of each well. In this regard, the bottom elevation (a parameter that refers to the distance in microns between the top of the microplate carrier and the bottom of the well where the sample is visible) was 3,250 microns. Additionally, endpoint fluorescence intensities were quantified for each well using the cell imaging multi-mode reader with a monochromator-based system, with excitation wavelength and bandwidth set to 485/20 nm and emission wavelengths and bandwidths set to 530/20 nm (green emission for live bacteria) and 630/20 nm (red emission for dead or damaged bacteria). The “wavelength switch per well” feature was set to “on” for sequential signal detection of both emissions before moving to the next well. Reads were performed using top fluorescence optics, a normal read speed (i.e., delay after plate movement of 100 ms), 10 measurements per data point, a height of 7 mm, and an extended dynamic range for automatic gain adjustment. Fluorescence data were corrected by the background fluorescence emitted by negative-control wells and expressed as the ratio of the green-to-red fluorescence; this approach has previously been used as a proxy to evaluate the changes in bacterial viability of biofilms formed by other strains of *P*. *salmonis* (Levipan et al., 2020).

For scan laser confocal microscopy, sterile glass slides were dipped into 20 mL of *P*. *salmonis* LF-89^T^ and CA5 cultures (prepared in AUSTRAL-SRS medium at ~1 × 10^6^ CFU mL^-1^) inside sterile glass Petri dishes. After static incubations, 48-h-old biofilms formed on glass slides were washed (3X) with a chilled sterile NaCl solution (2.5% w/v) and stained with the LIVE/DEAD Viability kit (following the manufacturer’s instructions) to determine the contribution of living and dead (or inactive) bacteria to the biofilm and the distribution of sessile bacteria on glass slides. A drop of mounting antifade oil (Invitrogen) was placed on LIVE/DEAD-stained slides and covered with a coverslip for subsequent microscopic observation on a Leica TCS SP5 II spectral confocal microscope (Leica Microsystems Inc., Jena, Germany). The images were collected using the HCX PL APO 100x/1.44 Oil CORR CS objective and the Leica Confocal software version 2.6 (Leica Inc.). In addition, 48-h-old biofilms formed on other set of glass slides were fixed in chilled methanol for 15 min, dipped once in sterile NaCl solution (2.5% w/v), air-dried at room temperature, and stained as per the manufacturer’s specifications with Alexa fluor 488-conjugated wheat germ agglutinin (Invitrogen) (5 μg mL-1) and 4’,6-diamidino-2-phenylindole (DAPI) (Sigma-Aldrich). Wheat germ agglutinin- and DAPI-stained slides were mounted using a drop of Dako mounting medium (Invitrogen) and coverslips for subsequent observation and imaging of the exopolysaccharide matrix of biofilms with embedded bacteria. The images were collected as previously described, and at least 10 microscopic fields were analyzed per sample.

### RNA extraction and RT-qPCR

RNA extraction and subsequent RNA-based experiments were carried out using *P*. *salmonis* strain LF-89^T^ considering three criteria. First, strain LF-89^T^ is the type of the species, the most studied worldwide, and is available in bacterial culture collections. This allows researchers to replicate published results as *P. salmonis* LF-89^T^ can be included for comparison when testing other field isolates. Second, this strain formed biofilms with higher levels of fluorescence of live bacteria compared with *P*. *salmonis* CA5, especially in the mature biofilm state. Finally, the increase in cytotoxicity induced by planktonic and biofilm-derived bacteria of *P*. *salmonis* LF-89^T^ was faster and sustained over time (see in the Results section). Briefly, total RNA was extracted with the TRIzol™ Max Bacterial RNA Isolation Kit (Thermo Fisher Scientific, NY, USA) from 24-h and 48-h-old biofilms of *P*. *salmonis* LF-89^T^. Biofilms were induced to form on the bottom of sterile glass Petri dishes under standard and static incubation conditions (as described before). After incubation, the resulting biofilms were washed (3X) with 10 mL of chilled sterile NaCl solution (2.5% w/v) to discard non-adherent cells and then scraped off with a cell scraper in 1 mL of TRIzol™ reagent. Three independent experiments were conducted, and, in each, four Petri dishes with biofilms were scraped using the same milliliter of TRIzol™ reagent. This 1 mL-suspension was later used for RNA extraction according to the manufacturer’s specifications. Complete cell detachment was confirmed by microscopic observation of TRIzol-scraped Petri dishes that were randomly chosen for CV (1% w/v) staining (**Supplementary Figure S1E**) and optical microscopy (**Supplementary Figure S1F**). For the sake of comparison, 1 mL aliquots of culture supernatant (containing planktonic bacteria) were collected from randomly selected glass Petri dishes in each independent experiment. Planktonic bacteria were harvested by centrifugation at 10,000 rpm and 4°C for 2.5 min, and each bacterial pellet was resuspended in 1 mL of TRIzol™ reagent for RNA extraction per the manufacturer’s instructions.

The RNA integrity number for each RNA extract was determined on an Agilent 2100 Bioanalyzer instrument using the RNA 6000 Nano kit in accordance with the manufacturer’s instructions (Agilent Technologies, CA, USA). Four total RNA extracts were obtained in triplicate with an RNA integrity number ≥ 7.0; the RNA extracts were quantified on a QuantiFluor RNA System using the instructions provided by the manufacturer (Promega, WI, USA). The RNA concentration was equal to 127 ± 23 μg μL^-1^ and 116 ± 26 μg μL^-1^ for 24-h and 48-h-old biofilm samples, respectively. In turn, measured RNA concentrations for 24-h and 48-h-old planktonic samples were 87 ± 30 μg μL^-1^ and 81 ± 36 μg μL^-1^, respectively. All RNA extracts were stored at -20°C until further use in both RNA sequencing and validation *via* RT-qPCR of the resulting transcriptomes.

For the sake of validating the transcriptomic data, 10 DEGs (differentially expressed genes as determined by RNA sequencing) were randomly chosen for design of qPCR primers (**Supplementary Table S1**) at Genoma Mayor Spa (http://www.genomamayor.com/) by using the Primer3 software (https://primer3.org/). To confirm specificity of the primers, a resulting amplicon from each primer pair was randomly selected for cloning with the pGEM-T Easy Vector System (Promega, Madison, CA, USA) and sequencing at Macrogen Inc. (https://dna.macrogen.com/). Two-step reverse transcription-qPCR (RT-qPCR) was performed from complementary DNA (cDNA) synthetized using the ImProm-II Reverse Transcription System (Promega), 20 ng of DNase-treated RNA (TURBO DNA-free kit, Applied Biosystems, Austin, TX, USA), and random primers from the ImProm-II System. All RT-qPCRs were performed on a Stratagene Mx3000P PCR device (Agilent Technologies, CA, USA) in a total volume of 20 μL with 1 μL of cDNA, 2X Brilliant II SYBR Green QPCR Master Mix (1X, final concentration; Agilent Technologies), forward and reverse primers (1 μM, final concentration), and ROX as a passive reference dye (18 nM, final concentration; Agilent Technologies). The description of target genes, amplicon lengths, primer sequences, and annealing temperatures are listed in **Supplementary Table S1**. The qPCR program consisted of initial denaturation for 3 min at 95°C, followed by 40 amplification cycles that consisted of denaturation at 95°C for 30 s, primer annealing for 45 s at different melting temperatures (**Supplementary Table S1**), and 45 s of extension at 72°C. Amplicons were within the expected size, as assessed by analyzing melting curves and standard electrophoresis in agarose gels.

Absolute qPCR data were processed with the MXPro software version 4.10 (Agilent Technologies). For quantification of the gene transcripts, the sequenced clones were used to construct 7-point standard curves (in triplicate) by 10-fold dilution series starting from 4 × 10^7^ copies. To do so, the concentration of each clone was determined with the QuantiFluor dsDNA System per the manufacturer’s instructions (Promega, WI, USA). Afterward, the copy number of clones was calculated by dividing the DNA concentration (in ng μL^-1^) of a given clone by its mass (in ng) estimated with the following formula: mass = [(n) × (M/NA)] × 10^9^, where n is the clone size in bp (vector plus insert); M, is the mean molecular weight of a nitrogenous bp (660 g mol^-1^); NA, is the Avogadro’s number (6.0221 × 10^23^ molecules mol^-1^); and 10^9^ is the factor that converts grams to nanogram. The efficiencies (E%) and correlation coefficients (r^2^) of the standard curves used for quantification of DEGs were 102.17 ± 7.14% and 0.9930 ± 0.0077, respectively.

### Cytotoxic effects of biofilm-derived bacteria

CHSE-214 embryo cells (ATCC 1681) from Chinook salmon were grown at 18°C on 24-well microplates (flat-bottomed; SPL Life Sciences Co., Ltd.) at 1 × 10^5^ cells per well in the Leibovitz’s L-15 medium (HyClone Laboratories Inc., Logan, Utah, USA) supplemented with 10% fetal bovine serum (Gibco, Invitrogen Laboratories), 6 mM L-glutamine (Gibco), 15 mM HEPES pH 7.3 (Gibco), and 100 μg mL^-1^/100 IU mL^-1^ streptomycin/penicillin (Gibco). CHSE-214 cells were washed in phosphate-buffered saline (1X PBS, pH 7.0) and then refreshed with fresh antibiotic-free medium (2 mL per well) with 2% fetal bovine serum (Gibco) prior to bacterial infections. Preliminary experiments indicated that the supplemented Leibovitz’s L-15 medium alone was unable to support the growth of *P*. *salmonis*.

The CHSE-214 cell line was infected with biofilm-derived and planktonic bacteria of *P*. *salmonis* LF-89^T^ and CA5. The inocula were prepared by growing both *P*. *salmonis* strains in AUSTRAL-SRS medium under routine conditions until achieving a concentration of ~1 × 10^6^ CFU mL^-1^ (determined by standard plate counting). Afterward, 20 mL of each culture were added to sterile glass Petri dishes and statically incubated in quadruplicate per strain to induce biofilm formation at 18°C for 48 h. After incubation, bacterial supernatants were withdrawn from the Petri dishes, while biofilms formed in the bottom were washed (3X) with 10 mL of sterile AUSTRAL-SRS medium by gentle manual shaking of the plates for 5 min. Later, washed Petri dishes were scraped with cell scrappers to collect biofilm-derived bacteria from the bottom by resuspending in 5 mL of sterile AUSTRAL-SRS medium to obtain a single 5 mL biofilm inoculum per strain in each independent experiment. The biofilm inocula of *P*. *salmonis* LF-89^T^ and CA5 were adjusted to 3.10 ± 0.42 × 10^6^ CFU mL^-1^ (i.e., 0.77 ± 0.04 McFarland) and 6.17 ± 0.76 × 10^6^ CFU mL^-1^ (0.78 ± 0.05 McFarland), respectively. Later, bacterial supernatants that were previously removed from the Petri dishes were adjusted to 6.93 ± 0.40 × 10^6^ CFU mL^-1^ (i.e., 0.80 ± 0.01 McFarland) and 2.53 ± 0.41 × 10^6^ CFU mL^-1^ (i.e., 0.78 ± 0.04 McFarland) to be used as planktonic inocula of *P*. *salmonis* LF-89^T^ and CA5, respectively. These planktonic inocula were included in the cytotoxicity trials for comparison purposes.

CHSE-214 cells were infected with biofilm-derived or planktonic bacteria of *P*. *salmonis* LF-89^T^ and CA5 by adding 100 µL of each inoculum per well. Considering the inocula sizes, each infection was carried out using a multiplicity of infection between 3 and 7. Three infection experiments were independently conducted with each strain (grown under biofilm and planktonic conditions) using triplicate infections at 18°C over a 72 h period. Afterward, aliquots of 100 μL were taken from each well at six different hours post-infection (i.e., at 0, 6, 12, 24, 48, and 72 hpi) to measure the LDH-based cytotoxicity with the Takara Kit (Bio Inc., Otsu, Japan) by spectrophotometric reading at 500 nm (Tecan microplate reader, Infinite 200 PRO, Männedorf, Switzerland). The absorbance measurements were corrected with the background absorbance measured in low controls (non-infected CHSE-214 cells) by using the equation supplied by the manufacturer. Resulting measurements were expressed as a percentage of the LDH-based cytotoxicity measured in high controls that consisted of CHSE-214 cells incubated without bacteria with 1% Triton X-100.

### RNA sequencing and data processing

Next-generation sequencing libraries and bioinformatic analyses were performed at Genoma Mayor Spa starting from total RNA extracts from biofilm-derived and planktonic samples of *P*. *salmonis* strain LF-89^T^. Total RNA extracts were subjected to digestion with DNase I to avoid contamination with genomic DNA. Later, the concentration and integrity of the RNA extracts were determined using the Quant-iT™ RiboGreen RNA Assay Kit (Thermo Fisher Scientific) and a Bioanalyzer 2100 (Agilent Technologies, CA, USA), respectively. The RNA libraries were prepared with the TruSeq™ RNA sample preparation kit according to the manufacturer’s protocol (Illumina Inc., CA, USA). Briefly, 28S, 18S, 5.8S, and 5S rRNAs were depleted starting from 500 ng of total RNA from each sample using the Ribo-Zero rRNA Removal Kit (Illumina Inc.); the depletion was confirmed by Bioanalyzer profiling. The rRNA-depleted RNA extracts were fragmented with divalent cations at an elevated temperature. The first-strand cDNA synthesis produced (by reverse transcription) single-stranded DNA copies from the fragmented RNA. After second-strand cDNA synthesis, the double-stranded DNA was end repaired, and the 3’ ends were adenylated. Universal adapters were then ligated to the cDNA fragments, and the final sequencing library was generated by PCR. After validation of the library by using the DNA 1000 chip and a Bioanalyzer (Agilent Technologies), the samples were pooled together in equal concentrations and run on an Illumina HiSeq System for 150 cycles of paired-end sequencing. The RNA sequencing data reported herein were deposited in the Sequence Read Archive (SRA) database under the BioProject number PRJNA854674 for biofilm-derived (SAMN29445255 to SAMN29445260) and planktonic (SAMN29445261 to SAMN29445266) BioSamples.

Technical sequences removal (adapters) and filter quality of the next-generation sequencing reads were performed using Trim Galore (http://www.bioinformatics.babraham.ac.uk/projects/trim_galore/), discarding sequences with Phred quality scores (on average) < 18. The pre- and post-filtering quality assessment of reads was performed with the FastQC online tool (https://www.bioinformatics.babraham.ac.uk/projects/fastqc/). The high-quality reads were aligned to the reference genome *P*. *salmonis* LF-89^T^ (ATCC VR-1361; NZ_CP011849) using the bowtie2 aligner (version 2.3.4.3) (Langmead and Salzberg, 2012), which creates Sequence Alignment/Map output files. These files were then changed to Binary Alignment Map files using the “sort” tool from SAMtools (Li et al., 2009). The number of mapped reads to a single gene for each “.bam” file was computed using the HTSeq-count tool (Anders et al., 2015). By using the “bedtools getfasta” function in BEDTools (Quinlan, 2014), the genes of the reference genome were extracted to generate a transcript FASTA file with the coordinates of all genes in the genome. Starting from these genes, proteins were predicted with the Prokka tool (Seemann, 2014), and functional annotation at the GO level was performed using the EggNOG-Mapper method (Huerta-Cepas et al., 2017). Starting from the found GO terms, functional profiles of gene expression in biofilm-derived and planktonic samples were obtained with the Web Gene Ontology Annotation Plot (WEGO) tool (Ye et al., 2018), which assigns gene transcripts into the three broader GO functional categories (i.e., Cellular Component, Molecular Function, and Biological Process), as well as into subsets.

### Data analysis

DEGs were identified from RNA sequencing data using the DESeq2 package (Love et al., 2014) for the following four comparisons between two conditions: (1) 24-h versus 48-h-old biofilm conditions (BC 24h vs. 48h), (2) 24-h versus 48-h-old planktonic conditions (PC 24h vs. 48h), (3) biofilm versus planktonic conditions at 24 h (BC vs. PC 24h), and (4) biofilm versus planktonic conditions at 48 h (BC vs. PC 48h). Fold Changes (FC) of gene expression were determined using threshold values of 4-fold (i.e., log_2_ FC ≥ 2) and 0.25-fold (i.e., log_2_ FC ≤ -2) for up- and down-regulated genes, respectively. Ten DEGs identified from RNA sequencing data (**Supplementary Table S1**) were randomly selected to validate the respective dataset by absolute DEG quantification using RT-qPCR; absolute DEG counts were normalized by the concentration of the total RNA extracts. Normalized DEG counts were log_2_-transformed for computing FC ratios that were then compared with log_2_ FC values directly computed from RNA sequencing data (**Supplementary Figure S2**).

Beta regression analysis (Cribari-Neto and Zeileis, 2010) was performed to model beta-distributed dependent variables (e.g., rates and proportions) to describe and evaluate how the percentage of cytotoxicity induced on CHSE-214 cells varied through five-time intervals (0, 6, 12, 24, 48, and 72 hpi) considering two modes of growth (i.e., biofilm and planktonic states) and two infecting *P*. *salmonis* strains (LF-89^T^ and CA5). Similarly, beta regression analysis was implemented to model the fluorescence ratio of live-to-dead bacteria in biofilms through time (24-360 h) considering the factors strain (i.e., LF-89^T^ and CA5) and mode of growth (i.e., planktonic vs biofilm states). As for the percentage of cytotoxicity and fluorescence ratio, a completely orthogonal model was built accounting for the effect of each factor on each response variable. All beta regression analyses were run using the “betareg” package (Cribari-Neto and Zeileis, 2010), while the “emmeans” package (Lenth et al., 2018) was used to implement the posteriori Tukey’s test to identify significant differences between treatment combinations. Additionally, a generalized linear model was implemented by assuming a Gaussian distribution with an identity function to test how the fluorescence signal (in RFU) of live bacteria in biofilms and specific biofilm formation (SBF) index varied through time intervals (i.e., between 24-360 h [RFU] and 24-312 h [SBF index]) considering *P*. *salmonis* strains LF-89^T^ and CA5. Here, a Gaussian distribution was assumed by the Akaike information criterion using the ‘propagate’ package (Spiess, 2013). For all analyses, an orthogonal model was implemented considering two factors (i.e., *P*. *salmonis* strain and time). Later, significant differences between treatment combinations were evaluated by the Tukey’s HSD *post hoc* test by using the “emmeans” package. All analyses considered a critical value of 0.05. Finally, a lognormal distribution of four parameters was fitted across time-intervals (between 24-360 h) to predict the temporal trajectory of the fluorescence signal of live sessile bacteria of *P*. *salmonis* LF-89^T^ and CA5 (see equation in **Supplementary Figure S3**). All analyses were performed using R version 4.2.0 (R Core Team, 2013).

## Results

### Characterization of *P*. *salmonis* biofilms

**Figure 1:**
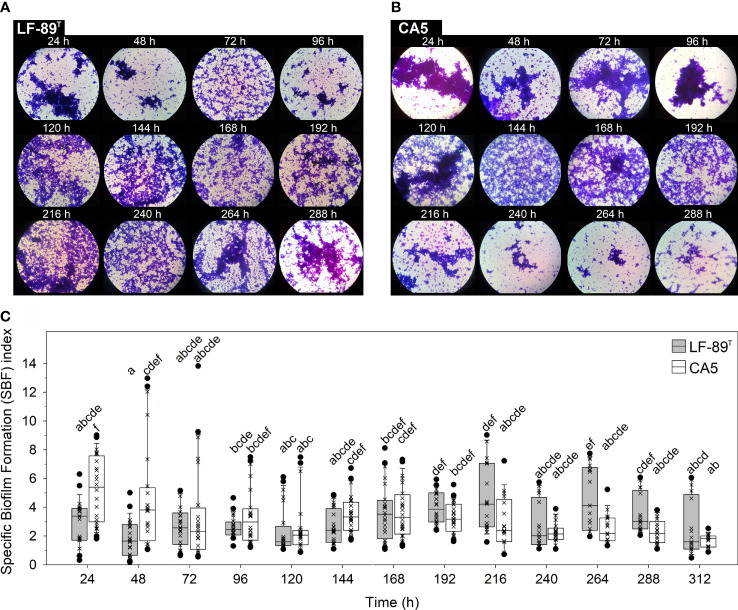
Crystal violet-stained biofilms and specific biofilm formation (SBF) index. The bulk fraction of the biofilm structure formed by *P*. *salmonis* **(A)** LF-89^T^ and **(B)** CA5 was visualized under 1,000X magnification. **(C)** Box plots show the medians, upper/lower quartiles (boxes), value ranges (vertical lines), and extreme values (circles) of SBF index data sets (×). Statistically significant differences between SBF index boxes (Tukey HSD test; P < 0.05) are indicated with different letters. Data were collected from three independent experiments.

The interaction between time and strain had a highly significant effect on the variability of the specific biofilm formation (SBF) index, yet the second factor alone did not have a significant effect (**Table 1**). Crystal violet staining revealed variations associated with changes in the bulk fraction of the biofilm structure (**Figure 1A**). These changes were coherent with those in the SBF index (**Figure 1**). While no statistically significant interstrain differences in the SBF index were detected at any time, excepting higher indexes for strain CA5 in the first 48 h (**Figure 1**), the two strains showed opposite general trends in variability of these indexes. In addition, there were some significant intra and interstrain differences in the SBF index between different incubation times (**Supplementary Table S2**). For instance, differences were detected between the first 24 h and subsequent sampling times (i.e., 72, 120, and 216-312 h) for *P*. *salmonis* CA5, as well as between *P*. *salmonis* CA5 at 24 h and *P*. *salmonis* LF-89^T^ at subsequent times (i.e., 48-144, 240, and 312 h) (**Figure 1** and **Supplementary Table S2**).

**Table 1:** Generalized linear model results for the effects of the “time” and “strain” factors (and their interaction) on different response variables.

Model term	df	F-ratio	p-value
**(A) SBF index**			
Time	12	5.903	0.000****
Strain	1	0.107	ns
Time × strain	12	6.490	0.000****
**(B) Fluorescence signal of live bacteria in biofilms**			
Time	14	90.143	0.000****
Strain	1	412.483	0.000****
Time × strain	14	10.735	0.000****
**(C) Fluorescence ratio of live-to-dead bacteria in biofilms**			
Time	14	69,359.000	0.000****
Strain	1	77,865.000	0.000***
Time × strain	14	9,098.000	0.000****

(A) specific biofilm formation (SBF) index, (B) fluorescence signal of live bacteria in biofilms, and (C) fluorescence ratio of live-to-dead bacteria in biofilms. The level of statistical significance is denoted by asterisks as follows: ‘****’P ≤ 0.0001; ‘***’P ≤ 0.001; ‘**’P ≤ 0.01; ‘*’P ≤ 0.05, and non-significant (ns = P > 0.05). Degrees of freedom (df).

**Figure 2:**
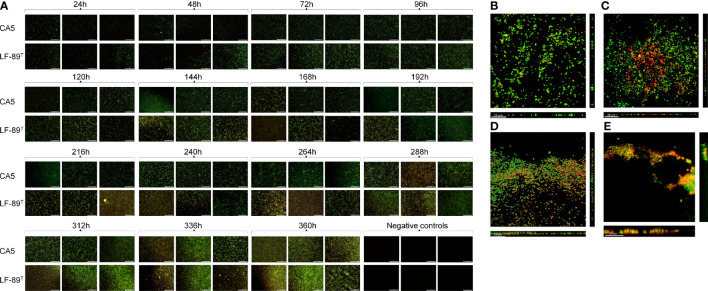
Automated imaging of biofilm development in 96‐well microplates and SCLM micrographs of 48-h-old biofilms. **(A)** Biofilms of *P*. *salmonis* LF-89^T^ and CA5 on the bottom of wells (including negative-control wells containing sterile AUSTRAL-SRS broth) were stained with the LIVE/DEAD bacterial viability kit at different times. For SCLM, 48-h-old biofilms of *P*. *salmonis* **(B, C)** LF-89^T^ and **(D, E)** CA5 were stained with the **(B, D)** LIVE/DEAD reagent or **(C, E)** fluorophore-conjugated WGA and DAPI stain. Bacteria stained with DAPI are depicted in orange only for visualization purposes. Automatically captured and SCLM images include a white scale bar in the lower‐right (100 µm) and lower‐left (15-20 µm) corners, respectively. All images are representative of three independent experiments.

The surface coverage of live and dead bacteria in biofilms tended to increase over time, but with a higher contribution of dead (or inactive) bacteria towards the end of the incubation period (**Figure 2**). The strain LF-89^T^ formed biofilms as very thin layers at 48 h (**Figure 2**), with an exopolysaccharide matrix revealed by wheat-germ-agglutinin lectin targeting *N*-acetyl sugar matrix components (**Figure 2**). Similarly, 48-h-old biofilms of *P*. *salmonis* CA5 were structured frequently as thin layers (**Figure 2**) supported by an exopolysaccharide matrix (**Figure 2**). In addition, *P*. *salmonis* LF-89^T^ biofilms and planktonic counterparts at 48 h shared an API ZYM profile composed of four enzymatic activities (i.e., esterase, esterase lipase, leucine arylamidase, and acid phosphatase) (**Supplementary Figures S4A, B**). Four enzymatic activities (i.e., alkaline phosphatase, leucine arylamidase, valine arylamidase, and acid phosphatase) also made up the API ZYM profile of *P*. *salmonis* CA5 biofilms and planktonic counterparts at 48 h (**Supplementary Figures S4C, D**), but in addition, 48-h-old planktonic bacteria of *P*. *salmonis* CA5 exhibited beta-glucosidase activity (**Supplementary Figure S4D**).

**Figure 3:**
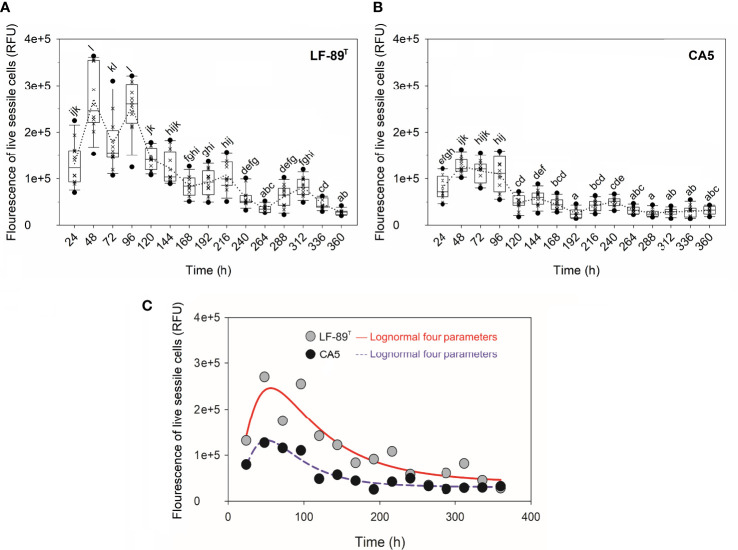
Fluorescence signal of live sessile bacteria and biofilm growth prediction. Fluorescence signal was measured in relative fluorescence units (RFU) in biofilms formed by *P*. *salmonis* **(A)** LF-89^T^ and **(B)** CA5 previously stained with the LIVE/DEAD reagent. Each box plot shows the median of fluorescence data sets (×), upper/lower quartiles (boxes), value ranges (vertical lines), extreme values (circles), and general patterns (dotted lines). Statistically significant differences between boxes (Tukey HSD test; P < 0.05) are denoted with different letters. **(C)** The biofilm mode of growth was predicted by fitting a four-parameter lognormal distribution to fluorescence data. The proportion of variance explained by this model accounted for ~88% on average. Data are representative of three independent experiments.

**Figure 4:**
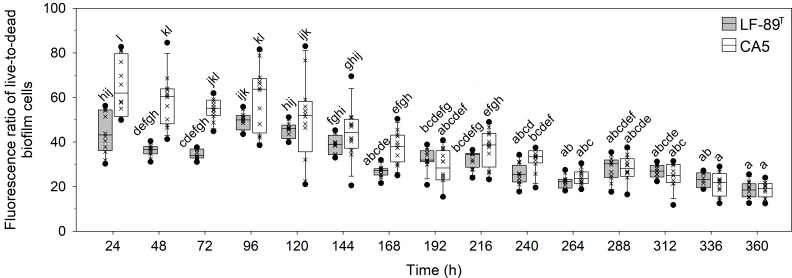
Temporal variations in fluorescence ratio of live-to-dead bacteria in biofilms formed by *P*. *salmonis* LF-89^T^ and CA5. Box plots show the medians of data sets (×), upper/lower quartiles (boxes), value ranges (vertical lines), and extreme values (circles). Statistically significant differences between boxes (Tukey HSD test; P < 0.05) are depicted with different letters. Data were obtained from three independent experiments.

The interaction between strain and time, as well as each factor separately, exerted a highly significant effect (P < 0.05) on the variables “fluorescence signal of live bacteria in biofilms” and “fluorescence ratio of live-to-dead bacteria in biofilms” (**Table 1**). The two *P*. *salmonis* strains emitted maximum levels of fluorescence from live bacteria during the first 48 h of biofilm formation; levels that, in general, were statistically significantly higher than subsequent times (**Figure 3A** and **Supplementary Table S3**). The two strains showed similar kinetic patterns of biofilm formation, and a four-parameter lognormal distribution provided the best fit to the fluorescence datasets (**Figures 3**). Maximum fluorescence peaks of live bacteria in biofilms formed by *P*. *salmonis* LF-89^T^ were significantly higher than those from live sessile *P*. *salmonis* CA5 during the first 96 h of biofilm development (**Figure 3A**). In addition, there was a general trend towards decreasing fluorescence ratios for live-to-dead bacteria in biofilms (i.e., viability ratios) towards the end of the incubation period (**Figure 4**). Significant differences in the viability ratio of sessile bacteria were detected between biofilms formed by *P*. *salmonis* CA5 and LF-89^T^ during the first 72 h, with higher viability ratios for biofilms formed by strain CA5 (**Figure 4**). Orthogonal comparison analysis further revealed additional significant intra and interstrain differences (**Supplementary Table S4**).

### Cytotoxic effects of biofilm-derived bacteria on the CHSE-214 cell line

**Figure 5:**
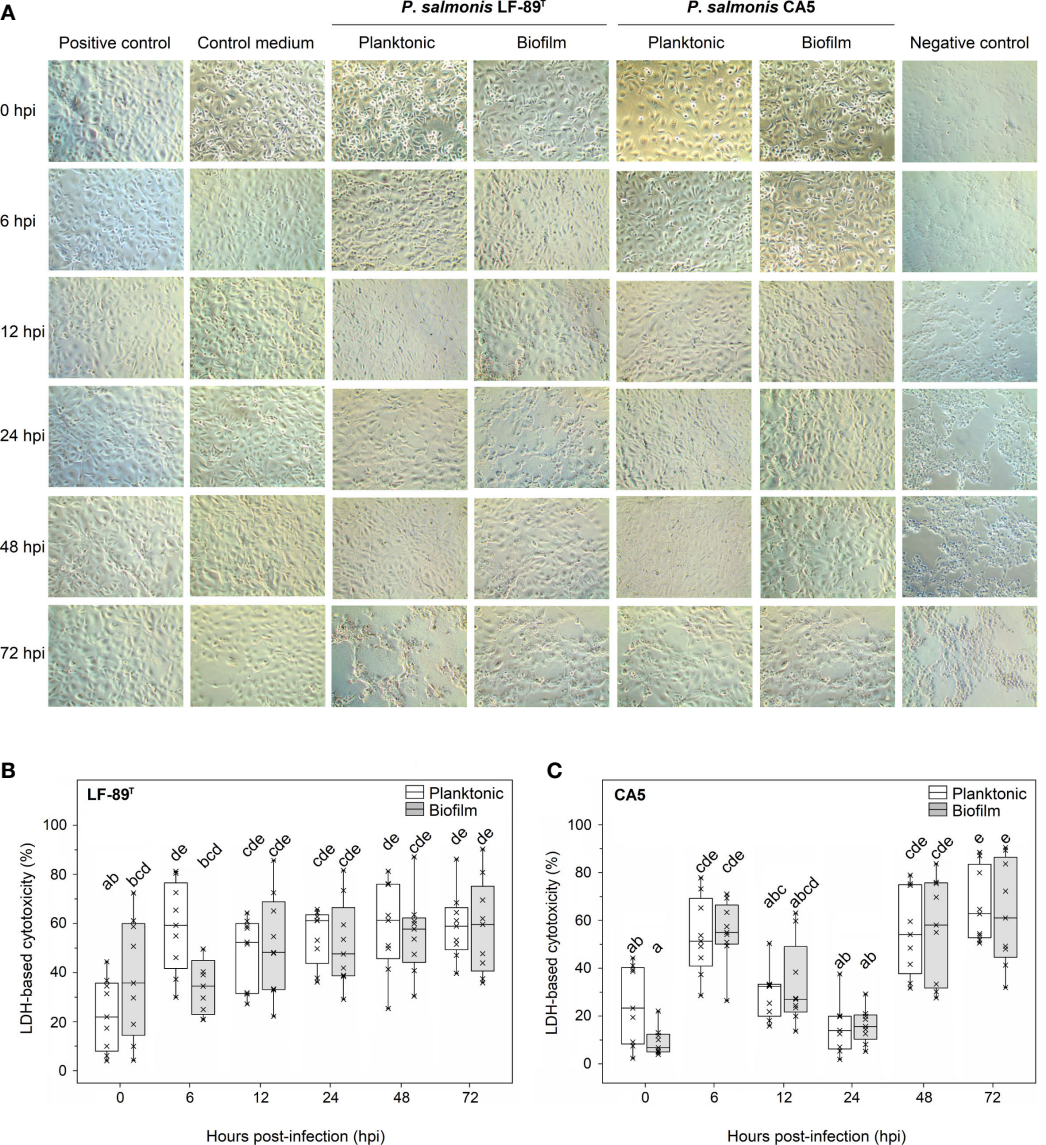
Cytotoxic effect on embryo cells from Chinook salmon (CHSE-214) induced by biofilm-derived and planktonic bacteria of *P*. *salmonis*. **(A)** Micrographs of CHSE-214 cells infected with 48-h-old biofilm-derived bacteria or planktonic counterparts. The cytotoxic effect was measured as the lactate dehydrogenase (LDH) release from CHSE-214 cells infected with *P*. *salmonis* **(B)** LF-89^T^ and **(C)** CA5. Box plots show the median of data sets (×), upper/lower quartiles (boxes), and range of percentages (vertical lines). Statistically significant differences between boxes (Tukey HSD test; P < 0.05) are depicted with different letters. Data were taken from three independent experiments.

A three-factor interaction between strain, time, and growth condition (i.e., biofilm versus planktonic states) significantly affected the variability of LDH-based cytotoxicity (**Table 2**). Remarkably, the factor “growth condition” alone was not a meaningful factor to explain cytotoxicity patterns (**Table 2**). There were no statistically significant differences in cytotoxicity due to *P*. *salmonis* LF-89^T^ or CA5 on Chinook salmon (*Oncorhynchus tshawytscha*) embryo (CHSE-214) cells when intrastrain comparisons between biofilm-derived and planktonic bacteria were performed at any given hpi (**Figure 5**). For biofilm-derived bacteria of *P*. *salmonis* LF-89^T^ (hereafter defined as biofilm-detached bacteria as single-cells and/or cell aggregates), no statistically significant differences in cytotoxicity between 0 hpi and later infection times were detected; however, the cytotoxic effect of planktonic bacteria of *P*. *salmonis* LF-89^T^ triggered a significant increment of the LDH-based measurements between 0 hpi and following infection times (**Figure 5**). Biofilm-derived and planktonic bacteria of *P*. *salmonis* CA5 induced an initial increase in cytotoxicity in CHSE-214 cells during the first 6 hpi. This was followed by a decrease in cytotoxicity between 12 and 24 hpi and, then, an important increase from 48 hpi onwards (**Figure 5**). Supplemental material is provided about significant intra and interstrain differences in cytotoxicity level induced on CHSE-214 cells by biofilm-derived bacteria of *P*. *salmonis* LF-89^T^ and CA5 (**Supplementary Table S5**).

**Table 2:** Beta regression analysis for testing the cytotoxic effect of *P. salmonis* on CHSE-214 cell line.

Model term	df	F-ratio	p-value
Time	5	37.851	0.000****
Strain	1	18.715	0.000****
Growth condition	1	0.411	ns
Time × strain	5	11.423	0.000****
Strain × growth condition	1	0.075	ns
Time × growth condition	5	0.911	ns
Time × strain × growth condition	5	2.685	0.019*

The cytotoxic effect of *P*. *salmonis* on CHSE-214 cells was analyzed depending on the infecting strain, the elapsed time after infection, and the bacterial inocula (i.e., biofilm-derived or planktonic bacteria) used in each infection. The level of statistical significance is denoted by asterisks as follows: ‘****’P ≤ 0.0001; ‘***’P ≤ 0.001; ‘**’P ≤ 0.01; ‘*’P ≤ 0.05, and non-significant (ns = P > 0.05). Degrees of freedom (df).

### Differential gene expression analysis in *P*. *salmonis* strain LF-89^T^

A total of 2,310 genes were expressed in the biofilm and/or planktonic modes of growth at 24 h and/or 48 h of incubation; indeed, the elapsed time to achieve mature biofilms in *P*. *salmonis* strain LF-89^T^ was 48 h. A small fraction of all expressed genes was exclusively transcribed in 24-h and 48-h-old biofilms (i.e., n = 35 and 78 genes, respectively). This fraction included eight genes encoding transposases (three of which were IS6 family transposases), in addition to three proteins (i.e., a LysR family transcriptional regulator; the type VI secretion system contractile sheath large subunit and a HigB family toxin). Of the transcribed genes (n = 2,310), only 157 were significantly differentially expressed genes (DEGs) (at Padj-values < 0.05) after four pairs of different conditions were compared, with different degrees of overlap in DEGs between comparisons (**Supplementary Figure S5**). Of the significant DEGs, 27 encoded virulence-related proteins, namely “stress response” (n = 12), “iron uptake” (n = 3), “endotoxins” (n = 2), and “other virulence-related genes” (n = 10) (**Supplementary Tables S6-S9**).

The total number of significant DEGs between 24-h and 48-h-old biofilms (**Supplementary Table S6**), or between biofilm and planktonic states at 48 h (**Supplementary Table S9**), was almost twice the total number of DEGs detected for the other two comparisons of conditions (**Supplementary Tables S7, S8**). DEGs that were upregulated between two specific conditions were found to be normally downregulated under another comparison of conditions. For example, approximately 67% of all upregulated DEGs in 24-h-old biofilms versus 48-h-old biofilms (**Supplementary Table S6**) were downregulated in 48-h-old biofilms versus planktonic counterparts (**Supplementary Table S9**). Mainly ribosomal proteins were involved, but DEGs encoding other proteins were detected.

**Figure 6:**
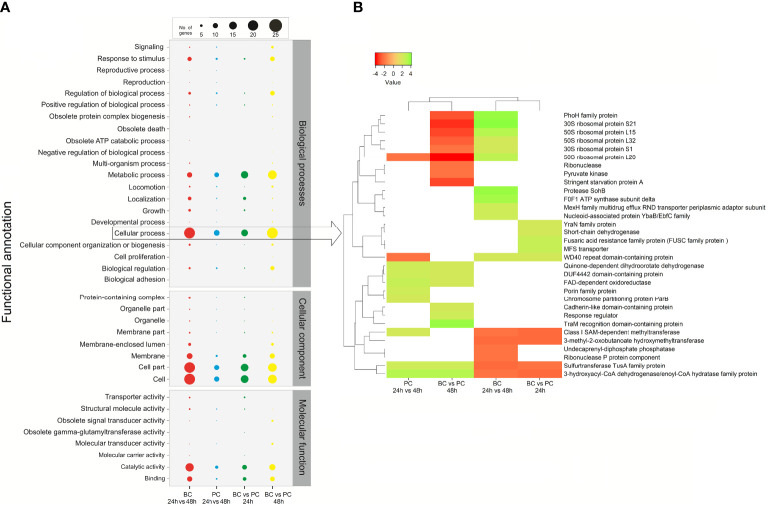
Comparative gene ontology (GO) functional classification of differentially expressed genes (DEGs) in *P*. *salmonis* LF-89^T^ obtained from four comparisons between different conditions. **(A)** The gene transcript dataset was analyzed with the WEGO tool set to second-level GO terms for the three major categories (Biological Process, Cellular Component, and Molecular Function). **(B)** The heatmap depicts significant DEGs between different conditions (i.e., modes of growth and/or incubation times) grouped at the ‘Cellular process’ GO term (see arrow).

Distribution analysis of the functional categories indicated that significant DEGs between 24-h and 48-h-old biofilms had a functional profile similar to profiling based on DEGs between biofilm and planktonic states at 48 h. Following in similarity was the functional profile obtained from DEGs between biofilm and planktonic states at 24 h, and finally by the functional profile from DEGs between planktonic bacteria at 24 and 48 h (**Figure 6**).

Significant DEGs in *P*. *salmonis* LF-89^T^ were grouped at the “cellular process” gene ontology (GO) term (**Figure 6**). In general, the results showed that some ribosomal proteins downregulated in 48-h-old biofilms (compared to planktonic bacteria at 48 h) were upregulated regarding the comparison 24-h versus 48-h-old biofilms (**Figure 6**). The same was true for the *phoH* gene, while the *sspA* gene (encoding the stringent starvation protein A) was downregulated in 48-h-old biofilms (compared to planktonic bacteria at the same timepoint) (**Figure 6**). Differential gene-expression analyses between 24-h and 48-h-old biofilms (BC 24 vs. 48 h in **Figure 6**) and/or between biofilms and planktonic bacteria at 24 h (BC vs. PC 24 h in **Figure 6**) indicated the upregulation of genes involved in protease activity, regulation of ATP synthesis, and efflux transporter activity, among others (**Figure 6**). Genes involved in cell adhesion (cadherin-like proteins), environmental sensing (response regulators), and RNA modifications or translation (*traM* gene) were upregulated in 48-h-old biofilms (compared to planktonic bacteria) (**Figure 6**). Additionally, a gene encoding an undecaprenyl-diphosphate phosphatase (involved in polysaccharide biosynthesis) was downregulated when 24-h and 48-h-old biofilms were compared; in other words, the respective gene was upregulated in mature biofilms (**Figure 6**).

## Discussion

The prevention of piscirickettsiosis and control of its etiological agent, *P*. *salmonis*, remain widely challenging in Chile (Rozas-Serri, 2022), the second largest salmon-producing nation worldwide behind Norway (Poblete et al., 2019). Despite recent research focused on biofilm formation by *P*. *salmonis* (Vidal et al., 2022), the significance of biofilm biology for piscirickettsiosis pathogenesis, as well as for many other aquatic animal diseases, has received little to no attention when compared with its notoriety in field of terrestrial animal diseases (Clutterbuck et al., 2007; Jacques et al., 2010). Many studies on bacterial pathogens have reached generalizations on biofilms and their relationship with virulence considering a limited number of traits (Gajdács et al., 2021) and genes (Wang et al., 2011). Likewise, thus far, studies on gene expression in *P*. *salmonis* biofilms have been conducted considering a limited number of genes (Marshall et al., 2012; Albornoz et al., 2017). The present study combined several methods, including whole-transcriptome sequencing, to examine and characterize the yet-cryptic nature of the biofilm development process by *P*. *salmonis*, in addition to exploring the relevance of this process as a significant virulence risk worth considering in disease pathogenesis.

Current literature on the biology of *P*. *salmonis* biofilms mentions several general traits that characterize these cellular accretions. Emphasis is particularly given to adhesion capacity on heterogeneous substrates (Albornoz et al., 2017; Larenas et al., 2019); the formation of cell aggregates (Marshall et al., 2012) or thin films (**Figure 2**), depending on culture conditions; secretion of an exopolysaccharide matrix (Marshall et al., 2012); fish-skin mucus tolerance (Levipan et al., 2020); and prolonged cell viability and survival in conditions of a nutrient-enriched medium and nutrient-poor seawater (Levipan et al., 2020). Live and dead bacteria plus the secreted exopolysaccharide matrix make up the bulk fraction of the biofilm structure (Azeredo et al., 2017), the temporal changes of which can be determined by analyzing variations in the SBF index. In the present study, *P*. *salmonis* CA5 showed a general temporal decline in the SBF index. Conversely, biofilms formed by *P*. *salmonis* LF-89^T^ showed an opposing pattern, with significant interstrain differences only detected in the first 48 h (**Figure 1**). The increasing trend in the SBF index exhibited by the type strain LF-89^T^ concorded with previously reported SBF-index patterns for other *P*. *salmonis* strains (Psal-103 and Psal-104) using the same culture medium and growth conditions (Levipan et al., 2020).

Biofilm-derived and planktonic bacteria of *P*. *salmonis* LF-89^T^ and CA5, at 48 h, showed a biochemical profile composed of four enzymatic activities that varied depending on the strain (**Supplementary Figures S4A–D**). Interestingly, beta-glucosidase activity was only detected in 48-h-old planktonic bacteria of *P*. *salmonis* CA5. The activity of beta-glucosidase can be inhibited by glucose as a reaction product (Salgado et al., 2018). Since the exopolysaccharide matrix mainly contains many types of carbohydrates (Jiao et al., 2010), the absence of beta-glucosidase activity in biofilms of *P*. *salmonis* CA5 could be related to the aforementioned enzymatic inhibition. The relevance of beta-glucosidase activity for the pathogenesis of piscirickettsiosis is unknown; nevertheless, this enzyme participates in carbohydrates metabolism (from bacteria to mammals). In general, the enzymatic profiles herein reported were consistent with previous reports on API-ZYM profiles for other Chilean and Canadian strains (Otterlei et al., 2016), as well as for patented isolates of *P*. *salmonis* (WO2014198913A2).

The prediction of biofilm development in *P*. *salmonis* was performed by fitting a four-parameter lognormal distribution to the fluorescence data from live sessile bacteria (**Figure 3**). The kinetics of biofilm formation followed a multi-step and strain-dependent process (**Table 1**). The results revealed a transient and undetectable lag phase in the first 24 h as a result of the temporal resolution achieved with the used sampling frequency. This transient phase then led to mature biofilms at 48 h with interstrain differences in maximum biofilm maturity levels. Over time, the fluorescence signal of live sessile bacteria declined and stabilized (**Figure 3**). Overall, the first 72 h of biofilm formation by *P*. *salmonis* CA5 were characterized by higher SBF indexes (**Figure 1**), a lower fluorescence signal of live bacteria (**Figure 3**), and higher cell viability ratios (**Figure 4**) when compared with biofilms formed by *P*. *salmonis* LF-89^T^. Taken together, these results suggest that interstrain differences in the SBF index could be a reflection of differences in exopolysaccharide productivity between strains (Poulin and Kuperman, 2021). In addition, the biofilm formation kinetics herein reported opposed the kinetics previously determined for other *P*. *salmonis* strains (Psal-103 and Psal-104) using the same sampling frequency, total sampling period, and methodology (Levipan et al., 2020). Indeed, biofilm formation depends on many physical and biological factors (Van Loosdrecht et al., 2002), including the bacterial species and/or strain. Consequently, some bacterial fish pathogens can generate different biofilm phenotypes in response to molecular mechanisms that, in turn, could even vary between isolates of the same species, as reported for the freshwater fish pathogen *F. psychrophilum* (Levipan and Avendaño-Herrera, 2017).

General patterns of cytotoxicity were mainly explained by the factors strain and time, as well as the factor interaction (**Table 2**). There were no significant differences in the cytotoxic response of CHSE-214 cells between infections with biofilm-derived and planktonic bacteria of *P*. *salmonis* LF-89^T^ or CA5 (**Figure 5**). This result aligns with infection trials conducted with SHK-1 cells and *P*. *salmonis* Psal-103 and Psal-104 using the same experimental setup in terms of sampling frequency, total incubation period (72 hpi), and methodology (Levipan et al., 2020). Another study reported differential cytotoxic responses in SHK-1 cells between infections with planktonic and biofilm-derived bacteria of different *P*. *salmonis* strains (including LF-89^T^), but from 10 dpi (Santibañez et al., 2020). Notably, freshly biofilm-detached bacteria can show higher expression levels of virulence-related genes compared with planktonic and biofilm bacteria; thus concurring with the respective cytotoxicity and representing a potential source for triggering acute infections (Chua et al., 2014). However, the phenotype of biofilm-detached bacteria can reverse to planktonic just a few hours after dispersion (Chua et al., 2014; Rumbaugh and Sauer, 2020). Considering the short phenotype half-life of freshly biofilm-detached bacterium, a short-term examination (e.g., ≤ 48 hpi) of biofilm-derived and planktonic-bacteria infections appears more appropriate when evaluating differential cytotoxic effects between both modes of growth.

A trait commonly reported in genomic and transcriptomic studies of *P*. *salmonis* is the presence and expression of genes encoding elements involved in genetic mobility and horizontal gene transfer (Nourdin-Galindo et al., 2017; Ortiz-Severín et al., 2019; Valenzuela-Miranda et al., 2020). In the present study, eight genes encoding transposases were transcribed exclusively in biofilm conditions, including IS6 family transposases (n = 3) involved in the rearrangement and spread of antimicrobial resistance (Harmer and Hall, 2019). Another three genes detected only in biofilms encoded a LysR-type transcriptional regulator (belonging to a family of bacterial regulators LTTRs) for diverse functions (e.g., cell division, biosynthesis, virulence, oxidative stress response, etc.) (Maddocks and Oyston, 2008; Gao et al., 2018); the type VI secretion system (T6SS) contractile sheath large subunit; and a HigB family toxin with unknown biological significance for *P*. *salmonis*. However, mutations on the toxin gene *higB* can lead to poorer pathogen adhesion, invasion, and propagation in fish host cells (Xie et al., 2021). The T6SS is implicated in bacterial relationships and host interactions (Vettiger and Basler, 2016) and has been detected in initial stages of *in vitro* intracellular infections by *P*. *salmonis* (Ortiz-Severín et al., 2020).

Based on the Virulence Factor Database for bacterial pathogens (http://www.mgc.ac.cn/VFs/main.htm), a prior study reported that the core-genome of *P*. *salmonis* is composed of 1,732 genes, with 135 falling into seven major categories or subcategories of virulence factors (i.e., “endotoxins”, “capsule and other surface component”, “secretion system”, “adherence, colonization and invasion factor”, “stress response”, “enzymes”, and “iron uptake”) (Nourdin-Galindo et al., 2017). Of the presently identified significant DEGs (n = 157), 27 encoded virulence-related proteins and accounted for 20% of virulence-related genes in the *P*. *salmonis* core-genome (Nourdin-Galindo et al., 2017). The specific category representation of 27 DEGs was as follows: “stress response” (n = 12); “iron uptake” (n = 3); “endotoxins” (n = 2); and “other virulence-related genes” (n = 10) (**Supplementary Tables S6-S9**).

A DEG associated with the “stress response” category and encoding an ATP-dependent protease subunit (ClpP) was upregulated in 24-h-old biofilms (Gene 857 in **Supplementary Table S6**). Aligning with this finding, *clpP* gene expression can increase once *P*. *salmonis* LF-89^T^ infects the SHK-1 cell line (Figueroa et al., 2021). In addition, an alkyl hydroperoxide reductase (AhpC), involved in potential protection against oxidative stress (Oh and Jeon, 2014), was encoded by a DEG upregulated in 24-h-old biofilms and downregulated in 48-h-old biofilms (Gene 938 in **Supplementary Tables S6, S9**). Other DEGs encoded several heat shock proteins. These proteins play key roles in bacterial protection against diverse stressors (Maleki et al., 2016), and some (i.e., GroES/GroEL and DnaK) have additionally been extensively used for vaccine design (Maisey et al., 2017). Heat shock proteins (Hsp) can be part of extracellularly secreted virulence factors, such as outer membrane vesicles (Tandberg et al., 2016; Oliver et al., 2017). DEGs encoding Hsp10 and Hsp60 (GroES and GroEL, respectively) were downregulated in 48-h-old biofilms (Genes 217 and 218 in **Supplementary Table S9**); however, the DEG encoding the Hsp70 (DnaK) was upregulated in 24-h-old biofilms but downregulated in 48-h-old biofilms (Gene 2909 in **Supplementary Tables S6, S9**). A similar up- and down-regulation pattern for biofilms (at 24 and 48 h, respectively) was observed for the gene encoding GrpE (Gene 2910 in **Supplementary Tables S8, S9**) and the DEG encoding the molecular chaperone HtpG (Gene 1254 in **Supplementary Tables S6, S9**). Additionally, some non-virulence DEGs encoding several ribosomal proteins, cell division proteins FtsA (Gene 3196) and/or FtsZ (Gene 3195), and elongation factors Ts (Gene 742) and G (Gene 2879) were downregulated in 48-h-old biofilms compared with 24-h-old biofilms (**Supplementary Table S6**) or as compared with planktonic bacteria at 48 h (**Supplementary Table S9**). These results suggest that changes in the gene expression of virulence-related genes were accompanied by a generalized reduction in translation machinery, a pattern that has previously been observed in biofilm formation by *F*. *psychrophilum* (Levipan et al., 2018). This pattern can vary depending on the conditions under which sessile growth occurs and/or the tested species. For example, under the influence of fish skin mucus, *F. columnare* biofilms upregulate genes associated with ribosome biogenesis and protein translation (Lange et al., 2018).

The “stress response” category further included DEGs encoding for a PhoH family protein, a universal stress protein (normally induced under various stressors) (Vollmer and Bark, 2018), the stringent starvation protein A (*sspA*) (Hansen and Jin, 2012), and a TraR/DksA family transcription regulator. The *phoH* gene is inducible in response to starvation signaling caused by phosphate limitation (Kim et al., 1993). In the present study, *phoH* was significantly upregulated in 24-h versus 48-h-old biofilms (Gene 2504 in **Supplementary Table S6**), suggesting a drop in phosphate concentrations during the earliest stage of biofilm formation. During the intracellular mode of growth of *P*. *salmonis*, and once nutrient deprivation starts, the bacterium can enter a survival mode known as a stringent response (Zúñiga et al., 2020; Ortiz-Severín et al., 2020). In our study, the following four points argue against activation of the stringent response in the first 48 h of biofilm formation: (1) the gene encoding the universal stress protein was downregulated in 24-h-old biofilms compared to the planktonic counterpart (Gene 1487 in **Supplementary Table S8**); (2) the *sspA* gene was downregulated in 48-h-old biofilms compared to the planktonic counterpart (Gene 431 in **Supplementary Table S9**); (3) genes involved in the stringent response, and that are normally differentially expressed during the intracellular growth of *P*. *salmonis* LF-89^T^ (Zúñiga et al., 2020), were not DEGs (or exclusively expressed genes) in biofilms; and (4) downregulation of a *dksA*/*traR* family gene in 48-h-old biofilms versus the planktonic counterpart (Gene 2832 in **Supplementary Table S9**). Bacterial control of the stringent response is regulated by ppGpp and DksA, but the distant homolog TraR would also exert a regulator effect when ppGpp is unavailable (Gourse et al., 2018).

Iron acquisition systems are among the most relevant pathogenic mechanisms described in *P*. *salmonis* LF-89^T^ (Almarza et al., 2016; Calquín et al., 2018); the bacterium has a set of at least 17 genes to cope with the iron-deprivation response of salmonids (Pulgar et al., 2015). In the present study, only three DEGs belonged to the “iron uptake” category. These encoded a diaminopimelate decarboxylase (Gene 1415) that was upregulated in 24-h-old biofilms (**Supplementary Tables S6, S8**) and downregulated in 48-h-old biofilms (**Supplementary Table S9**); a cysteine desulfurase-like protein upregulated in 24-h-old biofilms (Gene 356 in **Supplementary Table S6**); and a LbtU family siderophore porin downregulated in 48-h-old biofilms (Gene 148 in **Supplementary Table S9**). On the other hand, an acyl carrier protein and a dTDP-glucose 4,6-dehydratase – probably involved in O-antigen sugar biosynthesis (Nourdin-Galindo et al., 2017) – were encoded by DEGs belonging to the “endotoxins” category and were upregulated in 24-h-old biofilms (Genes 2430 and 105 in **Supplementary Tables S6, S8**, respectively).

The “other virulence-related genes” category included a DEG encoding a cadherin-like domain-containing protein that was significantly upregulated in 48-h-old biofilms (Gene 1226 in **Supplementary Table S9**); this gene may be involved in protein-protein interactions to form bacterial accretions (Fraiberg et al., 2010). Similarly, a DEG encoding a L,D-transpeptidase family protein was upregulated in 24-h-old biofilms (Gene 370 in **Supplementary Table S8**) and may be involved in biofilm formation through peptidoglycan cross-linking in sessile cells (Ye et al., 2016; Aliashkevich and Cava, 2022). This category also includes a DEG encoding a HU family DNA-binding protein that was upregulated in 24-h-old biofilms (Gene 860 in **Supplementary Table S6**) and may modulate several processes, including bacteria survival, the SOS response, and virulence genes expression, among other (Stojkova et al., 2019). Additionally detected were a DEG encoding a XRE family transcriptional regulator with importance in stress tolerance and virulence (Hu et al., 2019) that was upregulated in 24-h-old biofilms (Gene 280 in **Supplementary Tables S6, S8**) and a DEG encoding a MexH family multidrug efflux RND (resistance nodulation cell division) transporter adaptor subunit that was upregulated in 24-h-old biofilms (Gene 1792 in **Supplementary Table S6**). RND family transporters catalyze the efflux of many antimicrobial agents in Gram-negative bacteria (Nikaido and Takatsuka, 2009), and *P*. *salmonis* is not exempt (Sandoval et al., 2016). In addition, a gene encoding the protein phosphatase CheZ belonging to the chemotaxis operon *cheYZA* was upregulated in 48-h-old biofilms (Gene 1543 in **Supplementary Table S6**), which aligns with the reported relationship between biofilm formation and an overexpression of chemotaxis genes in *P*. *salmonis* (Albornoz et al., 2017). Importantly, despite *P*. *salmonis* having a set of genes encoding flagellar proteins, the bacterium lacks a flagellar structure (Carril et al., 2017).

In summary, *P*. *salmonis* strains LF-89^T^ and CA5 were avid biofilm formers under *in vitro* conditions and follow a traditional model of sessile growth in a multi-step, yet highly strain-dependent, process. No major differences existed in enzymatic profiles between biofilm-derived and planktonic bacteria for *P*. *salmonis* LF-89^T^ and CA5, and, likewise, significant differences were not found between these two modes of growth regarding the respective cytotoxicity levels induced by each strain on CHSE-214 cells. RNA sequencing analysis of *P*. *salmonis* LF-89^T^ during the early and mature stages of *in vitro* biofilm formation indicates resemblances in gene expression profiles to the *in vitro* intracellular growth of *P*. *salmonis* LF-89^T^ (Machuca and Martinez, 2016; Ortiz-Severín et al., 2020; Zúñiga et al., 2020). These resemblances are based on the gene expression of some proteases, Hsps, T6SS, chemotaxis proteins, endotoxin processing, protection against oxidative stress, translational machinery, and of genes involved in genetic mobility (Gene 3089 in **Supplementary Table S9**) and horizontal gene transfer (Genes 3376 and 3526 in **Supplementary Tables S6, S9**). Despite global differences in the functional profiles of gene expression between growth conditions for *P*. *salmonis* LF-89^T^ (**Figure 6**), just a few virulence-related genes were found to be upregulated in biofilms versus planktonic states at 24 and 48 h. The number of virulence-related genes exclusively expressed in the biofilm states was also small. Moreover, the bulk fraction of genes expected to encode virulence factors, according to the core-genome previously reported for *P*. *salmonis* (Nourdin-Galindo et al., 2017), did not show upregulation in biofilms. Importantly, the early and mature biofilm states were transcriptionally no more virulent than the planktonic counterparts, but, regardless of the strain, the two modes of growth induced important and very similar patterns of cytotoxicity on the CHSE-214 cell line (**Figure 5**). Considering these findings and the role of biofilms as a cell-persistence mechanism in *P*. *salmonis* (Marshall et al., 2012; Levipan et al., 2020), this study proposes *P*. *salmonis* biofilms be targets for epidemiological surveillance to determine if they can thrive in aquaculture settings and to understand how they contribute to piscirickettsiosis pathogenesis. Transcriptomic data generated here could be useful as a guide to advance these topics by enabling new avenues of research focused on individual genes (e.g., *via* single-gene mutant approaches) with some putative role in biofilm control.

## Data availability statement

The raw sequence data of this study can be found in the NCBI SRA database under the accession number PRJNA854674 (https://www.ncbi.nlm.nih.gov/bioproject/PRJNA854674/).

## Author contributions

HL and RA-H were responsible for the rationale and design of the study. RI maintained the bacterial strains and provided fresh axenic cultures. HL conducted the experiments with the support of HA-L for RT-qPCR assays. HL interpreted the data and wrote the manuscript. LO implemented the beta regression analysis, generalized linear model, and wrote the respective methodological section. RA-H participated in the critical analysis of the manuscript. All authors contributed to the article and approved the submitted version.

## Funding

This research was funded by Grants FONDAP-INCAR Center 15110027 and FONDECYT Iniciación 11200708 from the Agencia Nacional de Investigación y Desarrollo (ANID, Chile). The APC has been partially funded by Dirección General de Investigación (DGI) of UPLA.

## Acknowledgments

The Cytation-5 plate reader was supported by the CONICYT-FONDEQUIP EQM160131 instrumentation grant (Universidad de Playa Ancha). We are also grateful to Dr. C. Montero (BioTek Instruments, Inc.) for their technical specialist support.

## Conflict of interest

The authors declare that the research was conducted in the absence of any commercial or financial relationships that could be construed as a potential conflict of interest.

## Publisher’s note

All claims expressed in this article are solely those of the authors and do not necessarily represent those of their affiliated organizations, or those of the publisher, the editors and the reviewers. Any product that may be evaluated in this article, or claim that may be made by its manufacturer, is not guaranteed or endorsed by the publisher.

## References

[B1] Albornoz R. Valenzuela K. Pontigo J. P. Sánchez P. Ruiz P. Avendaño-Herrera R.. (2017). Identification of chemotaxis operon *cheYZA* and *cheA* gene expression under stressful conditions in *Piscirickettsia salmonis* . Microb. Pathog. 107, 436–441. doi: 10.1016/j.micpath.2017.04.030 28438636

[B2] Aliashkevich A. Cava F. (2022). LD-transpeptidases: The great unknown among the peptidoglycan cross-linkers. FEBS J. 289, 4718–4730. doi: 10.1111/febs.16066 34109739

[B3] Almarza O. Valderrama K. Ayala M. Segovia C. Santander J. (2016). A functional ferric uptake regulator (Fur) protein in the fish pathogen *Piscirickettsia salmonis* . Int. Microbiol. 19, 49–55. doi: 10.2436/20.1501.01.263 27762429

[B4] Anders S. Pyl P. T. Huber W. (2015). HTSeq─a Python framework to work with high-throughput sequencing data. Bioinformatics. 31, 166–169. doi: 10.1093/bioinformatics/btu638 25260700 PMC4287950

[B5] Avendaño-Herrera R. Mancilla M. Miranda C. D. (2022). Use of antimicrobials in Chilean salmon farming: Facts, myths and perspectives. Rev. Aquac. Early View 1-23. doi: 10.1111/raq.12702

[B6] Azeredo J. Azevedo N. F. Briandet R. Cerca N. Coenye T. Costa A. R.. (2017). Critical review on biofilm methods. Crit. Rev. Microbiol. 43, 313–351. doi: 10.1080/1040841X.2016.1208146 27868469

[B7] Bravo F. Sidhu J. P. S. Bernal P. Bustamante R. H. Condie S. Gorton B.. (2020). Hydrodynamic connectivity, water temperature, and salinity are major drivers of piscirickettsiosis prevalence and transmission among salmonid farms in Chile. Aquac. Environ. Interact. 12, 263–279. doi: 10.3354/aei00368

[B8] Calquín P. Ruiz P. Oliver C. Sánchez P. Haro R. Oliva H.. (2018). Physiological evidence that *Piscirickettsia salmonis* produces siderophores and uses iron from different sources. J. Fish. Dis. 41, 553–558. doi: 10.1111/jfd.12745 29193147

[B9] Carril G. P. Gómez F. A. Marshall S. H. (2017). Expression of flagellin and key regulatory flagellar genes in the non-motile bacterium *Piscirickettsia salmonis* . Dis. Aquat. Organ. 123, 29–43. doi: 10.3354/dao03079 28177291

[B10] Charlebois A. Jacques M. Archambault M. (2016). Comparative transcriptomic analysis of *Clostridium perfringens* biofilms and planktonic cells. Avian Pathol. 45, 593–601. doi: 10.1080/03079457.2016.1189512 27207477

[B11] Chua S. L. Liu Y. Yam J. K. H. Chen Y. Vejborg R. M. Tan B. G. C.. (2014). Dispersed cells represent a distinct stage in the transition from bacterial biofilm to planktonic lifestyles. Nat. Commun. 5, 4462. doi: 10.1038/ncomms5462 25042103

[B12] Clutterbuck A. L. Woods E. J. Knottenbelt D. C. Clegg P. D. Cochrane C. A. Percival S. L. (2007). Biofilms and their relevance to veterinary medicine. Vet. Microbiol. 121, 1–17. doi: 10.1016/j.vetmic.2006.12.029 17276630

[B13] Cribari-Neto F. Zeileis A. (2010). Beta regression in r. J. Stat. Software 34, 1–24. doi: 10.18637/jss.v034.i02

[B14] Díaz-Salazar C. Calero P. Espinosa-Portero R. Jiménez-Fernández A. Wirebrand L. Velasco-Domínguez M. G.. (2017). The stringent response promotes biofilm dispersal in *Pseudomonas putida* . Sci. Rep. 7, 18055. doi: 10.1038/s41598-017-18518-0 29273811 PMC5741744

[B15] Esteban M. A. Cerezuela R. (2015). “Fish mucosal immunity: Skin,” in Mucosal health in aquaculture. Eds. Beck B. H. Peatman E. (Elsevier Inc), 67–92.

[B16] Figueroa C. Veloso P. Espin L. Dixon B. Torrealba D. Elalfy I. S.. (2020). Host genetic variation explains reduced protection of commercial vaccines against *Piscirickettsia salmonis* in Atlantic salmon. Sci. Rep. 10, 18252. doi: 10.1038/s41598-020-70847-9 33106499 PMC7588420

[B17] Figueroa J. Villagrán D. Cartes C. Solis C. Nourdin-Galindo G. Haussmann D. (2021). Analysis of genes encoding for proteolytic enzymes and cytotoxic proteins as virulence factors of *Piscirickettsia salmonis* in SHK-1 cells. J. Fish. Dis. 44, 495–504. doi: 10.1111/jfd.13333 33455005

[B18] Flemming H.-C. Baveye P. Neu T. R. Stoodley P. Szewzyk U. Wingender J.. (2021). Who put the film in biofilm? the migration of a term from wastewater engineering to medicine and beyond. NPJ Biofilms Microbiomes. 7, 10. doi: 10.1038/s41522-020-00183-3 33504794 PMC7840925

[B19] Flemming H.-C. Wingender J. Szewzyk U. Steinberg P. Rice S. A. Kjelleberg S. (2016). Biofilms: An emergent form of bacterial life. Nat. Rev. Microbiol. 14, 563–575. doi: 10.1038/nrmicro.2016.94 27510863

[B20] Fraiberg M. Borovok I. Weiner R. M. Lamed R. (2010). Discovery and characterization of cadherin domains in *Saccharophagus degradans* 2-40. J. Bacteriol. 192, 1066–1074. doi: 10.1128/JB.01236-09 20023015 PMC2812970

[B21] Gaete-Carrasco A. Rosenfeld C. Gallardo A. (2019). Epidemiological analysis of the active surveillance programme for *Piscirickettsia salmonis* of the national fisheries and aquaculture service of Chile. Rev. Sci. Tech. 38, 823–849. doi: 10.20506/rst.38.3.3029 32286564

[B22] Gajdács M. Baráth Z. Kárpáti K. Szabó D. Usai D. Zanetti S.. (2021). No correlation between biofilm formation, virulence factors, and antibiotic resistance in *Pseudomonas aeruginosa*: Results from a laboratory-based *in vitro* study. Antibiotics. 10, 1134. doi: 10.3390/antibiotics10091134 34572716 PMC8471826

[B23] Gao X. Wang X. Mao Q. Xu R. Zhou X. Ma Y.. (2018). VqsA, a novel LysR-type transcriptional regulator, coordinates quorum sensing (QS) and is controlled by QS to regulate virulence in the pathogen *Vibrio alginolyticus* . Appl. Environ. Microbiol. 84, e00444–e00418. doi: 10.1128/AEM.00444-18 29625990 PMC5981076

[B24] Gómez F. A. Tobar J. A. Henríquez V. Sola M. Altamirano C. Marshall S. H. (2013). Evidence of the presence of a functional Dot/Icm type IV-b secretion system in the fish bacterial pathogen *Piscirickettsia salmonis* . PloS One 8, e54934. doi: 10.1371/journal.pone.0054934 23383004 PMC3557282

[B25] Gourse R. L. Chen A. Y. Gopalkrishnan S. Sanchez-Vazquez P. Myers A. Ross W. (2018). Transcriptional responses to ppGpp and DksA. Annu. Rev. Microbiol. 72, 163–184. doi: 10.1146/annurev-micro-090817-062444 30200857 PMC6586590

[B26] Høiby N. (2017). A short history of microbial biofilms and biofilm infections. APMIS. 125, 272–275. doi: 10.1111/apm.12686 28407426

[B27] Hansen A. M. Jin D. J. (2012). SspA up-regulates gene expression of the LEE pathogenicity island by decreasing h-NS levels in enterohemorrhagic *Escherichia coli* . BMC Microbiol. 12, 231. doi: 10.1186/1471-2180-12-231 23051860 PMC3539938

[B28] Happold J. Sadler R. Meyer A. Hillman A. Cowled B. Mackenzie C.. (2020). Effectiveness of vaccination for the control of salmonid rickettsial septicaemia in commercial salmon and trout farms in Chile. Aquaculture. 520, 734968. doi: 10.1016/j.aquaculture.2020.734968

[B29] Hariharan H. Amadi V. (2016). Shellfish as reservoirs of bacterial pathogens. J. Coast. Life Med. 4, 253–258. doi: 10.12980/jclm.4.2016J6-13

[B30] Harmer C. J. Hall R. M. (2019). An analysis of the IS6/IS26 family of insertion sequences: Is it a single family? Microb. Genom. 5, e000291. doi: 10.1099/mgen.0.000291 31486766 PMC6807381

[B31] Huerta-Cepas J. Forslund K. Coelho L. P. Szklarczyk D. Jensen L. J. Von Mering C.. (2017). Fast genome-wide functional annotation through orthology assignment by eggNOG-mapper. Mol. Biol. Evol. 34, 2115–2122. doi: 10.1093/molbev/msx148 28460117 PMC5850834

[B32] Hu Y. Hu Q. Wei R. Li R. Zhao D. Ge M.. (2019). The XRE family transcriptional regulator SrtR in *Streptococcus suis* is involved in oxidant tolerance and virulence. Front. Cell. Infect. Microbiol. 8. doi: 10.3389/fcimb.2018.00452 PMC6335249 30687648

[B33] Isla A. Haussmann D. Vera T. Kausel G. Figueroa J. (2014). Identification of the *clpB* and *bipA* genes and an evaluation of their expression as related to intracellular survival for the bacterial pathogen *Piscirickettsia salmonis* . Vet. Microbiol. 173, 390–394. doi: 10.1016/j.vetmic.2014.08.014 25205198

[B34] Jacques M. Aragon V. Tremblay Y. D. (2010). Biofilm formation in bacterial pathogens of veterinary importance. Anim. Health Res. Rev. 11, 97–121. doi: 10.1017/S1466252310000149 20969814

[B35] Jiao Y. Cody G. D. Harding A. K. Wilmes P. Schrenk M. Wheeler K. E.. (2010). Characterization of extracellular polymeric substances from acidophilic microbial biofilms. *Appl. Environ* . Microbiol. 76, 2916–2922. doi: 10.1128/AEM.02289-09 PMC2863431 20228116

[B36] Kim S. K. Makino K. Amemura M. Shinagawa H. Nakata A. (1993). Molecular analysis of the *phoH* gene, belonging to the phosphate regulon in *Escherichia coli* . J. Bacteriol. 175, 1316–1324. doi: 10.1128/jb.175.5.1316-1324.1993 8444794 PMC193217

[B37] Labra A. Arredondo-Zelada O. Flores-Herrera P. Marshall S. H. Gómez F. A. (2016). *In silico* identification and characterization of putative Dot/Icm secreted virulence effectors in the fish pathogen *Piscirickettsia salmonis* . Microb. Pathog. 92, 11–18. doi: 10.1016/j.micpath.2015.12.002 26706346

[B38] Lange M. D. Farmer B. D. Abernathy J. (2018). Catfish mucus alters the *Flavobacterium columnare* transcriptome. FEMS Microbiol. Lett. 365, fny244. doi: 10.1093/femsle/fny244 30285236

[B39] Langmead B. Salzberg S. (2012). Fast gapped-read alignment with bowtie 2. Nat. Methods 9, 357–359. doi: 10.1038/nmeth.1923 22388286 PMC3322381

[B40] Larenas J. J. Bartholomew J. Troncoso O. Fernández S. Ledezma H. Sandoval N.. (2003). Experimental vertical transmission of *Piscirickettsia salmonis* and *in vitro* study of attachment and mode of entrance into the fish ovum. Dis. Aquat. Organ. 56, 25–30. doi: 10.3354/dao056025 14524498

[B41] Larenas J. Perez M. Moraleda C. Godoy M. Larenas C. Acuña-Retamar M. (2019). *In vitro* adhesion and infectiveness of *Piscirickettsia salmonis* on mussel shells *Mytilus chilensis* . Bull. Eur. Assoc. Fish Pathol. 39, 114–121.

[B42] Lenth R. Singmann H. Love J. Buerkner P. Herve M. (2018) Emmeans: Estimated marginal means, aka least-squares means. Available at: https://CRAN.R-project.org/package=emmeans (Accessed March 2022).

[B43] Levipan H. A. Avendaño-Herrera R. (2017). Different phenotypes of mature biofilm in *Flavobacterium psychrophilum* share a potential for virulence that differs from planktonic state. Front. Cell. Infect. Microbiol. 7. doi: 10.3389/fcimb.2017.00076 PMC5350093 28361040

[B44] Levipan H. A. Avendaño-Herrera R. (2021). Assessing the impacts of skin mucus from *Salmo salar* and *Oncorhynchus mykiss* on the growth and *in vitro* infectivity of the fish pathogen *Piscirickettsia salmonis* . J. Fish. Dis. 44, 181–190. doi: 10.1111/jfd.13275 33006764

[B45] Levipan H. A. Irgang R. Yáñez A. Avendaño−Herrera R. (2020). Improved understanding of biofilm development by piscirickettsia salmonis reveals potential risks for the persistence and dissemination of piscirickettsiosis. Sci. Rep. 10, 12224. doi: 10.1038/s41598-020-68990-4 32699383 PMC7376020

[B46] Levipan H. A. Quezada J. Avendaño-Herrera R. (2018). Stress tolerance-related genetic traits of fish pathogen *Flavobacterium psychrophilum* in a mature biofilm. Front. Microbiol. 9. doi: 10.3389/fmicb.2018.00018 PMC5787105 29410654

[B47] Li H. Handsaker B. Wysoker A. Fennell T. Ruan J. Homer N. (2009). The sequence alignment/map format and SAMtools. Bioinformatics 25, 2078–2079. doi: 10.1093/bioinformatics/btp352 19505943 PMC2723002

[B48] Long A. Goodall A. Jones S. R. M. (2021). Development of a *Piscirickettsia salmonis* immersion challenge model to investigate the comparative susceptibility of three salmon species. J. Fish Dis. 44, 1–9. doi: 10.1111/jfd.13261 33067883 PMC7756497

[B49] Love M. I. Huber W. Anders S. (2014). Moderated estimation of fold change and dispersion for RNA-seq data with DESeq2. Genome Biol. 15, 550. doi: 10.1186/s13059-014-0550-8 25516281 PMC4302049

[B50] Machuca A. Martinez V. (2016). Transcriptome analysis of the intracellular facultative pathogen *Piscirickettsia salmonis*: Expression of putative groups of genes associated with virulence and iron metabolism. PloS One 11, e0168855. doi: 10.1371/journal.pone 28033422 PMC5199080

[B51] Mack W. N. Mack J. P. Ackerson A. O. (1975). Microbial film development in a trickling filter. Microb. Ecol. 2, 215–226. doi: 10.1007/BF02010441 24241336

[B52] Maddocks S. E. Oyston P. C. (2008). Structure and function of the LysR-type transcriptional regulator (LTTR) family proteins. Microbiology. 154, 3609–3623. doi: 10.1099/mic.0.2008/022772-0 19047729

[B53] Maisey K. Montero R. Christodoulides M. (2017). Vaccines for piscirickettsiosis (salmonid rickettsial septicaemia, SRS): The Chile perspective. Expert Rev. Vaccines 16, 215–228. doi: 10.1080/14760584.2017.1244483 27690686

[B54] Maleki F. Khosravi A. Nasser A. Taghinejad H. Azizian M. (2016). Bacterial heat shock protein activity. J. Clin. Diagn. Res. 10, BE01–BE03. doi: 10.7860/JCDR/2016/14568.7444 PMC4843247 27134861

[B55] Marshall S. H. Gómez F. A. Ramírez R. Nilo L. Henríquez V. (2012). Biofilm generation by *Piscirickettsia salmonis* under growth stress conditions: A putative *in vivo* survival/persistence strategy in marine environments. Res. Microbiol. 163, 557–566. doi: 10.1016/j.resmic.2012.08.002 22910282

[B56] Marshall S. H. Heath S. Henríquez V. Orrego C. (1998). Minimally invasive detection of *Piscirickettsia salmonis* in cultivated salmonids *via* the PCR. Appl. Environ. Microbiol. 64, 3066–3069. doi: 10.1128/AEM.64.8.3066-3069.1998 9687475 PMC106817

[B57] Nickel J. C. Ruseska I. Wright J. B. Costerton J. W. (1985). Tobramycin resistance of cells of *Pseudomonas aeruginosa* growing as a biofilm on urinary catheter material. Antimicrob. Agents Chemother. 27, 619–624. doi: 10.1128/AAC.27.4.619 3923925 PMC180108

[B58] Nikaido H. Takatsuka Y. (2009). Mechanisms of RND multidrug efflux pumps. Biochim. Biophys. Acta 1794, 769–781. doi: 10.1016/j.bbapap.2008.10.004 19026770 PMC2696896

[B59] Niu C. Gilbert E. S. (2004). Colorimetric method for identifying plant essential oil components that affect biofilm formation and structure. Appl. Environ. Microbiol. 70, 6951–6956. doi: 10.1128/AEM.70.12.6951-6956.2004 15574886 PMC535164

[B60] Nourdin-Galindo G. Sánchez P. Molina C. F. Espinoza-Rojas D. A. Oliver C. Ruiz P.. (2017). Comparative pan-genome analysis of *Piscirickettsia salmonis* reveals genomic divergences within genogroups. Front. Cell. Infect. Microbiol. 7. doi: 10.3389/fcimb.2017.00459 PMC5671498 29164068

[B61] Oh E. Jeon B. (2014). Role of alkyl hydroperoxide reductase (AhpC) in the biofilm formation of *Campylobacter jejuni* . PloS One 9, e87312. doi: 10.1371/journal.pone.0087312 24498070 PMC3909096

[B62] Oliver C. Hernández M. A. Tandberg J. I. Valenzuela K. N. Lagos L. X. Haro R. E.. (2017). The proteome of biologically active membrane vesicles from *Piscirickettsia salmonis* LF-89 type strain identifies plasmid-encoded putative toxins. Front. Cell. Infect. Microbiol. 7. doi: 10.3389/fcimb.2017.00420 PMC5625009 29034215

[B63] Ortiz-Severín J. Tandberg J. I. Winther-Larsen H. C. Chávez F. P. Cambiazo V. (2021). Comparative analysis of salmon cell lines and zebrafish primary cell cultures infection with the fish pathogen *Piscirickettsia salmonis* . Microorganisms. 9, 2516. doi: 10.3390/microorganisms9122516 34946119 PMC8706985

[B64] Ortiz-Severín J. Travisany D. Maass A. Cambiazo V. Chávez F. P. (2020). Global proteomic profiling of *Piscirickettsia salmonis* and salmon macrophage-like cells during intracellular infection. Microorganisms. 8, 1845. doi: 10.3390/microorganisms8121845 33255149 PMC7760863

[B65] Ortiz-Severín J. Travisany D. Maass A. Chávez F. P. Cambiazo V. (2019). *Piscirickettsia salmonis* cryptic plasmids: Source of mobile DNA and virulence factors. Pathogens. 8, 269. doi: 10.3390/pathogens8040269 31795181 PMC6963756

[B66] Otterlei A. Brevik Ø.J. Jensen D. Duesund H. Sommerset I. Frost P.. (2016). Phenotypic and genetic characterization of *Piscirickettsia salmonis* from Chilean and Canadian salmonids. BMC Vet. Res. 12, 55. doi: 10.1186/s12917-016-0681-0 26975395 PMC4791975

[B67] Penesyan A. Paulsen I. T. Kjelleberg S. Gillings M. R. (2021). Three faces of biofilms: A microbial lifestyle, a nascent multicellular organism, and an incubator for diversity. NPJ Biofilms Microbiomes. 7, 1–9. doi: 10.1038/s41522-021-00251-2 34759294 PMC8581019

[B68] Poblete E. G. Drakeford B. M. Ferreira F. H. Barraza M. G. Failler P. (2019). The impact of trade and markets on Chilean Atlantic salmon farming. Aquac. Int. 27, 1465–1483. doi: 10.1007/s10499-019-00400-7

[B69] Poulin M. B. Kuperman L. L. (2021). Regulation of biofilm exopolysaccharide production by cyclic di-guanosine monophosphate. Front. Microbiol. 12. doi: 10.3389/fmicb.2021.730980 PMC8461298 34566936

[B70] Pulgar R. Hödar C. Travisany D. Zuñiga A. Domínguez C. Maass A.. (2015). Transcriptional response of Atlantic salmon families to *Piscirickettsia salmonis* infection highlights the relevance of the iron-deprivation defence system. BMC Genom. 16, 495. doi: 10.1186/s12864-015-1716-9 PMC4490697 26141111

[B71] Quinlan A. R. (2014). BEDTools: The swiss-army tool for genome feature analysis. Curr. Protoc. Bioinf. 47, 11–12. doi: 10.1002/0471250953.bi1112s47 PMC4213956 25199790

[B72] R Core Team (2013). R: A language and environment for statistical computing (Vienna, Austria: R Foundation for Statistical Computing). Available at: https://www.R-project.org/.

[B73] Rodrigues S. Paillard C. Van Dillen S. Tahrioui A. Berjeaud J. M. Dufour A.. (2018). Relation between biofilm and virulence in *Vibrio tapetis*: A transcriptomic study. Pathogens. 7, 92. doi: 10.3390/pathogens7040092 30486310 PMC6313714

[B74] Rozas-Serri M. (2022). Why does *Piscirickettsia salmonis* break the immunological paradigm in farmed salmon? biological context to understand the relative control of piscirickettsiosis. Front. Immunol. 13. doi: 10.3389/fimmu.2022.856896 PMC8979166 35386699

[B75] Rumbaugh K. P. Sauer K. (2020). Biofilm dispersion. Nat. Rev. Microbiol. 18, 571–586. doi: 10.1038/s41579-020-0385-0 32533131 PMC8564779

[B76] Saavedra J. Hernandez N. Osses A. Castillo A. Cancino A. Grothusen H.. (2017). Prevalence, geographic distribution and phenotypic differences of *Piscirickettsia salmonis* EM-90-like isolates. J. Fish Dis. 40, 1055–1063. doi: 10.1111/jfd.12581 28075013

[B77] Salgado J. C. S. Meleiro L. P. Carli S. Ward R. J. (2018). Glucose tolerant and glucose stimulated β-glucosidases–a review. Bioresour. Technol. 267, 704–713. doi: 10.1016/j.biortech.2018.07.137 30093225

[B78] Sandoval R. Oliver C. Valdivia S. Valenzuela K. Haro R. E. Sánchez P.. (2016). Resistance-nodulation-division efflux pump *acrAB* is modulated by florfenicol and contributes to drug resistance in the fish pathogen *Piscirickettsia salmonis* . FEMS Microbiol. Lett. 363, fnw102. doi: 10.1093/femsle/fnw102 27190287

[B79] Santibañez N. Vega M. Pérez T. Yáñez A. González-Stegmaier R. Figueroa J.. (2020). Biofilm produced *in vitro* by *Piscirickettsia salmonis* generates differential cytotoxicity levels and expression patterns of immune genes in the Atlantic salmon cell line SHK-1. Microorganisms. 8, 1609. doi: 10.3390/microorganisms8101609 33092013 PMC7594049

[B80] Seemann T. (2014). Prokka: Rapid prokaryotic genome annotation. Bioinformatics. 30, 2068–2069. doi: 10.1093/bioinformatics/btu153 24642063

[B81] Smith P. A. Contreras J. R. Rojas M. E. Guajardo A. Díaz S. Carbonero A. (2015). Infectivity of *Piscirickettsia salmonis* in immersion-bath exposed rainbow trout *Oncorhynchus mykiss* (Walbaum) fry. J. Fish. Dis. 38, 765–770. doi: 10.1111/jfd.12288 25168060

[B82] Spiess A.-N. (2013) Propagate: Propagation of uncertainty. Available at: https://CRAN.R-project.org/package=propagate (Accessed March 2022).

[B83] Stojkova P. Spidlova P. Stulik J. (2019). Nucleoid-associated protein HU: A lilliputian in gene regulation of bacterial virulence. Front. Cell. Infect. Microbiol. 9. doi: 10.3389/fcimb.2019.00159 PMC6523023 31134164

[B84] Tamayo R. Patimalla B. Camilli A. (2010). Growth in a biofilm induces a hyperinfectious phenotype in *Vibrio cholerae* . Infect. Immun. 78, 3560–3569. doi: 10.1128/IAI.00048-10 20515927 PMC2916270

[B85] Tandberg J. I. Lagos L. X. Langlete P. Berger E. Rishovd A. L. Roos N.. (2016). Comparative analysis of membrane vesicles from three *Piscirickettsia salmonis* isolates reveals differences in vesicle characteristics. PloS One 11, e0165099. doi: 10.1371/journal.pone.0165099 27764198 PMC5072724

[B86] Valenzuela-Miranda D. Valenzuela-Muñoz V. Nuñez-Acuña G. Gallardo-Escárate C. (2020). Long-term serial culture of *Piscirickettsia salmonis* leads to a genomic and transcriptomic reorganization affecting bacterial virulence. Aquaculture. 529, 735634. doi: 10.1016/j.aquaculture.2020.735634

[B87] Van Loosdrecht M. C. M. Heijnen J. J. Eberl H. Kreft J. Picioreanu C. (2002). Mathematical modelling of biofilm structures. Antonie Van Leeuwenhoek. 81, 245–256. doi: 10.1023/A:1020527020464 12448723

[B88] Vettiger A. Basler M. (2016). Type VI secretion system substrates are transferred and reused among sister cells. Cell. 167, 99–110.e12. doi: 10.1016/j.cell.2016.08.023 27616061

[B89] Vidal J. M. Ruiz P. Carrasco C. Barros J. Sepúlveda D. Ruiz-Tagle N.. (2022). *Piscirickettsia salmonis* forms a biofilm on nylon surface using a CDC biofilm reactor. J. Fish Dis. 45, 1099–1107. doi: 10.1111/jfd.13632 35543448

[B90] Vollmer A. C. Bark S. J. (2018). “Twenty-five years of investigating the universal stress protein: function, structure, and applications,” in Advances in applied microbiology. Eds. Gadd G. M. Sariaslani S. (Elsevier Inc), 1–36. 10.1016/bs.aambs.2017.10.001 29680123

[B91] Wang Y. Zhang W. Wu Z. Lu C. (2011). Reduced virulence is an important characteristic of biofilm infection of *Streptococcus suis* . FEMS Microbiol. Lett. 316, 36–43. doi: 10.1111/j.1574-6968.2010.02189.x 21204925

[B92] Xie J. Zhao Q. Huang H. Fang Z. Hu Y. (2021). *Edwardsiella piscicida* HigB: A type II toxin that is essential to oxidative resistance, biofilm formation, serum survival, intracellular propagation, and host infection. Aquaculture. 535, 736382. doi: 10.1016/j.aquaculture.2021.736382

[B93] Yáñez A. J. Silva H. Valenzuela K. Pontigo J. P. Godoy M. Troncoso J.. (2013). Two novel blood-free solid media for the culture of the salmonid pathogen *Piscirickettsia salmonis* . J. Fish Dis. 36, 587–591. doi: 10.1111/jfd.12034 23173561

[B94] Yáñez A. Valenzuela K. Silva H. Retamales J. Romero A. Enriquez R.. (2012). Broth medium for the successful culture of the fish pathogen *Piscirickettsia salmonis* . Dis. Aquat. Organ. 97, 197–205. doi: 10.3354/dao02403 22422090

[B95] Ye Y. Jiao R. Gao J. Li H. Ling N. Wu Q.. (2016). Proteins involved in responses to biofilm and planktonic modes in *Cronobacter sakazakii* . LWT-Food. Sci. Technol. 65, 1093–1099. doi: 10.1016/j.lwt.2015.09.039G

[B96] Ye J. Zhang Y. Cui H. Liu J. Wu Y. Cheng Y.. (2018). WEGO 2.0: A web tool for analyzing and plotting GO annotations 2018 update. Nucleic Acids Res. 46, W71–W75. doi: 10.1093/nar/gky400 29788377 PMC6030983

[B97] Zhang W. Ding W. Li Y. X. Tam C. Bougouffa S. Wang R.. (2019). Marine biofilms constitute a bank of hidden microbial diversity and functional potential. Nat. Commun. 10, 517. doi: 10.1038/s41467-019-08463-z 30705275 PMC6355793

[B98] Zúñiga A. Aravena P. Pulgar R. Travisany D. Ortiz-Severín J. Chávez F. P.. (2020). Transcriptomic changes of *Piscirickettsia salmonis* during intracellular growth in a salmon macrophage-like cell line. Front. Cell. Infect. Microbiol. 9. doi: 10.3389/fcimb.2019.00426 PMC6964531 31998656

