# Supplementary material for: Collective behavior and virulence arsenal of the fish pathogen Piscirickettsia salmonis in the biofilm realm
Source: Front Cell Infect Microbiol. 2022 Dec 5;12:1067514. doi: 10.3389/fcimb.2022.1067514 (PMC9760808; doi:10.3389/fcimb.2022.1067514)
Supplement: Supplementary file 1 [file DataSheet_1.pdf]

Collective behavior and virulence arsenal of the fish pathogen *Piscirickettsia salmonis* in the biofilm realm

Héctor A. Levipan*[1, 2], Rute Irgang[3, 4], L. Felipe Opazo[5, 6], Henry Araya-León[3, 4], and Ruben Avendaño-Herrera*[3, 4, 7]

*** Correspondence to:**

Héctor A. Levipan, hector.levipan@upla.cl – hlevipan46@gmail.com

Ruben Avendaño-Herrera, ravendano@unab.cl – reavendano@yahoo.com

Number of supplementary figures: 5

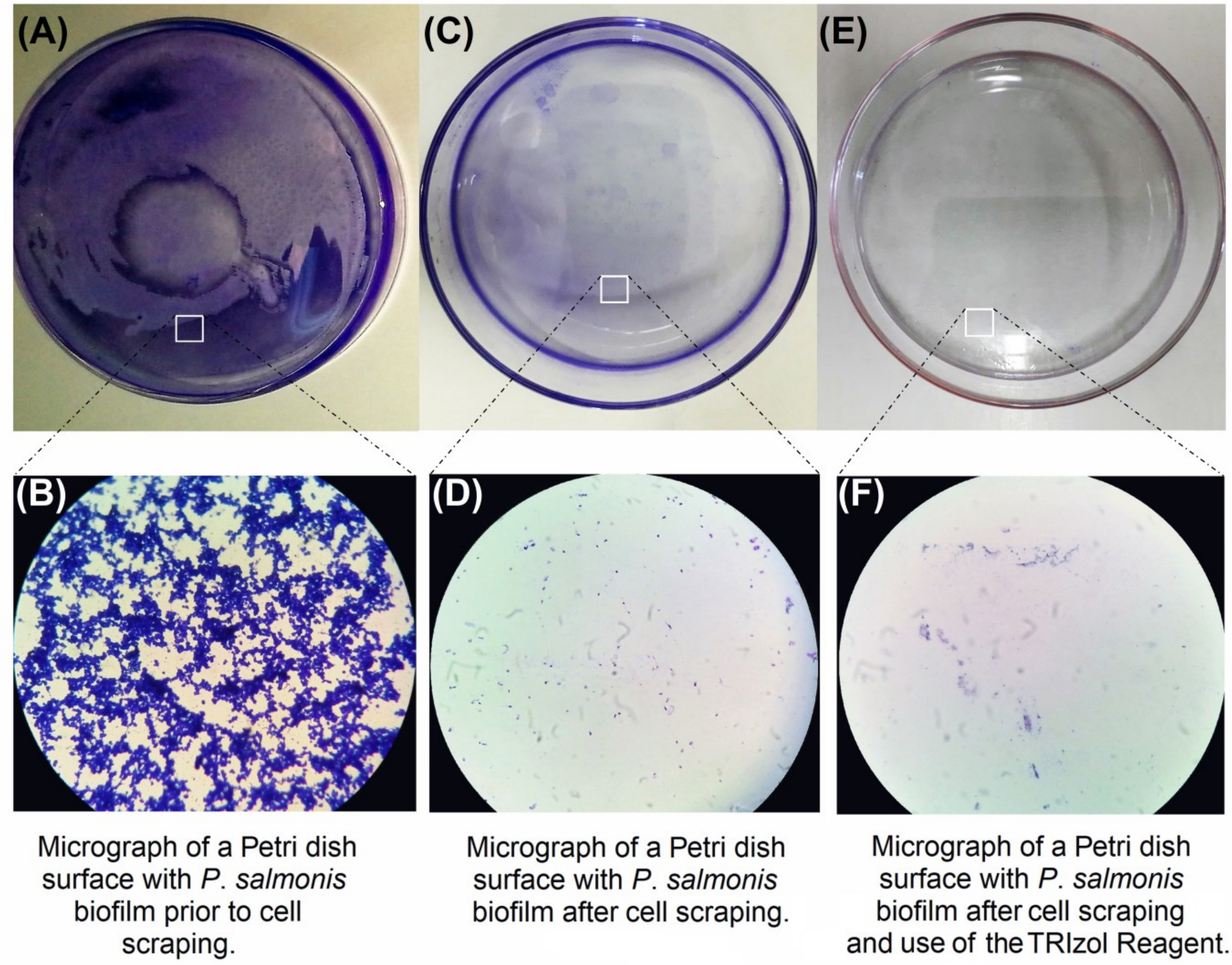

Supplementary Figure S1. Imaging of Petri dish surfaces with *Piscirickettsia salmonis* biofilms. (A) Non-scraped crystal violet (CV)-stained biofilms of *P. salmonis* LF-89^T on a glass Petri dish at 48 h (B) observed under 1000X magnification. (C) Scraped and CV-stained biofilms of *P. salmonis* LF-89^T on a glass Petri dish at 48 h (D) observed under 1000X magnification with almost complete biofilm detachment. (E) TRIzol-scraped CV-stained biofilms of *P. salmonis* LF-89^T on a glass Petri dish at 48 h (F) observed at 1000X magnification with complete biofilm detachment.

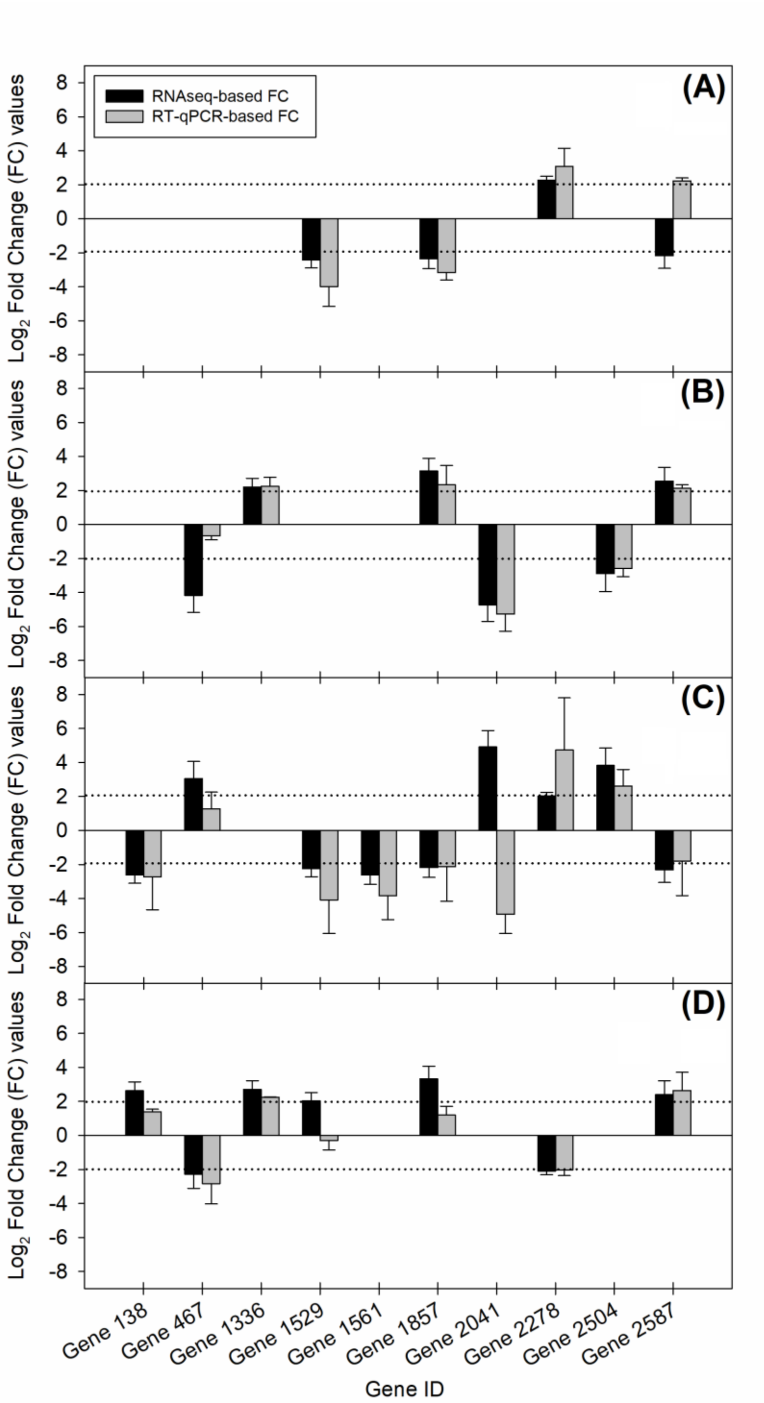

Supplementary Figure S2. Comparisons of $\log_2$ fold change (FC) data between values computed from RNA sequencing and RT-qPCR for ten randomly selected genes of *Piscirickettsia* salmonis LF-89^T. Log_2 FC threshold values ≥ 2 and ≤ −2 (dotted lines) indicate up- and down-regulated genes (i.e., differentially expressed genes (DEGs) at Padj-values < 0.05) between (A) biofilm and planktonic bacteria at 24 h, (B) biofilm and planktonic bacteria at 48 h, (C) 24-h and 48-h-old biofilms, and (D) 24-h and 48-h-old planktonic bacteria. Gene annotations can be found in Supplementary Tables S6-S9.

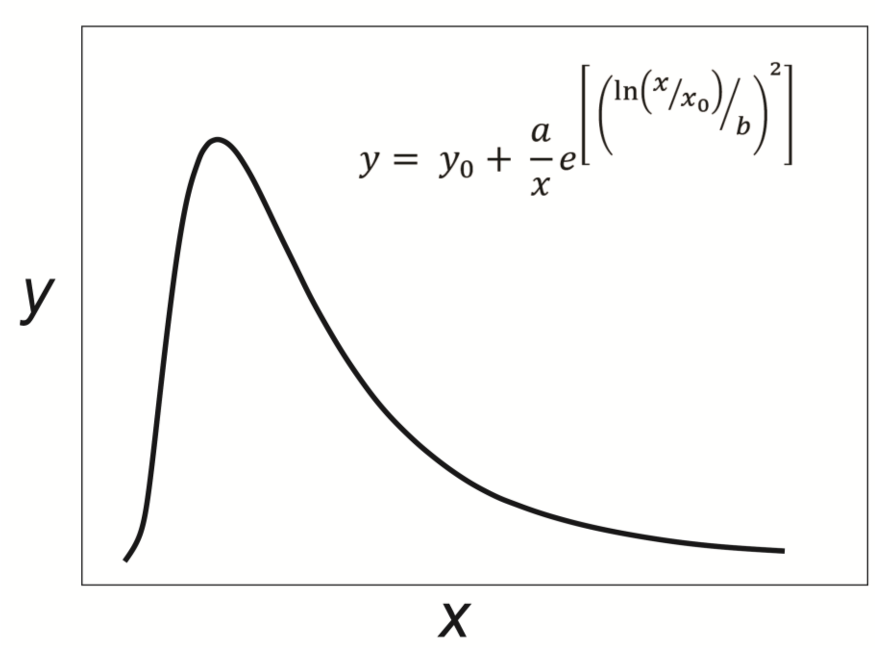

Supplementary Figure S3. Four parameter Lognormal peak function. The y-axis is the response variable. The x-axis is the predictor variable. y_0, is the initial value of y when x is zero; *a*, is the asymptotic maximum y value; x_0, is the position of the peak center; *b*, is the coefficient that controls the width of the bell (details can be found in Archontoulis & Miguez. 2015. Nonlinear regression models and applications in agricultural research. J. Agron. 107, 786-798; Di Marco & Bombi. 2001. Mathematical functions for the representation of chromatographic peaks. J. Chromatogr. A 931, 1-30).

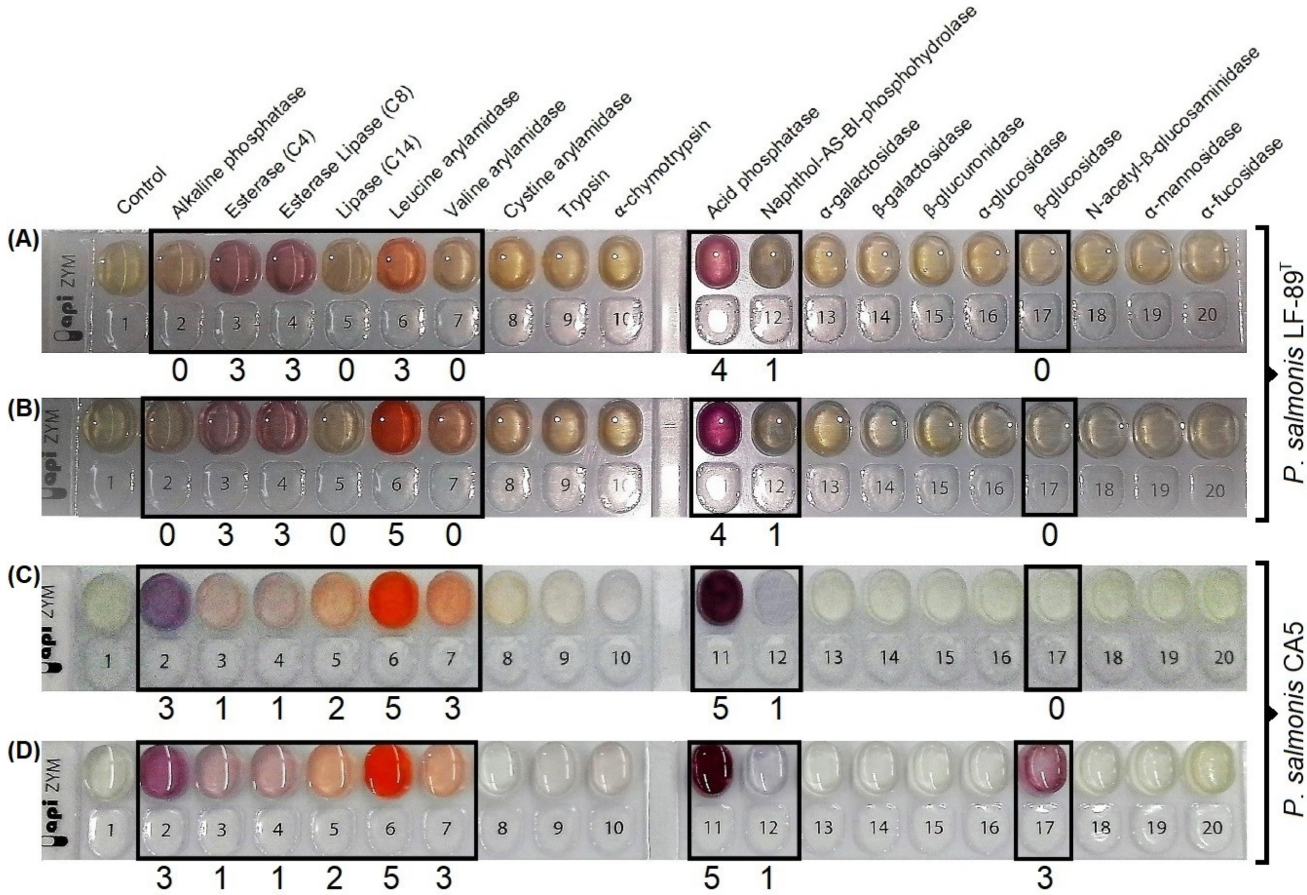

Supplementary Figure S4. API ZYM profiling of *Piscirickettsia salmonis*. (A, C) Biofilm-derived bacteria (at 48 h) of *P. salmonis* and (B, D) planktonic counterparts. Cupule number 1 of each strip is free of any substrate and represents a negative control. The numbers outside of the strips correspond to a color scale from 0 to 5, as provided by the manufacturer for the enzymatic activity level; only values ranging between 3 and 5 were considered true positive reactions. The results are representative of three independent experiments.

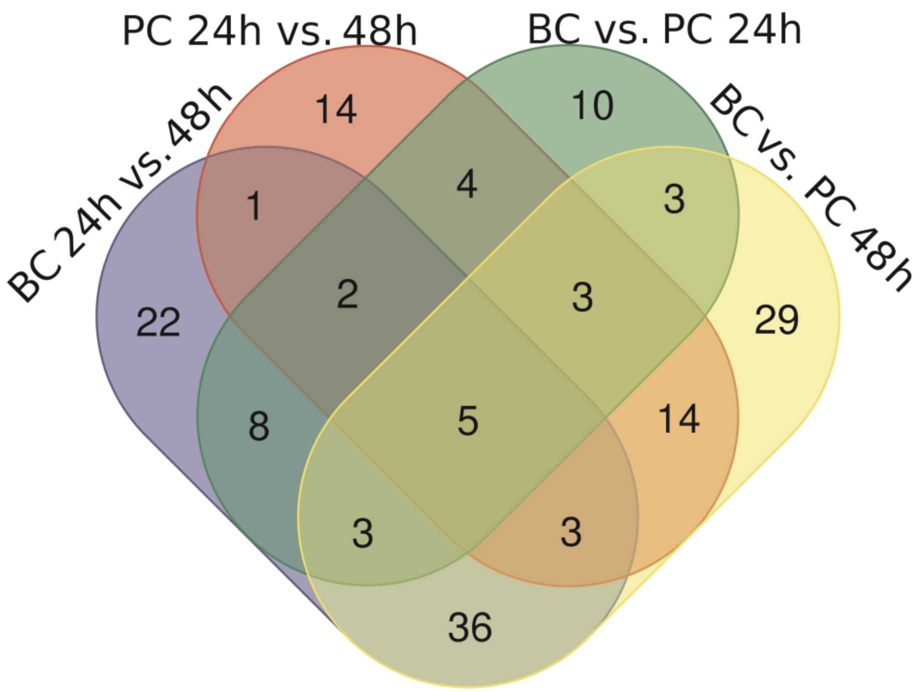

Supplementary Figure S5. Venn diagram depicting the degree of overlap in differentially expressed genes (DEGs) for four comparisons between two conditions. (1) Biofilm conditions (BC) at 24 h vs. 48 h, (2) planktonic conditions (PC) at 24 h vs. 48 h, (3) BC vs. PC at 24 h, and (4) BC vs. PC at 48 h.
