# Supplementary material for: Collective behavior and virulence arsenal of the fish pathogen Piscirickettsia salmonis in the biofilm realm
Source: Front Cell Infect Microbiol. 2022 Dec 5;12:1067514. doi: 10.3389/fcimb.2022.1067514 (PMC9760808; doi:10.3389/fcimb.2022.1067514)
Supplement: Supplementary file 2 [file DataSheet_2.pdf]

Collective behavior and virulence arsenal of the fish pathogen *Piscirickettsia salmonis* in the biofilm realm

Héctor A. Levipan*[1, 2], Rute Irgang[3, 4], L. Felipe Opazo[5, 6], Henry Araya-León[3, 4], and Ruben Avendaño-Herrera*[3, 4, 7]

*** Correspondence to:**

Héctor A. Levipan, hector.levipan@upla.cl – hlevipan46@gmail.com

Ruben Avendaño-Herrera, ravendano@unab.cl – reavendano@yahoo.com

Number of supplementary tables: 9

Supplementary Table S1. Quantitative PCR primers used in the present study to validate RNA sequencing data. Names, descriptions of target genes, amplicon lengths, primer sequences, and annealing temperatures are given.

Primer name	Description (Gene ID / annotation)	Amplicon length (bp)	Sequence (5' to 3')	Annealing temperature (ºC)
138F1<br>138R1	NZ_CP011849.2:131440-132291 / Hypothetical protein	156	AGTGGACCAAATGCCTGAGT<br>ACACTACAAGCCGGATCACC	56
467F1<br>467R1	NZ_CP011849.2:465946-466305 / 50S ribosomal protein L20	99	GCGCGCGCCCGTCATAAAA<br>GGCTTTGGTCACCGCTTGCT	59
1336F2<br>1336R2	NZ_CP011849.2:1348202-1349479 / FAD-dependent oxidoreductase	103	GCTGAGGCCCCAACGTTAAT<br>GCCCACGTACTGTCCGCAATG	59
1529F2<br>1529R2	NZ_CP011849.2:1542408-1543151 / Class I SAM-dependent methyltransferase	121	GCGAATGCCTTGCAGATTACCC<br>AACGCATGGGCAAGCACAAC	57
1561F2<br>1561R2	NZ_CP011849.2:1572798-1574027 / Hypothetical protein	141	GGCCCTACCTGTCTTGATTGT<br>GCTGCCGCAATCCCTAGTGT	58

1857F3<br>1857R3	NZ_CP011849.2:c1873696-1871330 / 3-hydroxyacyl-CoA dehydrogenase/enoyl-CoA hydratase family protein	84	GCGCTAAAATGCTCACCCTG<br>AGACGGGATAATGACGGCAA	56
2041F1<br>2041R1	NZ_CP011849.2:c2051892-2051221 / Hypothetical protein	163	CCACACCTTTGGTTGCTGAC<br>AAGAAAACCACAATAAAACGCGG	56
2278F2<br>2278R2	NZ_CP011849.2:2279991-2281022 / WD40 repeat domain-containing protein	144	AATCAACTAAGCACACCTTG<br>TGCTGTGAATAACTCTCGCTGA	55
2504F3<br>2504R3	NZ_CP011849.2:c2499753-2498761 / PhoH family protein	180	AGCCTCAGCCGATTCTTTCT<br>AGTCGGGGCAATGTCTTTCA	57
2587F3<br>2587R3	NZ_CP011849.2:c2580868-2580626 / Sulfurtransferase TusA family protein	102	CTTGGGTCTGTTGCCATCACT<br>TGTGGTTGGTTTACGTTGTCC	57

Supplementary Table S2. Tukey's multiple comparison test (HSD) for specific biofilm formation (SBF) indexes between two contrasted conditions. Only statistically significant condition comparisons are shown: '****'P ≤ 0.0001; '***'P ≤ 0.001; '**'P ≤ 0.01; and '*'P ≤ 0.05. Standard error (SE).

Contrast	Estimate	SE	z-ratio	p-value
24h, CA5 – 72h, CA5	0.717342713	0.165484869	4.334793359	0.004**
24h, CA5 – 120h, CA5	0.852194103	0.165484869	5.149679885	0.000****
24h, CA5 – 216h, CA5	0.681563713	0.181723361	3.750556388	0.038*
24h, CA5 – 240h, CA5	0.820757837	0.181723361	4.516523527	0.001**
24h, CA5 – 264h, CA5	0.698671823	0.181723361	3.844700086	0.027*
24h, CA5 – 288h, CA5	0.790883427	0.181723361	4.352128541	0.003**
24h, CA5 – 312h, CA5	1.085296847	0.181723361	5.972247264	0.000****
24h, CA5 – 24h, LF-89^T	0.701014778	0.165484869	4.236126125	0.006**
24h, CA5 – 48h, LF-89^T	1.274984871	0.165484869	7.704540458	0.000****
24h, CA5 – 72h, LF-89^T	0.750342368	0.165484869	4.534205281	0.001**
24h, CA5 – 96h, LF-89^T	0.647391231	0.165484869	3.912087151	0.021*
24h, CA5 – 120h, LF-89^T	0.903740827	0.165484869	5.461168934	0.000****
24h, CA5 – 144h, LF-89^T	0.692270714	0.165484869	4.183287067	0.007**
24h, CA5 – 240h, LF-89^T	0.71447098	0.181723361	3.931640794	0.020*
24h, CA5 – 312h, LF-89^T	0.8830418	0.181723361	4.859264068	0.000***
48h, CA5 – 312h, CA5	0.775597024	0.181723361	4.268009453	0.005**

48h, CA5 – 48h, LF-89^T	0.965285047	0.165484869	5.833071334	0.000****
96h, CA5 – 48h, LF-89^T	0.763551418	0.165484869	4.614025566	0.001**
120h, CA5 – 192h, LF-89^T	-0.634150247	0.165484869	-3.832073894	0.029*
120h, CA5 – 216h, LF-89^T	-0.689901495	0.181723361	-3.796438116	0.033*
120h, CA5 – 264h, LF-89^T	-0.709227784	0.181723361	-3.902788169	0.022*
144h, CA5 – 312h, CA5	0.713923947	0.181723361	3.928630543	0.020*
144h, CA5 – 48h, LF-89^T	0.903611971	0.165484869	5.460390275	0.000****
168h, CA5 – 312h, CA5	0.702019398	0.181723361	3.863121356	0.026*
168h, CA5 – 48h, LF-89^T	0.891707421	0.165484869	5.388452885	0.000****
192h, CA5 – 48h, LF-89^T	0.848296124	0.165484869	5.126124989	0.000****
312h, CA5 – 192h, LF-89^T	-0.867252992	0.181723361	-4.772380314	0.000***
312h, CA5 – 216h, LF-89^T	-0.923004239	0.196625324	-4.6942287	0.000***
312h, CA5 – 264h, LF-89^T	-0.942330528	0.196625324	-4.792518629	0.000***
312h, CA5 – 288h, LF-89^T	-0.739618878	0.196625324	-3.761564697	0.037*
48h, LF-89^T – 96h, LF-89^T	-0.62759364	0.165484869	-3.792453307	0.033*
48h, LF-89^T – 168h, LF-89^T	-0.852298611	0.165484869	-5.150311411	0.000****
48h, LF-89^T – 192h, LF-89^T	-1.056941015	0.165484869	-6.386934466	0.000****
48h, LF-89^T – 216h, LF-89^T	-1.112692263	0.181723361	-6.123000673	0.000****
48h, LF-89^T – 264h, LF-89^T	-1.132018552	0.181723361	-6.229350726	0.000****
48h, LF-89^T – 288h, LF-89^T	-0.929306901	0.181723361	-5.1138549	0.000****

120h, LF-89^T – 192h, LF-89^T	-0.685696972	0.165484869	-4.143562943	0.008**
120h, LF-89^T – 216h, LF-89^T	-0.74144822	0.181723361	-4.080093031	0.011*
120h, LF-89^T – 264h, LF-89^T	-0.760774509	0.181723361	-4.186443084	0.007**
264h, LF-89^T – 312h, LF-89^T	0.740075481	0.196625324	3.763886898	0.037*

Supplementary Table S3. Tukey's multiple comparison test (HSD) for fluorescence data of live sessile cells between two contrasted conditions. Only statistically significant condition comparisons are shown: '****'P ≤ 0.0001; '***'P ≤ 0.001; '**'P ≤ 0.01; and '*'P ≤ 0.05. Standard error (SE).

**Contrast**	**Estimate**	**SE**	**z-ratio**	**p-value**
24h, CA5 – 48h, CA5	-0.505359881	0.122342996	-4.130680938	0.012*
24h, CA5 – 120h, CA5	0.496513568	0.122342996	4.058373465	0.016*
24h, CA5 – 168h, CA5	0.569103249	0.122342996	4.651702735	0.001**
24h, CA5 – 192h, CA5	1.153695121	0.122342996	9.430005478	0.274****
24h, CA5 – 216h, CA5	0.620161842	0.122342996	5.069042468	0.000***
24h, CA5 – 264h, CA5	0.866978154	0.122342996	7.086455157	0.000****
24h, CA5 – 288h, CA5	1.09876851	0.122342996	8.981049561	0.000****
24h, CA5 – 312h, CA5	0.992903972	0.122342996	8.115740214	0.000****
24h, CA5 – 336h, CA5	1.023420527	0.122342996	8.365174644	0.000****
24h, CA5 – 360h, CA5	0.881162756	0.122342996	7.202396427	0.000****
24h, CA5 – 24h, LF-89^T	-0.493314223	0.122342996	-4.032222846	0.017*
24h, CA5 – 48h, LF-89^T	-1.234312498	0.122342996	-10.08895106	0.000****
24h, CA5 – 72h, LF-89^T	-0.791807517	0.122342996	-6.472029815	0.000****
24h, CA5 – 96h, LF-89^T	-1.182675512	0.122342996	-9.666883694	0.000****
24h, CA5 – 120h, LF-89^T	-0.612133477	0.122342996	-5.003420689	0.000***
24h, CA5 – 264h, LF-89^T	0.787266498	0.122342996	6.434912701	0.000****

24h, CA5 – 336h, LF-89^T	0.533930732	0.122342996	4.364211679	0.004**
24h, CA5 – 360h, LF-89^T	1.012622206	0.122342996	8.276911964	0.000****
48h, CA5 – 120h, CA5	1.001873449	0.122342996	8.189054403	0.000****
48h, CA5 – 144h, CA5	0.829592523	0.122342996	6.780874682	0.000****
48h, CA5 – 168h, CA5	1.07446313	0.122342996	8.782383673	0.000****
48h, CA5 – 192h, CA5	1.659055002	0.122342996	13.56068642	0.000****
48h, CA5 – 216h, CA5	1.125521723	0.122342996	9.199723406	0.000****
48h, CA5 – 240h, CA5	0.946598988	0.122342996	7.737255252	0.000****
48h, CA5 – 264h, CA5	1.372338035	0.122342996	11.2171361	0.000****
48h, CA5 – 288h, CA5	1.604128391	0.122342996	13.1117305	0.000****
48h, CA5 – 312h, CA5	1.498263853	0.122342996	12.24642115	0.000****
48h, CA5 – 336h, CA5	1.528780408	0.122342996	12.49585558	0.000****
48h, CA5 – 360h, CA5	1.386522638	0.122342996	11.33307737	0.000****
48h, CA5 – 48h, LF-89^T	-0.728952617	0.122342996	-5.958270119	0.000****
48h, CA5 – 96h, LF-89^T	-0.677315631	0.122342996	-5.536202755	0.000****
48h, CA5 – 240h, LF-89^T	0.805959776	0.122342996	6.587706717	0.000****
48h, CA5 – 264h, LF-89^T	1.292626379	0.122342996	10.56559364	0.000****
48h, CA5 – 288h, LF-89^T	0.80176301	0.122342996	6.553403439	0.000****
48h, CA5 – 336h, LF-89^T	1.039290613	0.122342996	8.494892618	0.000****
48h, CA5 – 360h, LF-89^T	1.517982087	0.122342996	12.4075929	0.000****

72h, CA5 – 120h, CA5	0.896173135	0.122342996	7.325087377	0.000****
72h, CA5 – 144h, CA5	0.723892209	0.122342996	5.916907655	0.000****
72h, CA5 – 168h, CA5	0.968762815	0.122342996	7.918416647	0.000****
72h, CA5 – 192h, CA5	1.553354688	0.122342996	12.69671939	0.000****
72h, CA5 – 216h, CA5	1.019821409	0.122342996	8.33575638	0.000****
72h, CA5 – 240h, CA5	0.840898673	0.122342996	6.873288226	0.000****
72h, CA5 – 264h, CA5	1.266637721	0.122342996	10.35316907	0.000****
72h, CA5 – 288h, CA5	1.498428076	0.122342996	12.24776347	0.000****
72h, CA5 – 312h, CA5	1.392563538	0.122342996	11.38245413	0.000****
72h, CA5 – 336h, CA5	1.423080094	0.122342996	11.63188856	0.000****
72h, CA5 – 360h, CA5	1.280822323	0.122342996	10.46911034	0.000****
72h, CA5 – 48h, LF-89^T	-0.834652931	0.122342996	-6.822237145	0.000****
72h, CA5 – 96h, LF-89^T	-0.783015945	0.122342996	-6.400169782	0.000****
72h, CA5 – 240h, LF-89^T	0.700259461	0.122342996	5.72373969	0.000****
72h, CA5 – 264h, LF-89^T	1.186926065	0.122342996	9.701626613	0.000****
72h, CA5 – 288h, LF-89^T	0.696062696	0.122342996	5.689436413	0.000****
72h, CA5 – 336h, LF-89^T	0.933590298	0.122342996	7.630925591	0.000****
72h, CA5 – 360h, LF-89^T	1.412281773	0.122342996	11.54362588	0.000****
96h, CA5 – 120h, CA5	0.80562592	0.122342996	6.584977865	0.000****
96h, CA5 – 144h, CA5	0.633344994	0.122342996	5.176798143	0.000****

96h, CA5 – 168h, CA5	0.8782156	0.122342996	7.178307135	0.000****
96h, CA5 – 192h, CA5	1.462807473	0.122342996	11.95660988	0.000****
96h, CA5 – 216h, CA5	0.929274194	0.122342996	7.595646868	0.000****
96h, CA5 – 240h, CA5	0.750351458	0.122342996	6.133178714	0.000****
96h, CA5 – 264h, CA5	1.176090506	0.122342996	9.613059557	0.000****
96h, CA5 – 288h, CA5	1.407880861	0.122342996	11.50765396	0.000****
96h, CA5 – 312h, CA5	1.302016323	0.122342996	10.64234461	0.000****
96h, CA5 – 336h, CA5	1.332532879	0.122342996	10.89177904	0.000****
96h, CA5 – 360h, CA5	1.190275108	0.122342996	9.729000827	0.000****
96h, CA5 – 48h, LF-89^T	-0.925200146	0.122342996	-7.562346657	0.000****
96h, CA5 – 72h, LF-89^T	-0.482695165	0.122342996	-3.945425415	0.024*
96h, CA5 – 96h, LF-89^T	-0.87356316	0.122342996	-7.140279294	0.000****
96h, CA5 – 240h, LF-89^T	0.609712246	0.122342996	4.983630178	0.000***
96h, CA5 – 264h, LF-89^T	1.09637885	0.122342996	8.961517101	0.000****
96h, CA5 – 288h, LF-89^T	0.605515481	0.122342996	4.949326901	0.000***
96h, CA5 – 336h, LF-89^T	0.843043083	0.122342996	6.890816079	0.000****
96h, CA5 – 360h, LF-89^T	1.321734558	0.122342996	10.80351636	0.000****
120h, CA5 – 192h, CA5	0.657181553	0.122342996	5.371632013	0.000****
120h, CA5 – 288h, CA5	0.602254941	0.122342996	4.922676096	0.000***
120h, CA5 – 312h, CA5	0.496390404	0.122342996	4.05736675	0.016*

120h, CA5 – 336h, CA5	0.526906959	0.122342996	4.30680118	0.005**
120h, CA5 – 24h, LF-89^T	-0.989827791	0.122342996	-8.090596311	0.000****
120h, CA5 – 48h, LF-89^T	-1.730826066	0.122342996	-14.14732452	0.000****
120h, CA5 – 72h, LF-89^T	-1.288321085	0.122342996	-10.53040328	0.000****
120h, CA5 – 96h, LF-89^T	-1.67918908	0.122342996	-13.72525716	0.000****
120h, CA5 – 120h, LF-89^T	-1.108647045	0.122342996	-9.061794154	0.000****
120h, CA5 – 144h, LF-89^T	-0.937043967	0.122342996	-7.659154989	0.000****
120h, CA5 – 168h, LF-89^T	-0.555343887	0.122342996	-4.539237274	0.002**
120h, CA5 – 192h, LF-89^T	-0.630958704	0.122342996	-5.157293228	0.000***
120h, CA5 – 216h, LF-89^T	-0.801540402	0.122342996	-6.551583902	0.000****
120h, CA5 – 312h, LF-89^T	-0.543594771	0.122342996	-4.443203035	0.003**
120h, CA5 – 360h, LF-89^T	0.516108638	0.122342996	4.218538499	0.008**
144h, CA5 – 192h, CA5	0.829462479	0.122342996	6.779811735	0.000****
144h, CA5 – 264h, CA5	0.542745512	0.122342996	4.436261414	0.003**
144h, CA5 – 288h, CA5	0.774535867	0.122342996	6.330855818	0.000****
144h, CA5 – 312h, CA5	0.668671329	0.122342996	5.465546471	0.000****
144h, CA5 – 336h, CA5	0.699187885	0.122342996	5.714980901	0.000****
144h, CA5 – 360h, CA5	0.556930114	0.122342996	4.552202684	0.002**
144h, CA5 – 24h, LF-89^T	-0.817546865	0.122342996	-6.682416589	0.000****
144h, CA5 – 48h, LF-89^T	-1.55854514	0.122342996	-12.7391448	0.000****

144h, CA5 – 72h, LF-89^T	-1.116040159	0.122342996	-9.122223558	0.000****
144h, CA5 – 96h, LF-89^T	-1.506908154	0.122342996	-12.31707744	0.000****
144h, CA5 – 120h, LF-89^T	-0.936366119	0.122342996	-7.653614433	0.000****
144h, CA5 – 144h, LF-89^T	-0.764763041	0.122342996	-6.250975268	0.000****
144h, CA5 – 192h, LF-89^T	-0.458677778	0.122342996	-3.749113507	0.049*
144h, CA5 – 216h, LF-89^T	-0.629259476	0.122342996	-5.14340418	0.000***
144h, CA5 – 264h, LF-89^T	0.463033856	0.122342996	3.784718958	0.044*
144h, CA5 – 360h, LF-89^T	0.688389564	0.122342996	5.626718221	0.000****
168h, CA5 – 192h, CA5	0.584591873	0.122342996	4.778302743	0.000***
168h, CA5 – 288h, CA5	0.529665261	0.122342996	4.329346826	0.005**
168h, CA5 – 24h, LF-89^T	-1.062417472	0.122342996	-8.683925581	0.000****
168h, CA5 – 48h, LF-89^T	-1.803415746	0.122342996	-14.74065379	0.000****
168h, CA5 – 72h, LF-89^T	-1.360910766	0.122342996	-11.12373255	0.000****
168h, CA5 – 96h, LF-89^T	-1.751778761	0.122342996	-14.31858643	0.000****
168h, CA5 – 120h, LF-89^T	-1.181236725	0.122342996	-9.655123424	0.000****
168,h CA5 – 144h, LF-89^T	-1.009633648	0.122342996	-8.252484259	0.000****
168h, CA5 – 168h, LF-89^T	-0.627933568	0.122342996	-5.132566544	0.000***
168h, CA5 – 192h, LF-89^T	-0.703548385	0.122342996	-5.750622498	0.000****
168h, CA5 – 216h, LF-89^T	-0.874130083	0.122342996	-7.144913172	0.000****
168h, CA5 – 312h, LF-89^T	-0.616184451	0.122342996	-5.036532305	0.0001***

192h, CA5 – 216h, CA5	-0.53353328	0.122342996	-4.36096301	0.004**
192h, CA5 – 240h, CA5	-0.712456015	0.122342996	-5.823431164	0.000****
192h, CA5 – 24h, LF-89^{T}	-1.647009344	0.122342996	-13.46222832	0.000****
192h, CA5 – 48h, LF-89^{T}	-2.388007619	0.122342996	-19.51895654	0.000****
192h, CA5 – 72h, LF-89^{T}	-1.945502638	0.122342996	-15.90203529	0.000****
192h, CA5 – 96h, LF-89^{T}	-2.336370633	0.122342996	-19.09688917	0.000****
192h, CA5 – 120h, LF-89^{T}	-1.765828598	0.122342996	-14.43342617	0.000****
192h, CA5 – 144h, LF-89^{T}	-1.594225521	0.122342996	-13.030787	0.000****
192h, CA5 – 168h, LF-89^{T}	-1.212525441	0.122342996	-9.910869287	0.000****
192h, CA5 – 192h, LF-89^{T}	-1.288140258	0.122342996	-10.52892524	0.000****
192h, CA5 – 216h, LF-89^{T}	-1.458721956	0.122342996	-11.92321591	0.000****
192h, CA5 – 240h, LF-89^{T}	-0.853095227	0.122342996	-6.972979699	0.000****
192h, CA5 – 288h, LF-89^{T}	-0.857291992	0.122342996	-7.007282977	0.000****
192h, CA5 – 312h, LF-89^{T}	-1.200776324	0.122342996	-9.814835048	0.000****
192h, CA5 – 336h, LF-89^{T}	-0.61976439	0.122342996	-5.065793799	0.000***
216h, CA5 – 288h, CA5	0.478606668	0.122342996	3.912007093	0.028*
216h, CA5 – 24h, LF-89^{T}	-1.113476065	0.122342996	-9.101265314	0.000****
216h, CA5 – 48h, LF-89^{T}	-1.85447434	0.122342996	-15.15799353	0.000****
216h, CA5 – 72h, LF-89^{T}	-1.411969359	0.122342996	-11.54107228	0.000****
216h, CA5 – 96h, LF-89^{T}	-1.802837354	0.122342996	-14.73592616	0.000****

216h, CA5 – 120h, LF-89^T	-1.232295319	0.122342996	-10.07246316	0.000****
216h, CA5 – 144h, LF-89^T	-1.060692241	0.122342996	-8.669823992	0.000****
216h, CA5 – 168h, LF-89^T	-0.678992161	0.122342996	-5.549906277	0.000****
216h, CA5 – 192h, LF-89^T	-0.754606978	0.122342996	-6.167962231	0.000****
216h, CA5 – 216h, LF-89^T	-0.925188676	0.122342996	-7.562252905	0.000****
216h, CA5 – 312h, LF-89^T	-0.667243044	0.122342996	-5.453872038	0.000****
240h, CA5 – 288h, CA5	0.657529403	0.122342996	5.374475247	0.000****
240h, CA5 – 312h, CA5	0.551664865	0.122342996	4.509165901	0.002**
240h, CA5 – 336h, CA5	0.582181421	0.122342996	4.75860033	0.000***
240h, CA5 – 24h, LF-89^T	-0.93455333	0.122342996	-7.63879716	0.000****
240h, CA5 – 48h, LF-89^T	-1.675551604	0.122342996	-13.69552537	0.000****
240h, CA5 – 72h, LF-89^T	-1.233046624	0.122342996	-10.07860413	0.000****
240h, CA5 – 96h, LF-89^T	-1.623914618	0.122342996	-13.27345801	0.000****
240h, CA5 – 120h, LF-89^T	-1.053372583	0.122342996	-8.609995003	0.000****
240h, CA5 – 144h, LF-89^T	-0.881769506	0.122342996	-7.207355838	0.000****
240h, CA5 – 168h, LF-89^T	-0.500069426	0.122342996	-4.087438124	0.014*
240h, CA5 – 192h, LF-89^T	-0.575684243	0.122342996	-4.705494077	0.000***
240h, CA5 – 216h, LF-89^T	-0.746265941	0.122342996	-6.099784751	0.000****
240h, CA5 – 312h, LF-89^T	-0.488320309	0.122342996	-3.991403884	0.020*
240h, CA5 – 360h, LF-89^T	0.5713831	0.122342996	4.67033765	0.001**

264h, CA5 – 24h, LF-89^T	-1.360292377	0.122342996	-11.118678	0.000****
264h, CA5 – 48h, LF-89^T	-2.101290652	0.122342996	-17.17540621	0.000****
264h, CA5 – 72h, LF-89^T	-1.658785671	0.122342996	-13.55848497	0.000****
264h, CA5 – 96h, LF-89^T	-2.049653666	0.122342996	-16.75333885	0.000****
264h, CA5 – 120h, LF-89^T	-1.479111631	0.122342996	-12.08987585	0.000****
264h, CA5 – 144h, LF-89^T	-1.307508553	0.122342996	-10.68723668	0.000****
264h, CA5 – 168h, LF-89^T	-0.925808473	0.122342996	-7.567318967	0.000****
264h, CA5 – 192h, LF-89^T	-1.00142329	0.122342996	-8.185374921	0.000****
264h, CA5 – 216h, LF-89^T	-1.172004988	0.122342996	-9.579665594	0.000****
264h, CA5 – 240h, LF-89^T	-0.566378259	0.122342996	-4.629429379	0.001**
264h, CA5 – 288h, LF-89^T	-0.570575025	0.122342996	-4.663732657	0.001**
264h, CA5 – 312h, LF-89^T	-0.914059357	0.122342996	-7.471284728	0.000****
288h, CA5 – 24h, LF-89^T	-1.592082733	0.122342996	-13.01327241	0.000****
288h, CA5 – 48h, LF-89^T	-2.333081007	0.122342996	-19.07000062	0.000****
288h, CA5 – 72h, LF-89^T	-1.890576027	0.122342996	-15.45307938	0.000****
288h, CA5 – 96h, LF-89^T	-2.281444022	0.122342996	-18.64793325	0.000****
288h, CA5 – 120h, LF-89^T	-1.710901986	0.122342996	-13.98447025	0.000****
288h, CA5 – 144h, LF-89^T	-1.539298909	0.122342996	-12.58183109	0.000****
288h, CA5 – 168h, LF-89^T	-1.157598829	0.122342996	-9.46191337	0.000****
288h, CA5 – 192h, LF-89^T	-1.233213646	0.122342996	-10.07996932	0.000****

288h, CA5 – 216h, LF-89^T	-1.403795344	0.122342996	-11.47426	0.000****
288h, CA5 – 240h, LF-89^T	-0.798168615	0.122342996	-6.524023782	0.000****
288h, CA5 – 288h, LF-89^T	-0.802365381	0.122342996	-6.55832706	0.000****
288h, CA5 – 312h, LF-89^T	-1.145849712	0.122342996	-9.365879131	0.000****
288h, CA5 – 336h, LF-89^T	-0.564837778	0.122342996	-4.616837881	0.001**
312h, CA5 – 24h, LF-89^T	-1.486218195	0.122342996	-12.14796306	0.000****
312h, CA5 – 48h, LF-89^T	-2.227216469	0.122342996	-18.20469127	0.000****
312h, CA5 – 72h, LF-89^T	-1.784711489	0.122342996	-14.58777003	0.000****
312h, CA5 – 96h, LF-89^T	-2.175579484	0.122342996	-17.78262391	0.000****
312h, CA5 – 120h, LF-89^T	-1.605037449	0.122342996	-13.1191609	0.000****
312h, CA5 – 144h, LF-89^T	-1.433434371	0.122342996	-11.71652174	0.000****
312h, CA5 – 168h, LF-89^T	-1.051734291	0.122342996	-8.596604024	0.000****
312h, CA5 – 192h, LF-89^T	-1.127349108	0.122342996	-9.214659978	0.000****
312h, CA5 – 216h, LF-89^T	-1.297930806	0.122342996	-10.60895065	0.000****
312h, CA5 – 240h, LF-89^T	-0.692304077	0.122342996	-5.658714436	0.000****
312h, CA5 – 288h, LF-89^T	-0.696500843	0.122342996	-5.693017714	0.000****
312h, CA5 – 312h, LF-89^T	-1.039985174	0.122342996	-8.500569785	0.000****
312h, CA5 – 336h, LF-89^T	-0.45897324	0.122342996	-3.751528535	0.049*
336h, CA5 – 24h, LF-89^T	-1.51673475	0.122342996	-12.39739749	0.000****
336h, CA5 – 48h, LF-89^T	-2.257733025	0.122342996	-18.4541257	0.000****

336h, CA5 – 72h, LF-89^T	-1.815228044	0.122342996	-14.83720446	0.000****
336h, CA5 – 96h, LF-89^T	-2.206096039	0.122342996	-18.03205834	0.000****
336h, CA5 – 120h, LF-89^T	-1.635554004	0.122342996	-13.36859533	0.000****
336h, CA5 – 144h, LF-89^T	-1.463950926	0.122342996	-11.96595617	0.000****
336h, CA5 – 168h, LF-89^T	-1.082250846	0.122342996	-8.846038454	0.000****
336h, CA5 – 192h, LF-89^T	-1.157865663	0.122342996	-9.464094408	0.000****
336h, CA5 – 216h, LF-89^T	-1.328447361	0.122342996	-10.85838508	0.000****
336h, CA5 – 240h, LF-89^T	-0.722820632	0.122342996	-5.908148866	0.000****
336h, CA5 – 288h, LF-89^T	-0.727017398	0.122342996	-5.942452144	0.000****
336h, CA5 – 312h, LF-89^T	-1.07050173	0.122342996	-8.750004215	0.000****
336h, CA5 – 336h, LF-89^T	-0.489489796	0.122342996	-4.000962965	0.020*
360h, CA5 – 24h, LF-89^T	-1.37447698	0.122342996	-11.23461927	0.000****
360h, CA5 – 48h, LF-89^T	-2.115475254	0.122342996	-17.29134748	0.000****
360h, CA5 – 72h, LF-89^T	-1.672970274	0.122342996	-13.67442624	0.000****
360h, CA5 – 96h, LF-89^T	-2.063838268	0.122342996	-16.86928012	0.000****
360h, CA5 – 120h, LF-89^T	-1.493296233	0.122342996	-12.20581712	0.000****
360h, CA5 – 144h, LF-89^T	-1.321693156	0.122342996	-10.80317795	0.000****
360h, CA5 – 168h, LF-89^T	-0.939993076	0.122342996	-7.683260237	0.000****
360h, CA5 – 192h, LF-89^T	-1.015607893	0.122342996	-8.301316191	0.000****
360h, CA5 – 216h, LF-89^T	-1.186189591	0.122342996	-9.695606864	0.000****

360h, CA5 – 240h, LF-89^T	-0.580562862	0.122342996	-4.745370649	0.000***
360h, CA5 – 288h, LF-89^T	-0.584759628	0.122342996	-4.779673926	0.000***
360h, CA5 – 312h, LF-89^T	-0.928243959	0.122342996	-7.587225997	0.000****
24h, LF-89^T – 48h, LF-89^T	-0.740998275	0.122342996	-6.056728212	0.000****
24h, LF-89^T – 96h, LF-89^T	-0.689361289	0.122342996	-5.634660848	0.000****
24h, LF-89^T – 240h, LF-89^T	0.793914118	0.122342996	6.489248624	0.000****
24h, LF-89^T – 264h, LF-89^T	1.280580721	0.122342996	10.46713555	0.000****
24h, LF-89^T – 288h, LF-89^T	0.789717352	0.122342996	6.454945346	0.000****
24h, LF-89^T – 336h, LF-89^T	1.027244955	0.122342996	8.396434525	0.000****
24h, LF-89^T – 360h, LF-89^T	1.505936429	0.122342996	12.30913481	0.000****
48h, LF-89^T – 120h, LF-89^T	0.622179021	0.122342996	5.085530368	0.000***
48h, LF-89^T – 144h, LF-89^T	0.793782099	0.122342996	6.488169533	0.000****
48h, LF-89^T – 168h, LF-89^T	1.175482179	0.122342996	9.608087248	0.000****
48h, LF-89^T – 192h, LF-89^T	1.099867362	0.122342996	8.990031294	0.000****
48h, LF-89^T – 216h, LF-89^T	0.929285664	0.122342996	7.595740621	0.000****
48h, LF-89^T – 240h, LF-89^T	1.534912392	0.122342996	12.54597684	0.000****
48h, LF-89^T – 264h, LF-89^T	2.021578996	0.122342996	16.52386376	0.000****
48h, LF-89^T – 288h, LF-89^T	1.530715627	0.122342996	12.51167356	0.000****
48h, LF-89^T – 312h, LF-89^T	1.187231295	0.122342996	9.704121487	0.000****
48h, LF-89^T – 336h, LF-89^T	1.768243229	0.122342996	14.45316274	0.000****

48h, LF-89^T – 360h, LF-89^T	2.246934704	0.122342996	18.36586302	0.000****
72h, LF-89^T – 168h, LF-89^T	0.732977198	0.122342996	5.991166005	0.000****
72h, LF-89^T – 192h, LF-89^T	0.657362381	0.122342996	5.373110051	0.000****
72h, LF-89^T – 216h, LF-89^T	0.486780683	0.122342996	3.978819378	0.021*
72h, LF-89^T – 240h, LF-89^T	1.092407412	0.122342996	8.929055593	0.000****
72h, LF-89^T – 264h, LF-89^T	1.579074015	0.122342996	12.90694252	0.000****
72h, LF-89^T – 288h, LF-89^T	1.088210646	0.122342996	8.894752316	0.000****
72h, LF-89^T – 312h, LF-89^T	0.744726315	0.122342996	6.087200245	0.000****
72h, LF-89^T – 336h, LF-89^T	1.325738249	0.122342996	10.83624149	0.000****
72h, LF-89^T – 360h, LF-89^T	1.804429723	0.122342996	14.74894178	0.000****
96h, LF-89^T – 120h, LF-89^T	0.570542035	0.122342996	4.663463004	0.001**
96h, LF-89^T – 144h, LF-89^T	0.742145113	0.122342996	6.066102169	0.000****
96h, LF-89^T – 168h, LF-89^T	1.123845193	0.122342996	9.186019884	0.000****
96h, LF-89^T – 192h, LF-89^T	1.048230376	0.122342996	8.56796393	0.000****
96h, LF-89^T – 216h, LF-89^T	0.877648678	0.122342996	7.173673257	0.000****
96h, LF-89^T – 240h, LF-89^T	1.483275407	0.122342996	12.12390947	0.000****
96h, LF-89^T – 264h, LF-89^T	1.96994201	0.122342996	16.10179639	0.000****
96h, LF-89^T – 288h, LF-89^T	1.479078641	0.122342996	12.08960619	0.000****
96h, LF-89^T – 312h, LF-89^T	1.135594309	0.122342996	9.282054123	0.000****
96h, LF-89^T – 336h, LF-89^T	1.716606244	0.122342996	14.03109537	0.000****

96h, LF-89^T – 360h, LF-89^T	2.195297718	0.122342996	17.94379566	0.000****
120h, LF-89^T – 168h, LF-89^T	0.553303158	0.122342996	4.52255688	0.002**
120h, LF-89^T – 192h, LF-89^T	0.477688341	0.122342996	3.904500926	0.028*
120h, LF-89^T – 240h, LF-89^T	0.912733372	0.122342996	7.460446468	0.000****
120h, LF-89^T – 264h, LF-89^T	1.399399975	0.122342996	11.43833339	0.000****
120h, LF-89^T – 288h, LF-89^T	0.908536606	0.122342996	7.42614319	0.000****
120h, LF-89^T – 312h, LF-89^T	0.565052274	0.122342996	4.618591119	0.001**
120h, LF-89^T – 336h, LF-89^T	1.146064208	0.122342996	9.367632369	0.000****
120h, LF-89^T – 360h, LF-89^T	1.624755683	0.122342996	13.28033265	0.000****
144h, LF-89^T – 240h, LF-89^T	0.741130294	0.122342996	6.057807303	0.000****
144h, LF-89^T – 264h, LF-89^T	1.227796897	0.122342996	10.03569423	0.000****
144h, LF-89^T – 288h, LF-89^T	0.736933528	0.122342996	6.023504025	0.000****
144h, LF-89^T – 336h, LF-89^T	0.974461131	0.122342996	7.964993204	0.000****
144h, LF-89^T – 360h, LF-89^T	1.453152606	0.122342996	11.87769349	0.000****
168h, LF-89^T – 264h, LF-89^T	0.846096817	0.122342996	6.915776511	0.000****
168h, LF-89^T – 336h, LF-89^T	0.592761051	0.122342996	4.845075489	0.000***
168h, LF-89^T – 360h, LF-89^T	1.071452526	0.122342996	8.757775774	0.000****
192h, LF-89^T – 264h, LF-89^T	0.921711634	0.122342996	7.533832465	0.000****
192h, LF-89^T – 336h, LF-89^T	0.668375868	0.122342996	5.463131443	0.000****
192h, LF-89^T – 360h, LF-89^T	1.147067343	0.122342996	9.375831728	0.000****

216h, LF-89^T – 240h, LF-89^T	0.605626729	0.122342996	4.950236215	0.000***
216h, LF-89^T – 264h, LF-89^T	1.092293332	0.122342996	8.928123138	0.000****
216h, LF-89^T – 288h, LF-89^T	0.601429963	0.122342996	4.915932937	0.000***
216h, LF-89^T – 336h, LF-89^T	0.838957566	0.122342996	6.857422116	0.000****
216h, LF-89^T – 360h, LF-89^T	1.317649041	0.122342996	10.7701224	0.000****
240h, LF-89^T – 264h, LF-89^T	0.486666603	0.122342996	3.977886923	0.021*
240h, LF-89^T – 360h, LF-89^T	0.712022312	0.122342996	5.819886186	0.000****
264h, LF-89^T – 288h, LF-89^T	-0.490863369	0.122342996	-4.012190201	0.019*
264h, LF-89^T – 312h, LF-89^T	-0.834347701	0.122342996	-6.819742272	0.000****
288h, LF-89^T – 360h, LF-89^T	0.716219077	0.122342996	5.854189463	0.000****
312h, LF-89^T – 336h, LF-89^T	0.581011934	0.122342996	4.749041249	0.000***
312h, LF-89^T – 360h, LF-89^T	1.059703409	0.122342996	8.661741534	0.000****
336h, LF-89^T – 360h, LF-89^T	0.478691475	0.122342996	3.912700285	0.027*

Supplementary Table S4. Tukey's multiple comparison test (HSD) for fluorescence ratios of live-to-dead bacteria in *P. salmonis* biofilms between two contrasted conditions. Only statistically significant condition comparisons are shown: '****'P ≤ 0.0001; '***'P ≤ 0.001; '**'P ≤ 0.01; and '*'P ≤ 0.05. Standard error (SE).

Contrast	Estimate	SE	z-ratio	p-value
24h, CA5 – 24h, LF-89^T	0.211977271	0.030440763	6.963599183	0.000****
24h, CA5 – 48h, LF-89^T	0.29153075	0.029925833	9.741775479	0.000****
24h, CA5 – 72h, LF-89^T	0.308856846	0.029754467	10.38018412	0.000****
24h, CA5 – 96h, LF-89^T	0.161373943	0.0305425	5.283586584	0.000****
24h, CA5 – 120h, CA5	0.153440792	0.03054276	5.023802353	0.000***
24h, CA5 – 120h, LF-89^T	0.201561397	0.030475883	6.613799994	0.000****
24h, CA5 – 144h, CA5	0.21817242	0.03041637	7.172861929	0.000****
24h, CA5 – 144h, LF-89^T	0.267398349	0.030128721	8.875197394	0.000****
24h, CA5 – 168h, CA5	0.278565288	0.030039972	9.273154113	0.000****
24h, CA5 – 168h, LF-89^T	0.383336269	0.028762625	13.32758268	0.000****
24h, CA5 – 192h, CA5	0.376298555	0.028874723	13.03210978	0.000****
24h, CA5 – 192h, LF-89^T	0.328920471	0.029528639	11.13903255	0.000****
24h, CA5 – 216h, CA5	0.28306452	0.030001721	9.434942885	0.000****
24h, CA5 – 216h, LF-89^T	0.329841068	0.029517562	11.1744008	0.000****
24h, CA5 – 240h, CA5	0.332040051	0.029490847	11.25908848	0.000****
24h, CA5 – 240h, LF-89^T	0.398833553	0.028501634	13.99335733	0.000****

24h, CA5 – 264h, CA5	0.411743636	0.028269044	14.56517706	0.000****
24h, CA5 – 264h, LF-89^T	0.43009014	0.027914085	15.40763856	0.000****
24h, CA5 – 288h, CA5	0.379659622	0.028821683	13.17270842	0.000****
24h, CA5 – 288h, LF-89^T	0.372426712	0.028934707	12.87127986	0.000****
24h, CA5 – 312h, CA5	0.404154155	0.028407469	14.22703851	0.000****
24h, CA5 – 312h, LF-89^T	0.383165656	0.02876539	13.3203706	0.000****
24h, CA5 – 336h, CA5	0.444198939	0.027621004	16.08192575	0.000****
24h, CA5 – 336h, LF-89^T	0.42313738	0.028052032	15.08401869	0.000****
24h, CA5 – 360h, CA5	0.464604947	0.027164958	17.10309848	0.000****
24h, CA5 – 360h, LF-89^T	0.468179815	0.027081033	17.28810789	0.000****
24h, LF-89^T – 48h, CA5	-0.147611688	0.030977914	-4.765062292	0.000***
24h, LF-89^T – 96h, CA5	-0.158450463	0.030907451	-5.126610431	0.000***
24h, LF-89^T – 168h, LF-89^T	0.171358998	0.029516917	5.805450397	0.000****
24h, LF-89^T – 192h, CA5	0.164321284	0.02962632	5.546462828	0.000****
24h, LF-89^T – 192h, LF-89^T	0.1169432	0.03026506	3.863967277	0.033*
24h, LF-89^T – 216h, LF-89^T	0.117863797	0.030254232	3.895778904	0.029*
24h, LF-89^T – 240h, CA5	0.12006278	0.030228118	3.97189069	0.022*
24h, LF-89^T – 240h, LF-89^T	0.186856282	0.029262299	6.385564028	0.000****
24h, LF-89^T – 264h, CA5	0.199766365	0.029035505	6.88007192	0.000****
24h, LF-89^T – 264h, LF-89^T	0.218112869	0.028689605	7.602505011	0.000****

24h, LF-89^T – 288h, CA5	0.167682351	0.029574552	5.669818845	0.000****
24h, LF-89^T – 288h, LF-89^T	0.160449441	0.029684874	5.405090884	0.000****
24h, LF-89^T – 312h, CA5	0.192176884	0.029170467	6.588063359	0.000****
24h, LF-89^T – 312h, LF-89^T	0.171188385	0.029519615	5.799140176	0.000****
24h, LF-89^T – 336h, CA5	0.232221668	0.028404199	8.175610553	0.000****
24h, LF-89^T – 336h, LF-89^T	0.21116011	0.028824001	7.325843089	0.000****
24h, LF-89^T – 360h, CA5	0.252627676	0.027960451	9.035178771	0.000****
24h, LF-89^T – 360h, LF-89^T	0.256202544	0.027878838	9.189857414	0.000****
48h, CA5 – 48h, LF-89^T	0.227165168	0.030471523	7.454998803	0.000****
48h, CA5 – 72h, LF-89^T	0.244491263	0.030303125	8.068186525	0.000****
48h, CA5 – 120h, LF-89^T	0.137195814	0.031012495	4.423888258	0.003**
48h, CA5 – 144h, CA5	0.153806837	0.030953902	4.968899734	0.000***
48h, CA5 – 144h, LF-89^T	0.203032766	0.030670964	6.619706106	0.000****
48h, CA5 – 168h, CA5	0.214199706	0.030583712	7.003718273	0.000****
48h, CA5 – 168h, LF-89^T	0.318970687	0.029329323	10.87548771	0.000****
48h, CA5 – 192h, CA5	0.311932972	0.029439311	10.59579744	0.000****
48h, CA5 – 192h, LF-89^T	0.264554888	0.030081279	8.794668945	0.000****
48h, CA5 – 216h, CA5	0.218698937	0.030546112	7.159632611	0.000****
48h, CA5 – 216h, LF-89^T	0.265475485	0.030070399	8.828465665	0.000****
48h, CA5 – 240h. CA5	0.267674469	0.03004416	8.909367633	0.000****

48h, CA5 – 240h, LF-89^T	0.33446797	0.029073311	11.50429598	0.000****
48h, CA5 – 264h, CA5	0.347378053	0.02884524	12.04282071	0.000****
48h, CA5 – 264h, LF-89^T	0.365724557	0.028497329	12.83364324	0.000****
48h, CA5 – 288h, CA5	0.315294039	0.029387266	10.72893393	0.000****
48h, CA5 – 288h, LF-89^T	0.308061129	0.029498174	10.44339665	0.000****
48h, CA5 – 312h, CA5	0.339788572	0.028980966	11.72454282	0.000****
48h, CA5 – 312h, LF-89^T	0.318800073	0.029332035	10.86866543	0.000****
48h, CA5 – 336h, CA5	0.379833356	0.028210208	13.46439407	0.000****
48h, CA5 – 336h, LF-89^T	0.358771798	0.028632515	12.53022293	0.000****
48h, CA5 – 360h, CA5	0.400239365	0.027763696	14.41592544	0.000****
48h, CA5 – 360h, LF-89^T	0.403814232	0.02768156	14.58784212	0.000****
48h, LF-89^T – 72h, CA5	-0.182198246	0.03068244	-5.938192957	0.000****
48h, LF-89^T – 96h, CA5	-0.238003942	0.030399978	-7.829082628	0.000****
48h, LF-89^T – 96h, LF-89^T	-0.130156808	0.030756122	-4.23189917	0.008**
48h, LF-89^T – 120h, CA5	-0.138089959	0.030756623	-4.489763292	0.002**
48h, LF-89^T – 264h, CA5	0.120212885	0.028491505	4.219253651	0.008**
48h, LF-89^T – 264h, LF-89^T	0.138559389	0.028138756	4.924147696	0.000***
48h, LF-89^T – 312h, CA5	0.112623405	0.028629097	3.933878991	0.025*
48h, LF-89^T – 336h, CA5	0.152668189	0.027847579	5.482278746	0.000****
48h, LF-89^T – 336h, LF-89^T	0.13160663	0.028275831	4.654385927	0.001**

48h, LF-89^T – 360h, CA5	0.173074197	0.027394633	6.317813946	0.000****
48h, LF-89^T – 360h, LF-89^T	0.176649065	0.027311297	6.467985151	0.000****
72h, CA5 – 72h, LF-89^T	0.199524342	0.030515123	6.538539592	0.000****
72h, CA5 – 144h, LF-89^T	0.158065845	0.030880631	5.118607979	0.000***
72h, CA5 – 168h, CA5	0.169232784	0.030793921	5.495655528	0.000****
72h, CA5 – 168h, LF-89^T	0.274003765	0.029547947	9.273191437	0.000****
72h, CA5 – 192h, CA5	0.266966051	0.029657158	9.001740921	0.000****
72h, CA5 – 192h, LF-89^T	0.219587967	0.030294734	7.248387284	0.000****
72h, CA5 – 216h, CA5	0.173732016	0.030756556	5.648617268	0.000****
72h, CA5 – 216h, LF-89^T	0.220508564	0.030283927	7.281372886	0.000****
72h, CA5 – 240h, CA5	0.222707547	0.030257863	7.360319826	0.000****
72h, CA5 – 240h, LF-89^T	0.289501049	0.02929377	9.8826832	0.000****
72h, CA5 – 264h, CA5	0.302411132	0.029067366	10.40380231	0.000****
72h, CA5 – 264h, LF-89^T	0.320757636	0.028722056	11.16764187	0.000****
72h, CA5 – 288h, CA5	0.270327118	0.02960548	9.130982346	0.000****
72h, CA5 – 288h, LF-89^T	0.263094208	0.029715608	8.853737991	0.000****
72h, CA5 – 312h, CA5	0.294821651	0.029202096	10.09590708	0.000****
72h, CA5 – 312h, LF-89^T	0.273833152	0.02955064	9.266572732	0.000****
72h, CA5 – 336h, CA5	0.334866435	0.028437134	11.77567446	0.000****
72h, CA5 – 336h, LF-89^T	0.313804877	0.028856223	10.87477302	0.000****

72h, CA5 – 360h, CA5	0.355272443	0.02799414	12.69095747	0.000****
72h, CA5 – 360h, LF-89^T	0.358847311	0.027912666	12.85607462	0.000****
72h, LF-89^T – 96h, CA5	-0.255330038	0.030231202	-8.445911014	0.000****
72h, LF-89^T – 96h, LF-89^T	-0.147482903	0.030589114	-4.821417918	0.000***
72h, LF-89^T – 120h, CA5	-0.155416054	0.030589633	-5.080677369	0.000***
72h, LF-89^T – 264h, LF-89^T	0.121233294	0.027955585	4.336639451	0.005**
72h, LF-89^T – 336h, CA5	0.135342093	0.027662453	4.892628092	0.000***
72h, LF-89^T – 336h, LF-89^T	0.114280535	0.028093568	4.067854107	0.015*
72h, LF-89^T – 360h, CA5	0.155748101	0.027206386	5.7246892	0.000****
72h, LF-89^T – 360h, LF-89^T	0.159322969	0.027122464	5.87420706	0.000****
96h, CA5 – 120h, LF-89^T	0.148034589	0.0309421	4.784245028	0.000***
96h, CA5 – 144h, CA5	0.164645612	0.030883392	5.331202282	0.000****
96h, CA5 – 144h, LF-89^T	0.213871541	0.030599858	6.989298488	0.000****
96h, CA5 – 168h, CA5	0.22503848	0.030512416	7.375308469	0.000****
96h, CA5 – 168h, LF-89^T	0.329809461	0.029255093	11.27357408	0.000****
96h, CA5 – 192h,CA5	0.322771747	0.029365351	10.99158478	0.000****
96h, CA5 – 192h,LF-89^T	0.275393663	0.030008847	9.177082446	0.000****
96h, CA5 – 216h,CA5	0.229537712	0.030474732	7.532066523	0.000****
96h, CA5 – 216h, LF-89^T	0.27631426	0.029997942	9.211107203	0.000****
96h, CA5 – 240h, CA5	0.278513243	0.029971642	9.292558617	0.000****

96h, CA5 – 240h, LF-89^T	0.345306745	0.028998444	11.9077679	0.000****
96h, CA5 – 264h, CA5	0.358216828	0.028769796	12.45114262	0.000****
96h, CA5 – 264h, LF-89^T	0.376563332	0.028420983	13.24948289	0.000****
96h, CA5 – 288h, CA5	0.326132814	0.02931318	11.12580822	0.000****
96h, CA5 – 288h, LF-89^T	0.318899904	0.029424358	10.83795632	0.000****
96h, CA5 – 312h, CA5	0.350627347	0.028905866	12.12997186	0.000****
96h, CA5 – 312h, LF-89^T	0.329638848	0.029257812	11.26669502	0.000****
96h, CA5 – 336h, CA5	0.390672131	0.0281331	13.88656546	0.000****
96h, CA5 – 336h, LF-89^T	0.369610573	0.028556522	12.94312271	0.000****
96h, CA5 – 360h, CA5	0.411078139	0.027685368	14.84820911	0.000****
96h, CA5 – 360h, LF-89^T	0.414653007	0.027603004	15.02202441	0.000****
96h, LF-89^T – 168h, CA5	0.117191346	0.030867408	3.796604655	0.042*
96h, LF-89^T – 168h, LF-89^T	0.221962327	0.029623935	7.492668565	0.000****
96h, LF-89^T – 192h, CA5	0.214924612	0.029732906	7.228510067	0.000****
96h, LF-89^T – 192h, LF-89^T	0.167546529	0.030369152	5.516997285	0.000****
96h, LF-89^T – 216h, CA5	0.121690577	0.030830108	3.947134331	0.024*
96h, LF-89^T – 216h, LF-89^T	0.168467126	0.030358366	5.549281745	0.000****
96h, LF-89^T – 240h, CA5	0.170666109	0.030332354	5.626536955	0.000****
96h, LF-89^T – 240h, LF-89^T	0.23745961	0.029370329	8.085017032	0.000****
96h, LF-89^T – 264h, CA5	0.250369693	0.029144447	8.590648237	0.000****

96h, LF-89^T – 264h, LF-89^T	0.268716197	0.028799957	9.330437453	0.000****
96h, LF-89^T – 288h,CA5	0.218285679	0.029681342	7.354306281	0.000****
96h, LF-89^T – 288h, LF-89^T	0.211052769	0.029791229	7.084392731	0.000****
96h, LF-89^T – 312h, CA5	0.242780212	0.029278865	8.291995318	0.000****
96h, LF-89^T – 312h, LF-89^T	0.221791714	0.029626622	7.486230194	0.000****
96h, LF-89^T – 336h, CA5	0.282824997	0.028515733	9.918208845	0.000****
96h, LF-89^T – 336h, LF-89^T	0.261763438	0.028933802	9.046976883	0.000****
96h, LF-89^T – 360h, CA5	0.303231005	0.028073862	10.80118583	0.000****
96h, LF-89^T – 360h, LF-89^T	0.306805872	0.027992599	10.96024949	0.000****
120h, CA5 – 168h, CA5	0.125124497	0.030867897	4.05354783	0.016*
120h, CA5 – 168h, LF-89^T	0.229895478	0.029624534	7.760306939	0.000****
120h, CA5 – 192h, CA5	0.222857763	0.029733497	7.495174945	0.000****
120h, CA5 – 192h, LF-89^T	0.17547968	0.030369691	5.778118719	0.000****
120h, CA5 – 216h, CA5	0.129623728	0.030830601	4.204385393	0.009**
120h, CA5 – 216h, LF-89^T	0.176400277	0.030358906	5.810495193	0.000****
120h, CA5 – 240h, CA5	0.17859926	0.030332896	5.887972586	0.000****
120h, CA5 – 240h, LF-89^T	0.245392761	0.029370947	8.354949024	0.000****
120h, CA5 – 264h, CA5	0.258302844	0.029145081	8.862656728	0.000****
120h, CA5 – 264h, LF-89^T	0.276649348	0.028800614	9.605675401	0.000****
120h, CA5 – 288h, CA5	0.22621883	0.029681937	7.621430916	0.000****

120h, CA5 – 288h, LF-89^T	0.21898592	0.029791816	7.350539517	0.000****
120h, CA5 – 312h, CA5	0.250713363	0.029279489	8.562764267	0.000****
120h, CA5 – 312h, LF-89^T	0.229724865	0.029627221	7.753844476	0.000****
120h, CA5 – 336h, CA5	0.290758147	0.028516409	10.19616984	0.000****
120h, CA5 – 336h, LF-89^T	0.269696589	0.02893445	9.320950976	0.000****
120h, CA5 – 360h, CA5	0.311164156	0.028074567	11.08348921	0.000****
120h, CA5 – 360h, LF-89^T	0.314739023	0.027993309	11.24336631	0.000****
120h, LF-89^T - LF89,168	0.181774872	0.02955357	6.150690762	0.000****
120h, LF-89^T - CA5,192	0.174737158	0.02966283	5.890778301	0.000****
120h, LF-89^T - LF89,192	0.127359074	0.030300748	4.203165977	0.009**
120h, LF-89^T - LF89,216	0.128279671	0.030289934	4.235059453	0.007**
120h, LF-89^T - CA5,240	0.130478654	0.030263853	4.31136947	0.005**
120h, LF-89^T - LF89,240	0.197272156	0.029299288	6.733001719	0.000****
120h, LF-89^T - CA5,264	0.210182238	0.029072798	7.229515406	0.000****
120h, LF-89^T - LF89,264	0.228528742	0.028727368	7.955088164	0.000****
120h, LF-89^T - CA5,288	0.178098225	0.029611129	6.014570454	0.000****
120h, LF-89^T - LF89,288	0.170865315	0.029721308	5.748916439	0.000****
120h, LF-89^T - CA5,312	0.202592758	0.029207578	6.936307942	0.000****
120h, LF-89^T - LF89,312	0.181604259	0.029556265	6.144357589	0.000****
120h, LF-89^T - CA5,336	0.242637542	0.028442356	8.53085237	0.000****

120h, LF-89^T - LF89,336	0.221575983	0.02886158	7.677195274	0.000****
120h, LF-89^T - CA5,360	0.26304355	0.027999237	9.394668546	0.000****
120h, LF-89^T - LF89,360	0.266618418	0.027917741	9.550143031	0.000****
144h, CA5 - 168h, LF-89^T	0.165163849	0.029491501	5.600388049	0.000****
144h, CA5 - 192h, CA5	0.158126135	0.029601003	5.341918206	0.000****
144h, CA5 - 240h, CA5	0.113867631	0.030203334	3.770035189	0.046*
144h, CA5 - 240h, LF-89^T	0.180661133	0.029236651	6.179269118	0.000****
144h, CA5 - 264h, CA5	0.193571215	0.029009648	6.67264956	0.000****
144h, CA5 - 264h, LF-89^T	0.211917719	0.028663424	7.393314975	0.000****
144h, CA5 - 288h, CA5	0.161487202	0.029549188	5.465030181	0.000****
144h, CA5 - 288h, LF-89^T	0.154254292	0.029659609	5.20082018	0.000****
144h, CA5 - 312h, CA5	0.185981735	0.029144734	6.381315119	0.000****
144h, CA5 - 312h, LF-89^T	0.164993236	0.029494201	5.594090684	0.000****
144h, CA5 - 336h, CA5	0.226026519	0.028377744	7.964921983	0.000****
144h, CA5 - 336h, LF-89^T	0.20496496	0.028797946	7.117346431	0.000****
144h, CA5 - 360h, CA5	0.246432527	0.027933562	8.822094481	0.000****
144h, CA5 - 360h, LF-89^T	0.250007395	0.027851868	8.976324241	0.000****
144h, LF-89^T – 168h, LF-89^T	0.115937921	0.029192664	3.97147449	0.022*
144h, LF-89^T – 240h, LF-89^T	0.131435204	0.028935098	4.542414269	0.002**
144h, LF-89^T – 264h, CA5	0.144345287	0.028705641	5.028464076	0.000***

144h, LF-89^T – 264h, LF-89^T	0.162691791	0.028355605	5.737553065	0.000****
144h, LF-89^T – 288h, CA5	0.112261273	0.02925096	3.837866326	0.036*
144h, LF-89^T – 312h, CA5	0.136755806	0.028842193	4.741519069	0.000***
144h, LF-89^T – 312h, LF-89^T	0.115767308	0.029195393	3.965259448	0.023*
144h, LF-89^T – 336h, CA5	0.17680059	0.028066716	6.299297393	0.000****
144h, LF-89^T – 336h, LF-89^T	0.155739032	0.028491619	5.466134841	0.000****
144h, LF-89^T – 360h, CA5	0.197206599	0.02761742	7.140659759	0.000****
144h, LF-89^T – 360h, LF-89^T	0.200781466	0.027534768	7.291925244	0.000****
168h, CA5 – 240h, LF-89^T	0.120268265	0.028842181	4.169874106	0.010*
168h, CA5 – 264h, CA5	0.133178347	0.028611963	4.654638566	0.001**
168h, CA5 – 264h, LF-89^T	0.151524851	0.028260744	5.361672374	0.000****
168h, CA5 – 312h, CA5	0.125588867	0.028748969	4.368465129	0.004**
168h, CA5 – 336h, CA5	0.165633651	0.027970857	5.921650829	0.000****
168h, CA5, - 336h, LF-89^T	0.144572092	0.028397221	5.091064846	0.000***
168h, CA5 – 360h, CA5	0.186039659	0.027519971	6.760169194	0.000****
168h, CA5 – 360h, LF-89^T	0.189614527	0.02743702	6.910900799	0.000****
192h, LF-89^T – 336h, CA5	0.115278468	0.027418364	4.204425463	0.009**
192h, LF-89^T – 360h, CA5	0.135684476	0.026958121	5.033157745	0.000***
192h, LF-89^T – 360h, LF-89^T	0.139259344	0.026873416	5.182048432	0.000****
216h, CA5 – 240h, LF-89^T	0.115769033	0.02880214	4.019459484	0.018*

216h, CA5 – 264h, CA5	0.128679116	0.028571592	4.503743211	0.002**
216h, CA5 – 264h, LF-89^T	0.14702562	0.028219862	5.210004938	0.000****
216h, CA5 – 312h, CA5	0.121089635	0.028708794	4.21785861	0.008**
216h, CA5 – 336h, CA5	0.161134419	0.027929544	5.769318003	0.000****
216h, CA5 – 336h, LF-89^T	0.140072861	0.028356539	4.939702351	0.000***
216h, CA5 – 360h, CA5	0.181540427	0.027477969	6.606762965	0.000****
216h, CA5 – 360h, LF-89^T	0.185115295	0.02739489	6.75729298	0.000****
216h, LF-89^T – 336h, CA5	0.114357871	0.027406387	4.172672321	0.010*
216h, LF-89^T – 360h, CA5	0.134763879	0.026945937	5.001268992	0.000***
216h, LF-89^T - 360h, LF-89^T	0.138338747	0.026861193	5.150134094	0.000***
240h, CA5 – 336h, CA5	0.112158888	0.027377498	4.096754546	0.013*
240h, CA5 – 360h, CA5	0.132564896	0.026916549	4.925033206	0.000***
240h, CA5 – 360h, LF-89^T	0.136139764	0.026831711	5.073838299	0.000***

Supplementary Table S5. Tukey's multiple comparison test (HSD) for LDH-based cytotoxicity values between two contrasted conditions. Only statistically significant condition comparisons are shown: '****'P ≤ 0.0001; '***'P ≤ 0.001; '**'P ≤ 0.01; and '*'P ≤ 0.05. Standard error (SE).

Contrast	Estimate	SE	z-ratio	p-value
0h, CA5, biofilm – 0h, LF-89^T, biofilm	-0.224180393	0.058783533	-3.813659757	0.027*
0h, CA5, biofilm – 6h, CA5, biofilm	-0.423226813	0.061368027	-6.896536053	0.000****
0h, CA5, biofilm – 6h, LF-89^T, biofilm	-0.230018926	0.058995668	-3.898912134	0.019*
0h, CA5, biofilm – 12h, LF-89^T, biofilm	-0.394948989	0.061564724	-6.415183331	0.000****
0h, CA5, biofilm – 24h, LF-89^T, biofilm	-0.395896983	0.061561121	-6.430957994	0.000****
0h, CA5, biofilm – 48h, CA5, biofilm	-0.444057564	0.061104322	-7.26720385	0.000****
0h, CA5, biofilm – 48h, LF-89^T, biofilm	-0.436873832	0.061206725	-7.137676976	0.000****
0h, CA5, biofilm – 72h, CA5, biofilm	-0.523224348	0.059154818	-8.844999673	0.000****
0h, CA5, biofilm – 72h, LF-89^T, biofilm	-0.471911473	0.060591931	-7.788355083	0.000****
0h, CA5, biofilm – 6h, CA5, planktonic	-0.410577886	0.061478868	-6.67835791	0.000****
0h, CA5, biofilm – 6h, LF-89^T, planktonic	-0.462578971	0.060784171	-7.610187997	0.000****
0h, CA5, biofilm – 12h, LF-89^T, planktonic	-0.360076024	0.06155348	-5.849807719	0.000****
0h, CA5, biofilm – 24h, LF-89^T, planktonic	-0.418734136	0.061411644	-6.818481118	0.000****
0h, CA5, biofilm – 48h, CA5, planktonic	-0.430732621	0.061284687	-7.028389021	0.000****
0h, CA5, biofilm – 48h, LF-89^T, planktonic	-0.458884956	0.060854517	-7.540688548	0.000****
0h, CA5, biofilm – 72h, CA5, planktonic	-0.538403372	0.058602448	-9.187387092	0.000****

0h, CA5, biofilm – 72h, LF-89^T, planktonic	-0.465716	0.060721878	-7.669657406	0.000****
0h, LF-89^T, biofilm – 72h, CA5, biofilm	-0.299043956	0.071486879	-4.183200606	0.000**
0h, LF-89^T, biofilm – 72h, CA5, planktonic	-0.314222979	0.071026417	-4.424029711	0.000**
6h, CA5, biofilm – 24h, CA5, biofilm	0.361971901	0.065769688	5.50362805	0.000****
6h, CA5, biofilm – 0h, CA5, planktonic	0.316797494	0.068465872	4.627086253	0.000***
6h, CA5, biofilm – 0h, LF-89^T, planktonic	0.324357743	0.068047654	4.766626387	0.000***
6h, CA5, biofilm – 24h, CA5, planktonic	0.386044553	0.064138755	6.018896841	0.000****
6h, LF-89^T, biofilm – 72h, CA5, biofilm	-0.293205423	0.071657083	-4.091785625	0.000**
6h, LF-89^T, biofilm – 72h, CA5, planktonic	-0.308384446	0.071197584	-4.331389199	0.000**
12h, CA5,biofilm – 72h, CA5, biofilm	-0.313015583	0.071050794	-4.405518432	0.000**
12h, CA5,biofilm – 72h, CA5, planktonic	-0.328194606	0.070587818	-4.649451069	0.000***
12h, LF-89^T, biofilm – 24h, CA5, biofilm	0.333694076	0.065954592	5.059451728	0.000***
12h, LF-89^T, biofilm – 0h, CA5, planktonic	0.28851967	0.068645145	4.203060098	0.000**
12h, LF-89^T, biofilm – 0h, LF-89^T, planktonic	0.296079919	0.068227728	4.339583435	0.000**
12h, LF-89^T, biofilm – 24h, CA5, planktonic	0.357766729	0.06432762	5.561634816	0.000****
24h, CA5, biofilm – 24h, LF-89^T, biofilm	-0.334642071	0.065951185	-5.074087325	0.000***
24h, CA5, biofilm – 48h, CA5, biofilm	-0.382802652	0.065522714	-5.842289295	0.000****
24h, CA5, biofilm – 48h, LF-89^T, biofilm	-0.37561892	0.065618561	-5.724278493	0.000****
24h, CA5, biofilm – 72h, CA5, biofilm	-0.461969436	0.063704761	-7.251725401	0.000****
24h, CA5, biofilm – 72h, LF-89^T, biofilm	-0.41065656	0.065043817	-6.313537199	0.000****

24h, CA5, biofilm – 6h, CA5, planktonic	-0.349322974	0.06587372	-5.302918577	0.000****
24h, CA5, biofilm – 6h, LF-89^{T}, Planktonic	-0.401324059	0.065223378	-6.153070769	0.000****
24h, CA5, biofilm – 12h, LF-89^{T}, planktonic	-0.298821112	0.065945745	-4.531317529	0.000**
24h, CA5, biofilm – 24h, LF-89^{T}, planktonic	-0.357479224	0.0658106	-5.431939904	0.000****
24h, CA5, biofilm – 48h, CA5, planktonic	-0.369477709	0.065691578	-5.62443045	0.000****
24h, CA5, biofilm – 48h, LF-89^{T}, planktonic	-0.397630044	0.065289114	-6.090296167	0.000****
24h, CA5, biofilm – 72h, CA5, planktonic	-0.47714846	0.063191458	-7.550838	0.000****
24h, CA5, biofilm – 72h, LF-89^{T}, planktonic	-0.404461088	0.06516518	-6.206705633	0.000****
24h, LF-89^{T}, biofilm – 0h, CA5, planktonic	0.289467664	0.068641816	4.217074666	0.000**
24h, LF-89^{T}, biofilm – 0h, LF-89^{T}, planktonic	0.297027913	0.068224389	4.353691072	0.000**
24h, LF-89^{T}, biofilm – 24h, CA5, planktonic	0.358714723	0.06432415	5.576672553	0.000****
CA5,48,Biofilm – 0h, CA5, planktonic	0.337628245	0.068227453	4.948568792	0.000***
48h, CA5, biofilm – 0h, LF-89^{T}, planktonic	0.345188494	0.067807985	5.090676183	0.000****
48h, CA5, biofilm – 24h, CA5, Planktonic	0.406875304	0.063886015	6.368769478	0.000****
48h, LF-89^{T}, biofilm – 0h, CA5, planktonic	0.330444513	0.06831992	4.836722751	0.000***
48h, LF-89^{T}, biofilm – 0h, LF-89^{T}, planktonic	0.338004762	0.067900948	4.977909342	0.000***
48h, LF-89^{T}, biofilm – 24h, CA5, planktonic	0.399691572	0.063984129	6.246729897	0.000****
72h, CA5, biofilm – 0h, CA5, planktonic	0.41679503	0.06647887	6.269586594	0.000****
72h, CA5, biofilm – 0h, LF-89^{T}, planktonic	0.424355279	0.066049144	6.42484149	0.000****
72h, CA5, biofilm – 12h, CA5, planktonic	0.340114231	0.070087303	4.852722461	0.000***

72h, CA5, biofilm – 24h, CA5, planktonic	0.486042088	0.062022182	7.836584747	0.000****
72h, LF-89^T, biofilm – 0h, CA5, planktonic	0.365482154	0.067766059	5.393292139	0.000****
72h, LF-89^T, biofilm – 0h, LF-89^T, planktonic	0.373042403	0.067344012	5.539355202	0.000****
72h, LF-89^T, biofilm – 12h, CA5, planktonic	0.288801355	0.071315047	4.049655262	0.011*
72h, LF-89^T, biofilm – 24h, CA5, planktonic	0.434729213	0.063395477	6.857416833	0.000****
0h, CA5, planktonic – 6h, CA5, planktonic	-0.304148567	0.068566543	-4.435815985	0.000**
0h, CA5, planktonic – 6h, LF-89^T, planktonic	-0.356149653	0.067938963	-5.24220031	0.000****
0h, CA5, planktonic – 12h, LF-89^T, planktonic	-0.253646706	0.06863866	-3.695391285	0.041*
0h, CA5, planktonic – 24h, LF-89^T, planktonic	-0.312304818	0.068505433	-4.558832851	0.000**
0h, CA5, planktonic – 48h, CA5, planktonic	-0.324303303	0.068390407	-4.741941409	0.000***
0h, CA5, planktonic – 48h, LF-89^T, planktonic	-0.352455638	0.068002288	-5.182996763	0.000****
0h, CA5, planktonic – 72h, CA5, planktonic	-0.431974053	0.065986274	-6.546422823	0.000****
0h, CA5, planktonic – 72h, LF-89^T, planktonic	-0.359286682	0.067882912	-5.292741148	0.000****
0h, LF-89^T, planktonic – 6h, CA5, planktonic	-0.311708816	0.06814881	-4.573943667	0.000**
0h, LF-89^T, planktonic – 6h, LF-89^T, planktonic	-0.363709902	0.067517898	-5.386866456	0.000****
0h, LF-89^T, planktonic – 12h, LF-89^T, planktonic	-0.261206955	0.068220835	-3.828844276	0.025*
0h, LF-89^T, planktonic – 24h, LF-89^T, planktonic	-0.319865066	0.068087411	-4.697859172	0.000***
0h, LF-89^T, planktonic – 48h, CA5, planktonic	-0.331863552	0.067971804	-4.882370804	0.000***
0h, LF-89^T, planktonic – 48h, LF-89^T, planktonic	-0.360015886	0.067581579	-5.327130456	0.000****
0h, LF-89^T, planktonic – 72h, CA5, planktonic	-0.439534302	0.065553479	-6.704972904	0.000****

0h, LF-89^T, planktonic – 72h, LF-89^T, planktonic	-0.36684693	0.067461531	-5.437868475	0.000****
6h, CA5, planktonic – 24h, CA5, planktonic	0.373395626	0.064245102	5.812048104	0.000****
6h, LF-89^T, planktonic – 12h, CA5, planktonic	0.279468854	0.071480406	3.909726743	0.019*
6h, LF-89^T, planktonic – 24h, CA5, planktonic	0.425396711	0.063579453	6.690789152	0.000****
12h, CA5, planktonic – 48h, LF-89^T, planktonic	-0.275774838	0.071541008	-3.854779905	0.023*
12h, CA5, planktonic – 72h, CA5, planktonic	-0.355293254	0.06961856	-5.103427244	0.000****
12h, CA5, planktonic – 72h, LF-89^T, planktonic	-0.282605882	0.071426784	-3.956581368	0.015*
12h, LF-89^T, planktonic – 24h, CA5, planktonic	0.322893764	0.064317649	5.02029798	0.000***
24h, CA5, planktonic – 24h, LF-89^T, planktonic	-0.381551876	0.064180591	-5.944972921	0.000****
24h, CA5, planktonic – 48h, CA5, planktonic	-0.393550361	0.06405885	-6.143575194	0.000****
24h, CA5, Planktonic – 48h, LF-89^T, planktonic	-0.421702696	0.063646792	-6.625670878	0.000****
24h, CA5, planktonic – 72h, CA5, planktonic	-0.501221112	0.061495228	-8.150569199	0.000****
24h, CA5, planktonic – 72h, LF-89^T, planktonic	-0.42853374	0.063519829	-6.746456106	0.000****

Supplementary Table S6. Differentially expressed genes (DEGs) in *Piscirickettsia salmonis* LF-89[T] between 24-h and 48-h-old biofilms. Only genes with statistically significant fold-change (FC) values are shown (Padj < 0.05). The $\log_2$ FC value for a specific DEG is written with positive or negative numbers to represent up- or down-regulation in 24-h versus 48-h-old biofilms. A color key for DEGs associated with different virulence categories is represented as follows: ( ) "stress response"; ( ) "iron uptake"; ( ) "endotoxins", and ( ) "other virulence-related genes".

DEG ID	Annotation	$\log_2$ FC
Gene2909	Molecular chaperone DnaK; protein_id=WP_017378060.1;transl_table=11	3.07
Gene1254	Molecular chaperone HtpG; protein_id=WP_017377488.1;transl_table=11	2.21
Gene742	Similar to AA sequence:RefSeq:WP_016209372.1;elongation factor Ts; protein_id=WP_017376218.1	3.12
Gene2879	Similar to AA sequence:RefSeq:WP_004286619.1;elongation factor G; protein_id=WP_017378035.1	2.29
Gene3195	Cell division protein FtsZ; protein_id=WP_017378451.1;transl_table=11	2.23
Gene2041	Hypothetical protein; protein_id=WP_017378201.1;transl_table=11	4.91
Gene332	Hypothetical protein; protein_id=WP_027243188.1;transl_table=11	2.27
Gene277	Hypothetical protein; protein_id=WP_017376843.1;transl_table=11	2.11
Gene2868	50S ribosomal protein L29; protein_id=WP_016209750.1;transl_table=11	5.25

Gene466	50S ribosomal protein L35; protein_id=WP_017378382.1;transl_table=11	4.51
Gene2874	50S ribosomal protein L23; protein_id=WP_016209744.1;transl_table=11	4.17
Gene3019	similar to AA sequence:RefSeq:WP_010548350.1;50S ribosomal protein L33; protein_id=WP_016210730.1	4.09
Gene988	30S ribosomal protein S21; protein_id=WP_016211035.1;transl_table=11	4.05
Gene2860	50S ribosomal protein L18; protein_id=WP_016209757.1;transl_table=11	3.76
Gene2022	Similar to AA sequence:RefSeq:WP_012986941.1;30S ribosomal protein S18;protein_id=WP_016209480.1	3.73
Gene2871	50S ribosomal protein L22; protein_id=WP_016209755.1;transl_table=11	3.49
Gene744	Ribosome recycling factor; protein_id=WP_017376216.1;transl_table=11	3.37
Gene2853	30S ribosomal protein S11; protein_id=WP_016209730.1;transl_table=11	3.20
Gene2872	30S ribosomal protein S19; protein_id=WP_017378032.1;transl_table=11	3.15
Gene2857	50S ribosomal protein L15; protein_id=WP_017378023.1;transl_table=11	3.12

Gene2865	50S ribosomal protein L24; protein_id=WP_016209734.1;transl_table=11	3.07
Gene438	Similar to AA sequence:RefSeq:WP_007148862.1;50S ribosomal protein L21; protein_id=WP_026063730.1	3.07
Gene467	50S ribosomal protein L20; protein_id=WP_016210495.1;transl_table=11	3.04
Gene2861	50S ribosomal protein L6; protein_id=WP_017378025.1;transl_table=11	3.00
Gene2867	Similar to AA sequence:RefSeq:WP_017378029.1;30S ribosomal protein S17;protein_id=WP_017378029.1	2.88
Gene429	50S ribosomal protein L13; protein_id=WP_017378349.1;transl_table=11	2.84
Gene2873	50S ribosomal protein L2; protein_id=WP_017378033.1;transl_table=11	2.77
Gene2880	30S ribosomal protein S7; protein_id=WP_016209732.1;transl_table=11	2.69
Gene2870	30S ribosomal protein S3; protein_id=WP_017378031.1;transl_table=11	2.65
Gene2855	50S ribosomal protein L36; protein_id=WP_016209752.1;transl_table=11	2.49
Gene2866	50S ribosomal protein L14; protein_id=WP_017378028.1;transl_table=11	2.47

Gene2884	50S ribosomal protein L7/L12; protein_id=WP_017378038.1;transl_table=11	2.46
Gene2885	50S ribosomal protein L10; protein_id=WP_017378039.1;transl_table=11	2.35
Gene2869	50S ribosomal protein L16; protein_id=WP_017378030.1;transl_table=11	2.32
Gene2854	30S ribosomal protein S13; protein_id=WP_017378021.1;transl_table=11	2.19
Gene2435	50S ribosomal protein L32; protein_id=WP_016210404.1;transl_table=11	2.14
Gene2023	30S ribosomal protein S6; protein_id=WP_027242778.1;transl_table=11	2.14
Gene2859	30S ribosomal protein S5; protein_id=WP_016209764.1;transl_table=11	2.08
Gene2265	30S ribosomal protein S1; protein_id=WP_017377748.1;transl_table=11	2.06
Gene2852	30S ribosomal protein S4; protein_id=WP_017378020.1;transl_table=11	2.01
Gene1042	Nucleoside-diphosphate kinase; protein_id=WP_017376083.1;transl_table=11	3.91
Gene743	UMP kinase; protein_id=WP_017376217.1;transl_table=11	2.31

Gene1415	Diaminopimelate decarboxylase; protein_id=WP_027242813.1;transl_table=11	3.32
Gene2481	Adenosylmethionine decarboxylase; protein_id=WP_017376335.1;transl_table=11	2.81
Gene2907	4-hydroxy-tetrahydrodipicolinate reductase; protein_id=WP_017378058.1;transl_table=11	3.75
Gene3031	Dihydrofolate reductase; protein_id=WP_017375796.1;transl_table=11	2.84
Gene846	Aldo/keto reductase; protein_id=WP_017377295.1;transl_table=11	2.65
Gene938	Alkyl hydroperoxide reductase (AhpC); protein_id=WP_036773927.1;transl_table=11	2.05
Gene1105	Protease SohB; protein_id=WP_027243145.1;transl_table=11	3.87
Gene2504	PhoH family protein; protein_id=WP_017376019.1;transl_table=11	3.82
Gene12	F0F1 ATP synthase subunit delta; protein_id=WP_017378486.1;transl_table=11	3.56
Gene906	Protein-L-isoaspartate O-methyltransferase; protein_id=WP_017376911.1;transl_table=11	3.13
Gene857	Similar to AA sequence:RefSeq:WP_016209647.1; ATP-dependent Clp endopeptidase proteolytic subunit ClpP; protein_id=WP_016209647.1	2.96
Gene860	Similar to AA sequence:RefSeq:WP_013661710.1;HU family DNA-binding protein; protein_id=WP_016209655.1	2.88
Gene2430	Acyl carrier protein; protein_id=WP_016210413.1;transl_table=11	2.62

Gene280	XRE family transcriptional regulator; protein_id=WP_017376846.1;transl_table=11	2.55
Gene1250	YbaB/EbfC family nucleoid-associated protein; protein_id=WP_016209821.1;transl_table=11	2.48
Gene1792	MexH family multidrug efflux RND transporter periplasmic adaptor subunit; protein_id=WP_027242910.1;transl_table=11	2.29
Gene3059	Similar to AA sequence:RefSeq:WP_017377545.1;succinylglutamate desuccinylase; protein_id=WP_017377545.1	2.17
Gene1436	Similar to AA sequence:RefSeq:WP_017377442.1;tRNA (guanosine(37)-N1)-methyltransferase TrmD; protein_id=WP_017377442.1	2.10
Gene1702	Sodium:dicarboxylate symporter; protein_id=WP_027243044.1;transl_table=11	2.07
Gene3024	Sulfite exporter TauE/SafE family protein; protein_id=WP_017375950.1;transl_table=11	2.05
Gene356	LbtU family siderophore porin; protein_id=WP_027243075.1;transl_table=11	2.03
Gene2278	WD40 repeat domain-containing protein; protein_id=WP_017375702.1;transl_table=11	2.02
Gene1561	Hypothetical protein; protein_id=WP_017376485.1;transl_table=11	-2.61
Gene138	Hypothetical protein; protein_id=WP_047927421.1;transl_table=11	-2.61

Gene1857	3-hydroxyacyl-CoA dehydrogenase/enoyl-CoA hydratase family protein ; protein_id=WP_017377952.1;transl_table=11	-2.18
Gene37	CBS domain-containing protein; protein_id=WP_026063559.1;transl_table=11	-2.63
Gene1361	CBS domain-containing protein; protein_id=WP_017378213.1;transl_table=11	-2.07
Gene1213	TIGR01777 family protein; protein_id=WP_017376415.1;transl_table=11	-3.11
Gene1543	Protein phosphatase CheZ; protein_id=WP_016209769.1;transl_table=11	-2.72
Gene2447	STAS domain-containing protein; protein_id=WP_017376372.1;transl_table=11	-2.58
Gene2450	ABC transporter permease; protein_id=WP_017376369.1;transl_table=11	-2.53
Gene817	Similar to AA sequence:RefSeq:WP_009814112.1;3-methyl-2-oxobutanoate hydroxymethyltransferase; protein_id=WP_026063614.1	-2.34
Gene2587	Sulfurtransferase TusA family protein; protein_id=WP_027242612.1;transl_table=11	-2.32
Gene1750	Methyltransferase domain-containing protein; protein_id=WP_017376769.1;transl_table=11	-2.32
Gene1529	Class I SAM-dependent methyltransferase; protein_id=WP_048875999.1;transl_table=11	-2.25

Gene1	Similar to AA sequence:RefSeq:WP_017378476.1;ribonuclease P protein component; protein_id=WP_017378476.1	-2.15
Gene1732	Similar to AA sequence:RefSeq:WP_020481309.1;cytochrome d terminal oxidase subunit I; protein_id=WP_027243221.1	-2.14
Gene3376	IS6 family transposase; protein_id=WP_048876210.1;transl_table=11	-2.04
Gene1880	Undecaprenyl-diphosphate phosphatase; protein_id=WP_017376716.1;transl_table=11	-2.13

Supplementary Table S7. Differentially expressed genes (DEGs) in *Piscirickettsia salmonis* LF-89[T] between 24-h and 48-h-old planktonic bacteria. Only genes with statistically significant fold-change (FC) values are shown (Padj < 0.05). The $\log_2$ FC value for a specific DEG is written with positive or negative numbers to represent up- or down-regulation in planktonic bacteria at 24 h versus planktonic bacteria at 48 h. A color key for DEGs associated with different virulence categories is represented as follows: ( ) "stress response"; ( ) "iron uptake", and ( ) "other virulence-related genes".

DEG ID	Annotation	$\log_2$ FC
Gene2451	Similar to AA sequence:RefSeq:WP_016209539.1;ABC transporter ATP-binding protein; protein_id=WP_016209539.1	2.81
Gene1082	ABC transporter permease; protein_id=WP_017377874.1;transl_table=11	2.59
Gene1266	Similar to AA sequence:RefSeq:WP_016211115.1;ABC transporter ATPase; protein_id=WP_027242809.1	2.20
Gene2264	DUF1049 domain-containing protein; protein_id=WP_017377747.1;transl_table=11	2.52
Gene2136	Helix-turn-helix domain-containing protein [Piscirickettsia salmonis]; protein_id=WP_017376289.1;transl_table=11	4.38
Gene1997	Hypothetical protein;protein_id=WP_027242770.1;transl_table=11	3.42
Gene1857	3-hydroxyacyl-CoA dehydrogenase/enoyl-CoA hydratase family protein [Piscirickettsia salmonis]; protein_id=WP_017377952.1;transl_table=11	3.33
Gene2834	Transporter substrate-binding domain-containing protein [Piscirickettsia salmonis]; protein_id=WP_027242668.1;transl_table=11	2.67
Gene138	Hypothetical protein;protein_id=WP_047927421.1;transl_table=11	2.63

Gene2403	4-hydroxybenzoate octaprenyltransferase; protein_id=WP_017377937.1;transl_table=11	3.69
Gene2673	Prolipoprotein diacylglyceryl transferase; protein_id=WP_017375811.1;transl_table=11	2.88
Gene2445	Similar to AA sequence:RefSeq:WP_016209518.1;UDP-N-acetylglucosamine 1-carboxyvinyltransferase; protein_id=WP_016209518.1	2.82
Gene2502	Ion transporter; protein_id=WP_017376017.1;transl_table=11	2.17
Gene1336	FAD-dependent oxidoreductase; protein_id=WP_017378240.1;transl_table=11	2.71
Gene1431	Gene=*fadH*; similar to AA sequence:RefSeq:WP_013790923.1; NADPH-dependent 2,4-dienoyl-CoA reductase	2.41
Gene1064	Phosphatase PAP2 family protein; protein_id=WP_017377894.1;transl_table=11	2.62
Gene1732	Similar to AA sequence:RefSeq:WP_020481309.1;cytochrome d terminal oxidase subunit I; protein_id=WP_027243221.1	2.60
Gene1140	Homoserine kinase; protein_id=WP_017377783.1;transl_table=11	2.59
Gene1487	Universal stress protein (USP); protein_id=WP_036816928.1;transl_table=11	2.57
Gene1083	Gamma-glutamylcyclotransferase; protein_id=WP_017377873.1;transl_table=11	2.45

Gene220	Similar to AA sequence:RefSeq:WP_016209852.1;acetyl-CoA carboxylase biotin carboxyl carrier protein; protein_id=WP_017377615.1	2.40
Gene2587	Sulfurtransferase TusA family protein; protein_id=WP_027242612.1;transl_table=11	2.40
Gene2568	Quinone-dependent dihydroorotate dehydrogenase; protein_id=WP_017377044.1;transl_table=11	2.35
Gene574	Porin family protein; protein_id=WP_017377818.1;transl_table=11	2.29
Gene1137	Similar to AA sequence:RefSeq:WP_016210013.1;long-chain-fatty-acid--CoA ligase; protein_id=WP_027242896.1	2.16
Gene3113	Similar to AA sequence:RefSeq:WP_016209697.1;type 1 glutamine amidotransferase; protein_id=WP_017377373.1	2.14
Gene447	Glycosyl hydrolase family 1;protein_id=WP_036772406.1;transl_table=11	2.12
Gene7	Chromosome partitioning protein ParB; protein_id=WP_027242747.1;transl_table=11	2.11
Gene2922	Similar to AA sequence:RefSeq:WP_017378073.1;glutaminase A; protein_id=WP_017378073.1	2.10
Gene522	DoxX family protein; protein_id=WP_017377263.1;transl_table=11	2.09
Gene627	Protein motif:HMM:TIGR00492;alanine racemase; protein_id=WP_027243040.1	2.07

Gene1529	Class I SAM-dependent methyltransferase; protein_id=WP_048875999.1;transl_table=11	2.05
Gene3089	Conjugal transfer protein TrbE; protein_id=WP_027242714.1;transl_table=11	2.04
Gene1739	DUF4442 domain-containing protein; protein_id=WP_026063485.1;transl_table=11	2.51
Gene2860	50S ribosomal protein L18; protein_id=WP_016209757.1;transl_table=11	-2.39
Gene2886	50S ribosomal protein L1; protein_id=WP_017378040.1;transl_table=11	-2.82
Gene2871	50S ribosomal protein L22; protein_id=WP_016209755.1;transl_table=11	-2.57
Gene467	50S ribosomal protein L20; protein_id=WP_016210495.1;transl_table=11	-2.28
Gene2880	30S ribosomal protein S7; protein_id=WP_016209732.1;transl_table=11	-2.48
Gene1353	Similar to AA sequence:RefSeq:WP_017378221.1;phosphatidylserine decarboxylase; protein_id=WP_017378221.1	-2.15
Gene1415	Diaminopimelate decarboxylase; protein_id=WP_027242813.1;transl_table=11	-3.73
Gene3136	Glycine--tRNA ligase subunit beta; protein_id=WP_017378400.1;transl_table=11	-2.43
Gene118	Cold-shock protein; protein_id=WP_016209597.1;transl_table=11	-2.40

Gene856	Trigger factor; protein_id=WP_017377304.1;transl_table=11	-2.33
Gene2278	WD40 repeat domain-containing protein; protein_id=WP_017375702.1;transl_table=11	-2.09
Gene3181	Enoyl-[acyl-carrier-protein] reductase FabI; protein_id=WP_017378439.1;transl_table=11	-2.82

Supplementary Table S8. Differentially expressed genes (DEGs) in *Piscirickettsia salmonis* LF-89^T between 24-h-old biofilms and the planktonic counterpart. Only genes with statistically significant fold-change (FC) values are shown (Padj < 0.05). The $\log_2$ FC value for a specific DEG is written with positive or negative numbers to represent up- or down-regulation in 24-h-old biofilms versus planktonic bacteria at 24 h. A color key for DEGs associated with different virulence categories is represented as follows: ( ) "stress response"; ( ) "iron uptake"; ( ) "endotoxins", and ( ) "other virulence-related genes".

DEG ID	Annotation	Log_2 FC
Gene2861	50S ribosomal protein L6;protein_id=WP_017378025.1;transl_table=11	2.08
Gene2868	50S ribosomal protein L29;protein_id=WP_016209750.1;transl_table=11	3.05
Gene2871	50S ribosomal protein L22;protein_id=WP_016209755.1;transl_table=11	2.98
Gene2874	50S ribosomal protein L23;protein_id=WP_016209744.1;transl_table=11	2.34
Gene2886	50S ribosomal protein L1;protein_id=WP_017378040.1;transl_table=11	2.03
Gene1931	Hypothetical protein; protein_id=WP_075275336.1;transl_table=11	4.50
Gene3182	Hypothetical protein; protein_id=WP_017378440.1;transl_table=11	2.82
Gene361	Hypothetical protein; protein_id=WP_036772686.1;transl_table=11	2.10
Gene370	L,D-transpeptidase family protein; protein_id=WP_017377649.1;transl_table=11	2.21

Gene3031	Dihydrofolate reductase; protein_id=WP_017375796.1;transl_table=11	2.51
Gene3181	Enoyl-[acyl-carrier-protein] reductase FabI; protein_id=WP_017378439.1;transl_table=11	2.88
Gene846	Aldo/keto reductase; protein_id=WP_017377295.1;transl_table=11	2.54
Gene105	Similar to AA sequence:RefSeq:WP_017376187.1;dTDP-glucose 4,6-dehydratase;protein_id=WP_017376187.1	4.12
Gene1415	Diaminopimelate decarboxylase; protein_id=WP_027242813.1;transl_table=11	3.59
Gene2179	Fusaric acid resistance family protein; protein_id=WP_036771855.1;transl_table=11	2.58
Gene2278	WD40 repeat domain-containing protein; protein_id=WP_017375702.1;transl_table=11	2.27
Gene280	XRE family transcriptional regulator; protein_id=WP_017376846.1;transl_table=11	2.32
Gene2910	Nucleotide exchange factor GrpE; protein_id=WP_048875858.1;transl_table=11	3.44
Gene3059	Similar to AA sequence:RefSeq:WP_017377545.1;succinylglutamate desuccinylase; protein_id=WP_017377545.1	2.04
Gene358	MFS transporter; protein_id=WP_027243073.1;transl_table=11	2.59
Gene434	YraN family protein; protein_id=WP_027242730.1;transl_table=11	2.14

Gene67	Short-chain dehydrogenase; protein_id=WP_036772645.1;transl_table=11	2.02
Gene1702	Sodium:dicarboxylate symporter; protein_id=WP_027243044.1;transl_table=11	2.56
Gene1857	3-hydroxyacyl-CoA dehydrogenase/enoyl-CoA hydratase family protein; protein_id=WP_017377952.1;transl_table=11	-2.36
Gene2024	Transporter substrate-binding domain-containing protein; protein_id=WP_027242779.1;transl_table=11	-2.88
Gene2136	Helix-turn-helix domain-containing protein; protein_id=WP_017376289.1;transl_table=11	-4.48
Gene2834	Transporter substrate-binding domain-containing protein; protein_id=WP_027242668.1;transl_table=11	-2.72
Gene3263	Tyrosine-type recombinase/integrase; protein_id=WP_036774388.1;transl_table=11	-2.40
Gene3526	Transposase [Piscirickettsia salmonis]; protein_id=WP_048876256.1;transl_table=11	-2.25
Gene1140	Homoserine kinase; protein_id=WP_017377783.1;transl_table=11	-2.04
Gene1213	TIGR01777 family protein; protein_id=WP_017376415.1;transl_table=11	-2.71
Gene1431	Gene=*fadH*;similar to AA sequence:RefSeq:WP_013790923.1; NADPH-dependent 2,4-dienoyl-CoA reductase	-2.29

Gene1487	Universal stress protein (USP); protein_id=WP_036816928.1;transl_table=11	-3.27
Gene1529	Class I SAM-dependent methyltransferase; protein_id=WP_048875999.1;transl_table=11	-2.42
Gene1732	Similar to AA sequence:RefSeq:WP_020481309.1;cytochrome d terminal oxidase subunit I; protein_id=WP_027243221.1	-2.59
Gene2587	Sulfurtransferase TusA family protein; protein_id=WP_027242612.1;transl_table=11	-2.17
Gene37	CBS domain-containing protein;protein_id=WP_026063559.1;transl_table=11	-2.87
Gene817	Similar to AA sequence:RefSeq:WP_009814112.1;3-methyl-2-oxobutanoate hydroxymethyltransferase; protein_id=WP_026063614.1	-2.37

Supplementary Table S9. Differentially expressed genes (DEGs) in *Piscirickettsia salmonis* LF-89^{T} between 48-h-old biofilms and the planktonic counterpart. Only genes with statistically significant fold-change (FC) values are shown (Padj < 0.05). The $\log_2$ FC value for a specific DEG is written with positive or negative numbers to represent up- or down-regulation in 48-h-old biofilms versus planktonic bacteria at 48 h. A color key for DEGs associated with different virulence categories is represented as follows: ( ) "stress response"; ( ) "iron uptake", and ( ) "other virulence-related genes".

DEG ID	Annotation	$\log_2$ FC
Gene1361	CBS domain-containing protein; protein_id=WP_017378213.1;transl_table=11	2.39
Gene1266	Similar to AA sequence:RefSeq:WP_016211115.1;ABC transporter ATPase; protein_id=WP_027242809.1	2.05
Gene2451	Similar to AA sequence:RefSeq:WP_016209539.1;ABC transporter ATP-binding protein; protein_id=WP_016209539.1	2.82
Gene3146	Potassium transporter; protein_id=WP_080963575.1;transl_table=11	2.50
Gene1226	Cadherin-like domain-containing protein; protein_id=WP_048875975.1;transl_table=11	2.03
Gene1313	TraM recognition domain-containing protein; protein_id=WP_017378263.1;transl_table=11	3.64
Gene1857	3-hydroxyacyl-CoA dehydrogenase/enoyl-CoA hydratase family protein; protein_id=WP_017377952.1;transl_table=11	3.15
Gene1863	Hypothetical protein; protein_id=WP_017375861.1;transl_table=11	2.84
Gene2136	Helix-turn-helix domain-containing protein; protein_id=WP_017376289.1;transl_table=11	4.56

Gene2163	SufE family protein; protein_id=WP_017377023.1;transl_table=11	2.47
Gene3263	Tyrosine-type recombinase/integrase; protein_id=WP_036774388.1;transl_table=11	2.21
Gene3526	Transposase [Piscirickettsia salmonis]; protein_id=WP_048876256.1;transl_table=11	2.88
Gene629	6-carboxytetrahydropterin synthase; protein_id=WP_017376597.1;transl_table=11	2.11
Gene686	Transporter substrate-binding domain-containing protein; protein_id=WP_017377734.1;transl_table=11	2.98
Gene1739	DUF4442 domain-containing protein; protein_id=WP_026063485.1;transl_table=11	2.23
Gene2264	DUF1049 domain-containing protein; protein_id=WP_017377747.1;transl_table=11	2.67
Gene2568	Quinone-dependent dihydroorotate dehydrogenase; protein_id=WP_017377044.1;transl_table=11	2.19
Gene1732	Similar to AA sequence:RefSeq:WP_020481309.1;cytochrome d terminal oxidase subunit I; protein_id=WP_027243221.1	2.15
Gene1061	Response regulator; protein_id=WP_017377897.1;transl_table=11	2.31
Gene1140	Homoserine kinase; protein_id=WP_017377783.1;transl_table=11	2.45
Gene1336	FAD-dependent oxidoreductase; protein_id=WP_017378240.1;transl_table=11	2.21

Gene220	Similar to AA sequence:RefSeq:WP_016209852.1;acetyl-CoA carboxylase biotin carboxyl carrier protein; protein_id=WP_017377615.1	2.04
Gene2402	Bifunctional [glutamate--ammonia ligase]-adenylyl-L-tyrosine phosphorylase/[glutamate--ammonia-ligase] adenylyltransferase; protein_id=WP_048876070.1;transl_table=11	2.02
Gene2403	4-hydroxybenzoate octaprenyltransferase; protein_id=WP_017377937.1;transl_table=11	3.82
Gene2447	STAS domain-containing protein; protein_id=WP_017376372.1;transl_table=11	2.28
Gene2587	Sulfurtransferase TusA family protein; protein_id=WP_027242612.1;transl_table=11	2.55
Gene2601	N-acetyltransferase; protein_id=WP_017377072.1;transl_table=11	2.00
Gene2673	Prolipoprotein diacylglyceryl transferase; protein_id=WP_017375811.1;transl_table=11	2.27
Gene2922	Similar to AA sequence:RefSeq:WP_017378073.1;glutaminase A; protein_id=WP_017378073.1	2.05
Gene3089	Conjugal transfer protein TrbE; protein_id=WP_027242714.1;transl_table=11	2.07
Gene3315	Phage portal protein; protein_id=WP_087910668.1;transl_table=11	5.23
Gene627	Protein motif:HMM:TIGR00492;alanine racemase; protein_id=WP_027243040.1	2.26

Gene148	LbtU family siderophore porin; protein_id=WP_017376146.1;transl_table=11	-2.85
Gene2041	Hypothetical protein; protein_id=WP_017378201.1;transl_table=11	-4.72
Gene206	YhbY family RNA-binding protein; protein_id=WP_017377601.1;transl_table=11	-2.19
Gene1434	30S ribosomal protein S16;protein_id=WP_016211283.1;transl_table=11	-2.12
Gene2023	30S ribosomal protein S6;protein_id=WP_027242778.1;transl_table=11	-2.03
Gene2265	30S ribosomal protein S1;protein_id=WP_017377748.1;transl_table=11	-2.17
Gene2852	30S ribosomal protein S4;protein_id=WP_017378020.1;transl_table=11	-2.02
Gene2853	30S ribosomal protein S11;protein_id=WP_016209730.1;transl_table=11	-3.35
Gene2854	30S ribosomal protein S13;protein_id=WP_017378021.1;transl_table=11	-2.90
Gene2859	30S ribosomal protein S5;protein_id=WP_016209764.1;transl_table=11	-2.58
Gene2863	30S ribosomal protein S14;protein_id=WP_026063699.1;transl_table=11	-2.26
Gene2870	30S ribosomal protein S3;protein_id=WP_017378031.1;transl_table=11	-2.95
Gene2880	30S ribosomal protein S7;protein_id=WP_016209732.1;transl_table=11	-3.25
Gene2952	30S ribosomal protein S15;protein_id=WP_016209298.1;transl_table=11	-2.15

Gene988	30S ribosomal protein S21;protein_id=WP_016211035.1;transl_table=11	-3.43
Gene2867	Similar to AA sequence:RefSeq:WP_017378029.1;30S ribosomal protein S17;protein_id=WP_017378029.1	-2.61
Gene2022	Similar to AA sequence:RefSeq:WP_012986941.1;30S ribosomal protein S18;protein_id=WP_016209480.1	-3.78
Gene2435	50S ribosomal protein L32; protein_id=WP_016210404.1;transl_table=11	-2.77
Gene2855	50S ribosomal protein L36;protein_id=WP_016209752.1;transl_table=11	-2.04
Gene2857	50S ribosomal protein L15;protein_id=WP_017378023.1;transl_table=11	-3.14
Gene2860	50S ribosomal protein L18;protein_id=WP_016209757.1;transl_table=11	-4.18
Gene2861	50S ribosomal protein L6;protein_id=WP_017378025.1;transl_table=11	-2.90
Gene2865	50S ribosomal protein L24;protein_id=WP_016209734.1;transl_table=11	-3.69
Gene2866	50S ribosomal protein L14;protein_id=WP_017378028.1;transl_table=11	-2.35
Gene2868	50S ribosomal protein L29;protein_id=WP_016209750.1;transl_table=11	-4.20

Gene2871	50S ribosomal protein L22;protein_id=WP_016209755.1;transl_table=11	-3.08
Gene2873	50S ribosomal protein L2;protein_id=WP_017378033.1;transl_table=11	-2.59
Gene2874	50S ribosomal protein L23;protein_id=WP_016209744.1;transl_table=11	-3.05
Gene2884	50S ribosomal protein L7/L12;protein_id=WP_017378038.1;transl_table=11	-2.65
Gene2885	50S ribosomal protein L10;protein_id=WP_017378039.1;transl_table=11	-2.71
Gene2886	50S ribosomal protein L1;protein_id=WP_017378040.1;transl_table=11	-2.63
Gene2991	50S ribosomal protein L31;protein_id=WP_017378137.1;transl_table=11	-2.72
Gene3018	50S ribosomal protein L28;protein_id=WP_017375944.1;transl_table=11	-2.36
Gene429	50S ribosomal protein L13;protein_id=WP_017378349.1;transl_table=11	-2.49
Gene466	50S ribosomal protein L35;protein_id=WP_017378382.1;transl_table=11	-4.54
Gene467	50S ribosomal protein L20;protein_id=WP_016210495.1;transl_table=11	-4.17

Gene438	Similar to AA sequence:RefSeq:WP_007148862.1;50S ribosomal protein L21;protein_id=WP_026063730.1	-2.78
Gene2887	Similar to AA sequence:RefSeq:WP_008045226.1;50S ribosomal protein L11;protein_id=WP_017378041.1	-2.71
Gene3019	Similar to AA sequence:RefSeq:WP_010548350.1;50S ribosomal protein L33;protein_id=WP_016210730.1	-4.37
Gene3195	Cell division protein FtsZ; protein_id=WP_017378451.1;transl_table=11	-2.10
Gene3196	Similar to AA sequence:RefSeq:WP_016209355.1;cell division protein FtsA; protein_id=WP_016209355.1	-2.47
Gene2879	Similar to AA sequence:RefSeq:WP_004286619.1;elongation factor G; protein_id=WP_017378035.1	-2.38
Gene742	Similar to AA sequence:RefSeq:WP_016209372.1;elongation factor Ts; protein_id=WP_017376218.1	-2.45
Gene1254	Molecular chaperone HtpG; protein_id=WP_017377488.1;transl_table=11	-3.04
Gene217	Similar to AA sequence:RefSeq:WP_017377613.1;chaperonin GroEL; protein_id=WP_017377613.1	-2.63
Gene218	Co-chaperone GroES; protein_id=WP_016209850.1;transl_table=11	-2.34
Gene2909	Molecular chaperone DnaK; protein_id=WP_017378060.1;transl_table=11	-3.83

Gene1042	Nucleoside-diphosphate kinase; protein_id=WP_017376083.1;transl_table=11	-2.72
Gene542	Similar to AA sequence:RefSeq:WP_017376007.1;pyruvate kinase; protein_id=WP_017376007.1	-2.15
Gene743	UMP kinase; protein_id=WP_017376217.1;transl_table=11	-2.02
Gene1353	Similar to AA sequence:RefSeq:WP_017378221.1;phosphatidylserine decarboxylase; protein_id=WP_017378221.1	-2.26
Gene1415	Diaminopimelate decarboxylase; protein_id=WP_027242813.1;transl_table=11	-3.45
Gene2481	Adenosylmethionine decarboxylase; protein_id=WP_017376335.1;transl_table=11	-2.79
Gene1063	Ribonuclease; protein_id=WP_027242839.1;transl_table=11	-2.04
Gene1070	Similar to AA sequence:RefSeq:WP_017377888.1;protein TolQ; protein_id=WP_017377888.1	-3.67
Gene1436	Similar to AA sequence:RefSeq:WP_017377442.1;tRNA (guanosine(37)-N1)-methyltransferase TrmD; protein_id=WP_017377442.1	-2.16
Gene2504	PhoH family protein; protein_id=WP_017376019.1;transl_table=11	-2.89
Gene2832	Prokaryotic *dksA*/traR C4-type zinc finger family protein; protein_id=WP_016210574.1;transl_table=11	-2.06

Gene2910	Nucleotide exchange factor GrpE; protein_id=WP_048875858.1;transl_table=11	-2.64
Gene3049	Similar to AA sequence:RefSeq:WP_014980177.1;glutamate--ammonia ligase; protein_id=WP_016210041.1	-2.18
Gene431	Stringent starvation protein A (SspA); protein_id=WP_017378351.1;transl_table=11	-3.36
Gene856	Trigger factor; protein_id=WP_017377304.1;transl_table=11	-2.66
Gene906	Protein-L-isoaspartate O-methyltransferase; protein_id=WP_017376911.1;transl_table=11	-3.07
Gene938	Alkyl hydroperoxide reductase (AhpC); protein_id=WP_036773927.1;transl_table=11	-2.03
